# Global, regional, and national assessment of foreign body aspiration (1990–2021): novel insights into incidence, mortality, and disability-adjusted life years

**DOI:** 10.1186/s13049-025-01352-z

**Published:** 2025-03-11

**Authors:** Pingping Zheng, Ning Zhang, Zixi Chen, Zhelong Jiang

**Affiliations:** 1 Department of Emergency, The Affiliated Hangzhou First People’s Hospital, College of Medicine, Westlake University, No. 261 Huanshan Road, Shangcheng District, Hangzhou, Zhejiang Province 310006 PR China; 2 Department of Cardiology, College of Medicine, The Affiliated Hangzhou First People’s Hospital, Westlake University, No. 261 Huanshan Road, Shangcheng District, Hangzhou, Zhejiang Province 310006 PR China

**Keywords:** Foreign body aspiration, Epidemiology, Global burden of disease, Sociodemographic index, Prevention

## Abstract

**Background:**

Foreign body aspiration (FBA) is a preventable yet underrecognized global health challenge, contributing to substantial clinical and economic burdens. Comprehensive and comparable analyses of FBA trends across diverse populations and socioeconomic contexts remain limited. Leveraging data from the 2021 Global Burden of Disease (GBD) Study, we provide an in-depth global, regional, and national analysis of FBA trends over the past three decades, including the first evaluation of disability-adjusted life years (DALYs), years of life lost (YLLs), and years lived with disability (YLDs).

**Methods:**

We examined FBA incidence, mortality, and disease burden across regions, nations, ages, sexes, and Socio Demographic Index (SDI) levels from 1990 to 2021, calculating age-standardized incidence (ASIR) and death (ASDR) rates, as well as estimated annual percentage changes (EAPCs).

**Results:**

Globally, FBA incidence declined by 35.3% between 1990 and 2021 (EAPC: -2.02; 95% CI: -2.13 to -1.91), with marked reductions among children under 5 years of age. Nonetheless, total FBA-related deaths rose slightly from 99,329 (95% UI: 80,764–112,381) in 1990 to 103,915 (95% UI: 82,081–113,555) in 2021. While many regions showed improvement, countries such as Italy, Georgia, and Zimbabwe recorded increases in ASIRs. In 2021, children under 5 remained at highest risk of morbidity, while older adults (≥ 70 years), especially in high-income Asia Pacific and Western Europe, showed elevated mortality. Notably, younger children achieved substantial decreases in incidence, death, and DALYs, yet older populations faced modest rises in mortality and DALYs. Higher-SDI regions reported the greatest morbidity and mortality, and high-middle SDI regions exhibited the highest DALYs, YLLs, and YLDs, reflecting the influence of socioeconomic development on FBA burden.

**Conclusions:**

Global FBA incidence declined from 1990 to 2021, yet the number of associated deaths continued to rise, indicating ongoing challenges in prevention and management. High- and middle-high SDI regions carried the greatest burden, with children under 5 and older adults (≥ 70 years) particularly affected. These patterns suggest that both advancing socioeconomic development and population aging influence FBA outcomes. Strengthening surveillance, improving emergency response, and implementing targeted, population-specific prevention strategies are essential for reducing the global FBA burden.

**Supplementary Information:**

The online version contains supplementary material available at 10.1186/s13049-025-01352-z.

## Introduction

Foreign body aspiration (FBA), also defined as choking, is a potentially life-threatening emergency that predominantly affects young children and the elderly [1]. Outcomes can range from unnoticed, asymptomatic episodes to complete airway obstruction, leading to acute asphyxia, hypoxic brain injury, or death [2]. Prompt recognition and timely management are essential to mitigate severe complications. Despite advancements in prevention and treatment, FBA remains a significant global health challenge, imposing considerable social and economic burdens. In 1982, the incidence of choking in the United States was reported at 0.66 per 100,000 individuals annually [3]. In 2016, London recorded 1,916 cases of significant choking requiring emergency assessment [4], and more than 10,000 annual emergency department visits were attributed to choking in children aged 14 years and under [1]. Among adults, the incidence of choking increases with advancing age [4]. The National Safety Council identified choking as the third leading cause of unintentional death in the United States in 2018 [5] and the second leading cause in Japan in 2019 [6]. Similarly, between 2006 and 2012, Canada experienced a rise in choking-related deaths among children compared to previous decades [7]. In the United Kingdom, the Office for National Statistics reported 289 choking-related deaths in 2016, a 17% increase from the previous year [1].

The economic burden is equally alarming. Between 2000 and 2009, healthcare costs associated with FBA in children in the United States increased from $93 million to $486 million [8]. However, existing studies are often limited by small sample sizes and short observation periods, which constrain the generalizability of findings [9–11].Comprehensive and timely epidemiological data are essential to inform effective health policy and improve healthcare systems to address this burden adequately.

This study leverages data from the Global Burden of Disease Study 2021 to analyze the morbidity, mortality, and disability-adjusted life years (DALYs) associated with FBA across regions, countries, age groups, genders, and the sociodemographic index. By providing a detailed global perspective, this study aims to inform the design of effective prevention and management strategies to reduce the burden of FBA.

## Methods

### Data source

The Global Burden of Disease Study 2021 (GBD 2021) provides comprehensive estimates of mortality and morbidity trends for 371 diseases and injuries across 204 countries and territories from 1990 to 2021 [12, 13]. The dataset, which is publicly accessible online (http://www.ghdx.healthdata.org), was utilized to extract information on pulmonary aspiration and foreign body in the airway. Our analysis focused on incidence, mortality, DALYs, years of life lost (YLLs) and years lived with disability (YLDs) across different age groups, genders (males, females, and combined), and regions. This study adheres to the Guidelines for Accurate and Transparent Health Estimates Reporting (GATHER) statement [14], and registered in the GBD 2021 Paper Proposal Form (https://uwhealthmetrics.co1.qualtrics.com/).

## Definition

### Case definition

The GBD 2021 case definition relies on International Classification of Diseases, Ninth Revision (ICD-9) [15], and Tenth Revision (ICD-10) codes [16], which vary depending on the data source and time period [12]. “Pulmonary aspiration and foreign body in airway” encompass unintentional deaths or disabilities resulting from the inhalation, swallowing, or aspiration of extraneous material entering the airway or lungs. Relevant ICD-9 codes include 770.1–770.18, E911–E912.09, E913.19, and E913.8–E913.99, while ICD-10 codes cover W75–W76.9, W78–W80.9, and W83–W84.9 (S1 Table). Data for FBA in GBD 2021 are derived from 2,574 data sources (https://ghdx.healthdata.org/gbd-2021/sources).

### DALYs, YLLs and YLDs

DALYs quantify the economic burden of disease due to productivity loss. DALYs are calculated as the sum of YLLs due to premature death and YLDs [17]. YLLs are derived as the product of deaths in each age and sex group and corresponding life expectancy, according to the formula:

YLLs = ∑ (life expectancy − age of death) ×number of deaths [18].

YLDs are calculated by multiplying the prevalence of disease sequelae by disability weights, which range from 0 (perfect health) to 1 (death) [17].

### Socio-demographic index (SDI)

The SDI is a composite indicator representing a country or region’s health-related social and economic status. It is calculated based on per capita income, total fertility rate (age < 25 years), and average educational attainment (age ≥ 15 years), ranging from 0 (minimum development) to 1 (maximum development) [17]. Countries in the GBD study are grouped into five SDI categories: low (0–0.45), low-middle (0.45–0.61), middle (0.61–0.69), high-middle (0.69–0.81), and high (0.81–1) [19]. SDI data can be accessed at https://ghdx.healthdata.org/record/global-burden-disease-study-2021-gbd-2021-socio-demographic-index-sdi-1950–2021.

### Data analysis

This study quantified the burden of FBA by computing the number and age-standardized rates (ASRs) of incidence, mortality, and DALYs. The 95% uncertainty intervals (UIs) were reported for each metric, defined as the 25th and 975th values of the ordered 1,000 draws. ASRs were standardized to the global age structure to account for differences in age composition across regions.

To assess temporal trends, the estimated annual percentage change (EAPC) in ASR between 1990 and 2021 was calculated using the formula:

EAPC = 100 × (exp(β) − 1).

where β represents the slope of the natural logarithm of the ASR over time, obtained through a linear regression model. A positive EAPC signifies an average annual percentage increase in the ASR, whereas a negative EAPC indicates a decline. An EAPC of 0 suggests that the ASR remains stable throughout the study period. The larger the absolute value of the EAPC, the more pronounced the rate of change over time [12].

Additionally, we analyzed the relationship between SDI and the burden of FBA to explore socio-economic disparities. All analyses and visualizations were conducted using the R software package (version 4.2.3) and JD_GBDR (V2.22, Jingding Medical Technology Co., Ltd.)

## Results

### Global and regional incidence

Globally, incident cases of FBA declined by 35.3%, from 1,920,812 (95% UI: 1,451,855–2,504,134) in 1990 to 1,243,442 (95% UI: 992,477–1,551,580) in 2021. The age-standardized incidence rate (ASIR) also decreased significantly over the same period, from 32.7 per 100,000 (95% UI: 25.14–42.27) to 17.31 per 100,000 (95% UI: 13.71–21.77), corresponding to an estimated annual percentage change (EAPC) of -2.02 (95% CI: -2.13 to -1.91) (Table 1).

**Table 1:** Incident cases and ASIR^a^ of foreign body aspiration in 1990 and 2021, and Temporal trends

	1990		2021		1990–2021 EAPC^b^(95%CI^d^)
	Incident cases (95%UI ^c^)	ASIR^a^ per 100,000 (95% UI ^c^)	Incident cases (95%UI ^c^)	ASIR^a^ per 100,000 (95% UI^c^)	
Global	1,920,812(1451855–2504134)	32.74(25.14–42.27)	1,243,442(992477–1551580)	17.31(13.73–21.77)	-2.02(-2.13 to-1.91)
Sex					
Males	954,772(725324–1235707)	31.95(24.68–40.81)	611,888(493046–764160)	16.86(13.50-21.12)	-2.06(-2.16 to-1.96)
Females	966,040(731089–1260093)	33.64(25.61–43.44)	631,554(502133–792021)	17.87(14.02–22.57)	-1.97(-2.09 to-1.85)
Socio-demographic index					
High SDI^e^	361,940(288916–451948)	49.03(38.20-62.92)	272,907(229667–325317)	30.16(23.87–38.30)	-1.49(-1.59 to-1.39)
High-middle SDI^e^	449,697(349148–573566)	45.61(35.13–58.73)	218,128(177776–268840)	13.64(10.44–17.84)	-2.02(-2.13 to-1.92)
Middle SDI^e^	547,162(410037–723140)	27.98(21.11–36.61)	272,216(213938–342074)	13.58(10.58–17.28)	-2.48(-2.60 to-2.36)
Low-middle SDI^e^	389,698(283359–525579)	24.75(18.26–32.88)	267,151(204075–350300)	13.64(10.44–17.84)	-1.92(-2.03 to-1.81)
Low SDI^e^	170,045(121225–232190)	21.62(15.86–29.14)	211,907(161601–276408)	13.98(10.85-18.00)	-1.39(-1.54 to-1.24)
Region					
East Asia	361,994(264712–484254)	29.98(21.80-40.12)	119,068(87368–155244)	11.98(8.80-15.87)	-3.26(-3.53 to-2.98)
Southeast Asia	92,967(69130–126786)	16.94(12.75–22.74)	52,065(40179–67535)	8.56(6.52–11.25)	-2.20(-2.36 to-2.03)
Oceania	970(694–1349)	11.15(8.14–15.06)	1497(1107–1966)	8.87(6.74–11.52)	-0.53(-0.72 to-0.35)
Central Asia	63,416(50423–80620)	73.26(58.83–92.86)	45,598(36603–57511)	47.53(38.06–59.93)	-1.59(-1.81 to-1.38)
Central Europe	83,023(64191–106854)	79.76(61.51–103.90)	30,795(24887–39018)	41.76(32.29–55.63)	-2.05(-2.16 to-1.94)
Eastern Europe	105,379(81258–135062)	55.73(42.54–71.97)	53,714(43935–66193)	37.32(29.80-46.53)	-1.42(-1.80 to-1.04)
High-income Asia Pacific	69,556(56101–86518)	48.48(38.43–61.28)	50,493(41756–59733)	27.84(22.21–35.31)	-1.73(-1.83 to-1.63)
Australasia	12,161(9655–15403)	66.61(52.19–85.61)	12,838(10741–15495)	46.30(37.13–58.75)	-0.78(-0.96 to-0.60)
Western Europe	152,559(118404–196222)	55.28(42.22–72.85)	100,152(78774–128630)	35.51(27.02–46.96)	-1.32(-1.42 to-1.23)
Southern Latin America	40,921(33658–50407)	79.74(65.69–98.04)	21,221(17385–26343)	39.79(32.29–50.14)	-2.07(-2.22 to-1.91)
High-income North America	102,965(83593–128124)	39.84(31.78–50.62)	111,362(94929–132300)	30.94(25.46–37.91)	-0.80(-0.97 to-0.63)
Caribbean	15,080(11657–19581)	37.82(29.31–49.02)	9093(7283–11461)	22.14(17.60–28.00)	-1.70(-1.77 to-1.62)
Andean Latin America	48,664(38042–61325)	97.45(77.31-121.49)	23,920(19903–28547)	37.37(30.93–44.89)	-3.47(-3.63 to-3.31)
Central Latin America	96,981(73677–127278)	44.65(34.28–58.17)	48,233(38246–60770)	21.93(17.32–27.89)	-2.62(-2.86 to-2.37)
Tropical Latin America	46,067(32885–62281)	26.24(18.83–35.36)	23,481(18181–30109)	12.27(9.33–16.01)	-2.43(-2.53 to-2.33)
North Africa and Middle East	113,975(85081–150603)	24.56(18.63–31.89)	94,812(73968–120724)	15.01(11.75–19.14)	-1.68(-1.77 to-1.59)
South Asia	347,708(247091–479301)	24.18(17.56–32.78)	221,894(163652–298668)	12.92(9.62–17.33)	-1.93(-2.09 to-1.77)
Central Sub-Saharan Africa	25,578(18374–35515)	27.68(20.14–38.03)	32,274(25071–42254)	16.50(13.03–21.26)	-1.83(-2.11 to-1.54)
Eastern Sub-Saharan Africa	57,580(40329–81213)	17.94(12.70-24.82)	63,461(46390–88676)	10.54(7.80-14.58)	-1.66(-1.86 to-1.45)
Southern Sub-Saharan Africa	12,498(9326–16614)	18.18(13.73–24.08)	9973(7802–12821)	12.09(9.50-15.59)	-1.37(-1.63 to-1.11)
Western Sub-Saharan Africa	70,773(51812–95247)	23.19(17.28–30.80)	117,495(91325–151795)	16.88(13.33–21.34)	-0.99(-1.10 to-0.89)

^a^ Age-standardized incidence rate, ^b^ Estimated annual percentage change, ^c^ Uncertainty interval, ^d^ Confidence interval, ^e^ Socio-demographic index

**Fig. 1:**
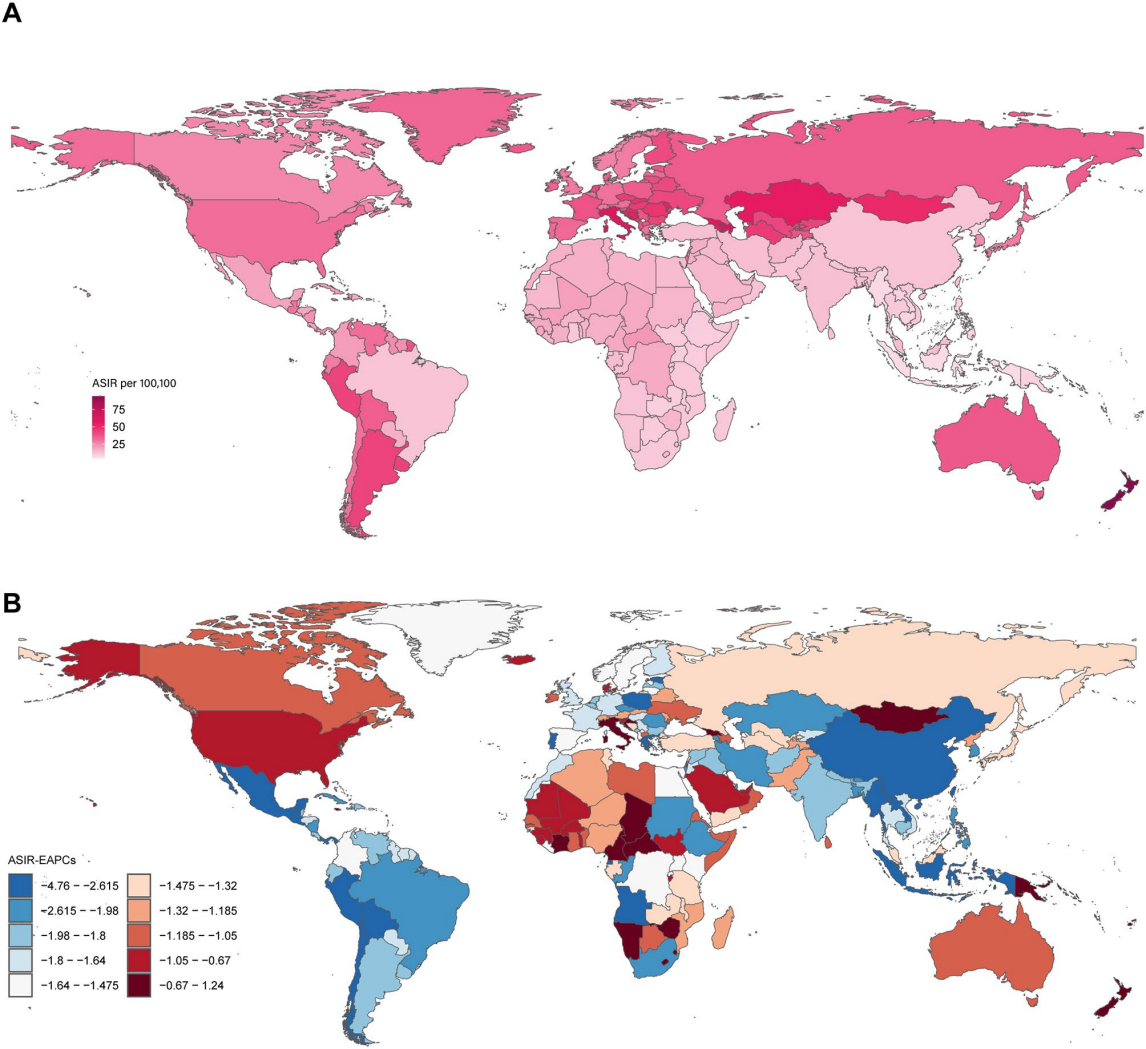
The world map of foreign body aspiration. (**A**) Age-standardized incidence rate (ASIR) of foreign body aspiration in 2021, by country. (**B**) Estimated annual percentage change of ASIR from 1990 to 2021, by country

Regionally, South Asia recorded the highest number of incident cases in 2021 (221,894; 95% UI: 163,652–298,668), followed by East Asia and Western Sub-Saharan Africa. Central Asia had the highest ASIR (47.53 per 100,000; 95% UI: 38.06–59.93), while Southeast Asia recorded the lowest (8.56 per 100,000; 95% UI: 6.52–11.25) (Table 1). Among 204 countries, India, China, the United States, and Nigeria collectively accounted for 35.3% of global cases. The highest ASIRs were reported in New Zealand (93.98 per 100,000; 95% UI: 77.59–112.69), Azerbaijan (66.43 per 100,000; 95% UI: 53.32–81.30), and Italy (60.71 per 100,000; 95% UI: 47.44–78.32) (Fig. 1A). From 1990 to 2021, Although most countries experienced decreasing ASIRs, notable increases were observed in Italy (EAPC: 1.24; 95% CI: 0.75–1.72), Georgia (EAPC: 0.24; 95% CI: 0.07–0.42), and Zimbabwe (EAPC: 0.19; 95% CI: 0.00–0.38) (S2 Table and Fig. 1B).

### Global and regional mortality

Globally, deaths due to FBA increased slightly, from 99,329 (95% UI: 80,764–112,381) in 1990 to 103,915 (95% UI: 82,081–113,555) in 2021. However, the age-standardized death rate (ASDR) decreased from 1.95 per 100,000 (95% UI: 1.62–2.15) in 1990 to 1.37 per 100,000 (95% UI: 1.07–1.51) in 2021, with an EAPC of -1.31 (95% CI: -1.42 to -1.20) (Table 2).

**Table 2:** Deaths and ASDR ^a^ of foreign body aspiration in 1990 and 2021, and Temporal trends

	1990		2021		1990–2021 EAPC ^b^(95%CI^d^)
	Death cases (95%UI^c^)	ASDR ^a^ per 100,000 (95% UI^c^)	Death cases (95%UI^c^)	ASDR ^a^ per 100,000 (95% UI^c^)	
Global	99,329(80764–112381)	1.95(1.62–2.15)	103,915(82081–113555)	1.37(1.07–1.51)	-1.31(-1.42 to -1.20)
Sex					
Males	59,248(41650–69292)	2.41(1.77–2.78)	62,346(44656–68511)	1.76(1.26–1.94)	-1.26(-1.39 to-1.13)
Females	40,081(34535–43824)	1.53(1.34–1.66)	41,569(34716–45610)	1.03(0.84–1.14)	-1.35(-1.43 to-1.28)
Socio-demographic index					
High SDI^e^	17,558(16624–18075)	1.91(1.80–1.97)	29,539(25758–31634)	1.63(1.48–1.73)	-0.51(-0.60 to -0.41)
High-middle SDI^e^	21,771(20112–25962)	2.29(2.11–2.75)	21,623(18975–23396)	1.50(1.30–1.63)	-1.94(-2.23 to -1.66)
Middle SDI^e^	36,047(28215–44452)	2.14(1.68–2.57)	27,402(19527–30872)	1.31(0.94–1.48)	-1.75(-1.84 to -1.67)
Low-middle SDI^e^	15,230(10023–17596)	1.19(0.78–1.35)	14,169(9588–16462)	0.91(0.62–1.03)	-0.83(-0.93 to -0.73)
Low SDI^e^	8600(4907–11163)	1.25(0.76–1.56)	11,074(5455–14628)	1.00(0.55–1.31)	-0.66(-0.76 to -0.55)
Region					
East Asia	23,538(18981–36972)	2.16(1.76–3.39)	13,798(8151–16605)	1.22(0.76–1.47)	-2.32(-2.54 to -2.11)
Southeast Asia	6059(3639–7144)	1.51(0.89–1.74)	6947(4451–8164)	1.30(0.85–1.52)	-0.50(-0.55 to -0.46)
Oceania	120(69–170)	1.83(1.03–2.53)	269(143–360)	1.96(1.03–2.57)	0.32(0.15 to 0.48)
Central Asia	2161(1907–2443)	2.79(2.49–3.12)	1862(16134 − 2150)	1.95(1.69–2.24)	-1.69(-1.98 to -1.40)
Central Europe	2945(2817–3053)	2.52(2.42–2.63)	1993(1829–2163)	1.32(1.21–1.44)	-1.95(-2.25 to -1.63)
Eastern Europe	6881(6686–7259)	3.04(2.94–3.20)	8391(7777–8975)	3.22(3.00-3.42)	-1.09(-1.85 to -0.32)
High-income Asia Pacific	4198(3670–4421)	2.61(2.27–2.78)	10,126(8335–11222)	1.94(1.70–2.10)	-1.44(-1.72 to -1.17)
Australasia	184(176–192)	0.95(0.90–0.99)	299(270–317)	0.75(0.69–0.80)	-0.13(-0.56 to 0.30)
Western Europe	7866(7458–8139)	1.76(1.69–1.82)	11,807(10184–12686)	1.26(1.14–1.33)	-0.95(-1.11 to -0.80)
Southern Latin America	3389(3282–3505)	6.98(6.76–7.22)	1226(1151–1291)	1.70(1.59–1.81)	-4.48(-5.30 to -3.65)
High-income North America	4392(4162–4519)	1.44(1.37–1.48)	9001(8077–9495)	1.87(1.72–1.99)	1.23(1.05 to 1.42)
Caribbean	1396(999–1663)	3.99(2.98–4.66)	1214(890–1481)	2.63(1.86–3.26)	-1.24(-1.74 to -0.73)
Andean Latin America	6278(4444–7270)	14.46(10.03–16.50)	3474(2691–4738)	5.57(4.31–7.63)	-3.37(-3.79 to -2.95)
Central Latin America	6505(6067–6878)	3.80(3.56–3.98)	5248(4543–6135)	2.22(1.90–2.61)	-1.80(-2.09 to -1.52)
Tropical Latin America	2253(2098–2409)	1.63(1.53–1.73)	4777(4374–5087)	2.12(1.94–2.27)	0.78(0.48 to 1.08)
North Africa and Middle East	5745(3059–7153)	1.45(0.76–1.76)	3524(2079–4301)	0.64(0.38–0.77)	-2.60(-2.66 to -2.55)
South Asia	6549(2739–7968)	0.65(0.28–0.75)	6578(3335–7733)	0.51(0.27–0.60)	-0.71(-0.85 to -0.58)
Central Sub-Saharan Africa	1747(843–2400)	2.05(1.02–2.74)	1662(775–2303)	1.17(0.60–1.66)	-1.74(-1.85 to -1.62)
Eastern Sub-Saharan Africa	3241(1990–4225)	1.23(0.82–1.56)	2819(1699–4734)	0.76(0.52–1.13)	-1.58(-1.64 to -1.53)
Southern Sub-Saharan Africa	955(563–1104)	1.87(1.07–2.21)	1315(772–1628)	1.75(1.03–2.15)	-0.29(-0.56 to -0.01)
Western Sub-Saharan Africa	2925(1788–3983)	1.11(0.73–1.46)	7585(3186–10209)	1.55(0.70–2.03)	0.94(0.57 to 1.31)

^a^ Age-standardized incidence rate, ^b^ Estimated annual percentage change, ^c^ Uncertainty interval, ^d^ Confidence interval, ^e^ Socio-demographic index

In 2021, East Asia, Western Europe, and High-income Asia Pacific recorded the highest number of deaths. The highest ASDRs were observed in Andean Latin America, Eastern Europe, and the Caribbean. While most regions showed decreasing ASDRs from 1990 to 2021, exceptions included High-income North America (EAPC: 1.23; 95% CI: 1.05–1.42), Western Sub-Saharan Africa (EAPC: 0.94; 95% CI: 0.57–1.31), Tropical Latin America (EAPC: 0.78; 95% CI: 0.48–1.08), and Oceania (EAPC: 0.32; 95% CI: 0.15–0.48) (Table 2).

At the national level, China, Japan, the United States, and the Russian Federation accounted for 35.5% of global deaths in 2021. Honduras (6.63 per 100,000; 95% UI: 3.48–8.73), Peru (6.18 per 100,000; 95% UI: 4.45–8.71), and Bolivia (6.06 per 100,000; 95% UI: 4.52–9.69) reported the highest ASDRs. Cabo Verde showed the largest increase in ASDR (EAPC: 9.57; 95% CI: 6.04–13.21), while Chile exhibited the steepest decline (EAPC: -6.46; 95% CI: -7.99 to -4.90) (S3 Table and S1 Fig).

### Global and regional YLLs, YLDs and dalys

In 2021, global estimates for DALYs, YLLs, and YLDs due to FBA were 4,576,487 (95% UI: 3,401,165–5,247,680), 4,481,115 (95% UI: 3,288,800–5,157,242), and 95,372 (95% UI: 67,473–127,076), respectively. All indicators showed declining trends since 1990, with EAPCs of -2.10 (95% CI: -2.19 to -2.02) for DALYs, -2.09 (95% CI: -2.18 to -2.00) for YLLs, and − 2.58 (95% CI: -2.67 to -2.48) for YLDs (Table 3, S4 and S5 Table).

**Table 3:** DALYs ^a^ and agestandardized dalys ^a^ rate for foreign body aspiration in 1990 and 2021, and Temporal trends

	1990		2021		1990–2021 EAPC ^b^(95%CI^d^)
	DALYs ^a^ number (95%UI^c^)	Age-standardized DALYs ^a^ rate per 100,000 (95% UI^c^)	DALYs ^a^ number (95%UI^c^)	Age-standardized DALY^a^ rate per 100,000 (95% UI^c^)	
Global	6955260.86(5493412.85-8050430.75)	119.06(94.91–137.10)	4576486.84(3401165.08-5247679.69)	64.12(47.18–74.24)	-2.10(-2.19 to-2.02)
Sex					
Males	4079926.34(2727337.42-4916711.45)	139.04(95.08–165.60)	2812870.92(1850796.27-3231244.61)	77.73(50.52–90.01)	-2.03(-2.12 to-1.93)
Females	2875334.52(2410978.37-3188174.84)	99.11(83.63-109.52)	1763615.92(1313384.46-2005949.30)	50.85(37.42–58.72)	-2.19(-2.27 to-2.12)
Socio-demographic index					
High SDI^e^	707455.46(672548.58 -729338.90)	90.06(85.36–93.44)	705019.22(656206.71 -741336.32)	64.86(60.26–69.10)	-0.86(-0.93 to-0.78)
High-middle SDI^e^	1434178.02(1305487.18-1765360.68)	150.86(136.82-186.97)	797170.67(696992.00-859181.52)	72.16(61.42–79.51)	-2.95(-3.20,-2.70)
Middle SDI^e^	2872794.50(2278206.83-3594094.04)	150.37(119.15-186.99)	1355894.74(980471.54-1549346.23)	69.43(50.76–80.33)	-2.68(-2.79 to-2.57)
Low-middle SDI^e^	1212449.20(810713.32-1420676.20)	77.31(51.61–89.70)	853942.67(572595.85-1012615.13)	46.76(31.45–55.24)	-1.50(-1.57 to-1.42)
Low SD I^e^	720340.39(413911.20-937352.63)	85.14(50.00-109.86)	859193.12(421740.64-1152521.44)	59.15(29.96–77.84)	-1.09(-1.19 to-0.99)
Region					
East Asia	1980271.34(1584589.21-3074938.23)	173.14(138.60-268.76)	646746.18(404469.55-777930.69)	77.19(50.65–94.11)	-3.24(-3.51 to-2.97)
Southeast Asia	448363.39(273564.38-536725.92)	84.72(52.21-100.17)	318895.93(204405.82-385076.76)	54.40(34.66–65.96)	-1.47(-1.52 to-1.42)
Oceania	8978.37(5368.92-12998.79)	106.71(62.06-150.19)	19573.50(10272.93-26785.96)	115.72(61.82-155.15)	0.41(0.17 to0.64)
Central Asia	166505.34(145067.23-189674.95)	197.65(174.18-223.56)	121860.11(103973.43-143101.53)	123.74(105.65-145.22)	-2.03(-2.29 to-1.76)
Central Europe	175495.09(167790.67-183189.22)	164.00(156.57–172.20)	76726.91(70362.86-83487.18)	64.49(58.58–70.86)	-2.81(-3.12 to-2.51)
Eastern Europe	369623.29(356513.20-389955.78)	179.45(172.09-189.53)	326254.44(302064.49-347313.15)	153.18(143.22-161.58)	-1.67(-2.39 to-0.95)
High-income Asia Pacific	158910.40(130336.85-171674.92)	115.98(94.34-127.98)	162880.68(142646.15-175320.00)	55.73(51.57–62.70)	-2.59(-2.72 to-2.46)
Australasia	9789.85(9337.00-10226.85)	54.49(51.79–57.20)	10304.62(9592.35-10961.73)	38.00(34.75–41.27)	-0.46(-0.88 to-0.03)
Western Europe	272678.02(266116.42-279280.33)	80.53(78.69–82.65)	228125.77(208973.01-239275.38)	39.88(37.42–41.95)	-2.05(-2.25 to-1.84)
Southern Latin America	222019.53(212963.45-231428.40)	443.17(425.21–461.80)	50172.04(47044.83-53312.15)	82.89(75.87–90.50)	-5.35(-6.08 to-4.61)
High-income North America	183836.60(180094.87-187409.90)	69.69(68.42–71.07)	286296.68(268064.28-303527.17)	91.56(84.30–99.40)	1.45(1.26 to1.64)
Caribbean	98058.97(65645.62-120690.11)	248.50(169.81-302.98)	65190.06(41921.54-84075.63)	155.24(96.93-202.48)	-1.29(-1.72 to-0.85)
Andean Latin America	494022.88(353921.20-579987.90)	1001.83(714.11-1165.44)	204796.65(158712.45-269358.73)	324.87(250.51-427.89)	-3.87(-4.24 to-3.49)
Central Latin America	485823.17(453273.78-517942.72)	240.94(225.35-254.91)	295399.12(250396.85-354981.31)	129.99(108.66-158.47)	-1.96(-2.29 to-1.63)
Tropical Latin America	169730.45(156417.00-183716.68)	107.92(99.70-116.54)	189088.72(171317.67-207826.41)	92.38(82.27–103.30)	-0.44(-0.63 to-0.24)
North Africa and Middle East	462623.20(250164.08-582961.88)	100.71(54.51-124.98)	231468.01(138612.94-285865.47)	38.51(22.99–47.51)	-2.98(-3.03 to-2.93)
South Asia	519351.93(236029.68-640341.82)	37.53(17.40-45.27)	332272.54(169746.60-429651.20)	21.95(11.23–28.28)	-1.59(-1.68 to-1.49)
Central Sub-Saharan Africa	148826.62(67474.02-205020.66)	150.83(74.49-204.54)	130388.69(61429.71-181006.78)	73.15(35.38-100.76)	-2.18(-2.35 to-2.02)
Eastern Sub-Saharan Africa	272986.18(166425.84-355831.79)	80.34(50.52–104.00)	217224.87(125635.50-377536.05)	39.98(24.56–66.59)	-2.18(-2.24 to-2.12)
Southern Sub-Saharan Africa	65517.61(39408.27-76009.04)	110.54(65.99-127.55)	78405.73(47292.62-97330.08)	96.58(58.17-119.34)	-0.44(-0.68 to-0.20)
Western Sub-Saharan Africa	241848.61(146687.58-336003.46)	75.47(46.98-102.16)	584415.58(243796.44-794026.18)	87.44(37.82–117.60)	0.45(0.12 to0.78)

^a^ Disability-adjusted life years, ^b^ EAPC Estimated annual percentage change, ^c^ UI Uncertainty interval, ^d^ confidence interval, ^e^ Sociodemographic indices

Regionally, Andean Latin America, the Caribbean, and Eastern Europe had the highest ASRs of DALYs and YLLs in 2021. The lowest rates were recorded in South Asia, Australasia, and North Africa and the Middle East. Central Asia, Andean Latin America, and Central Europe reported the highest ASRs of YLDs. Most regions showed declining trends in ASRs of DALYs, with the steepest decline observed in Southern Latin America (EAPC: -5.35; 95% CI: -6.08 to -4.61). Conversely, High-income North America (EAPC: 1.45; 95% CI: 1.26–1.64), Western Sub-Saharan Africa (EAPC: 0.45; 95% CI: 0.12–0.78), and Oceania (EAPC: 0.41; 95% CI: 0.17–0.64) showed increases (Table 3). The ASR of YLLs showed the largest decrease in Southern Latin America (EAPC: -5.38, 95% CI: -6.12 to -4.63) and the largest increase in High-income North America (EAPC: 1.48, 95% CI: 1.29–1.67) (S5 Table). The ASR of YLDs declined across all regions, with Oceania experiencing the steepest reduction (EAPC: -0.51, 95% CI: -0.70 to -0.32) (S6 Table).

Nationally, Bolivia (Plurinational State of), Peru, and Honduras recorded the highest ASRs of DALYs and YLLs in 2021. Between 1990 and 2021, Chile, Uruguay, and Egypt experienced the largest declines in age-standardized DALYs and YLLs rates. In contrast, Cabo Verde, Lesotho, and the Northern Mariana Islands exhibited the greatest increases in EAPC values (S4 and S5 Table, and S1 Fig). For YLDs, Azerbaijan, Georgia, and Mongolia had the highest ASRs in 2021. Most countries demonstrated declining trends in YLDs, except Italy (EAPC: 1.34; 95% CI: 0.86–1.82), Zimbabwe (EAPC: 0.59; 95% CI: 0.31–0.87), and Georgia (EAPC: 0.11; 95% CI: -0.08 to 0.30) (S6 Table).

### Age and sex patterns

**Fig. 2:**
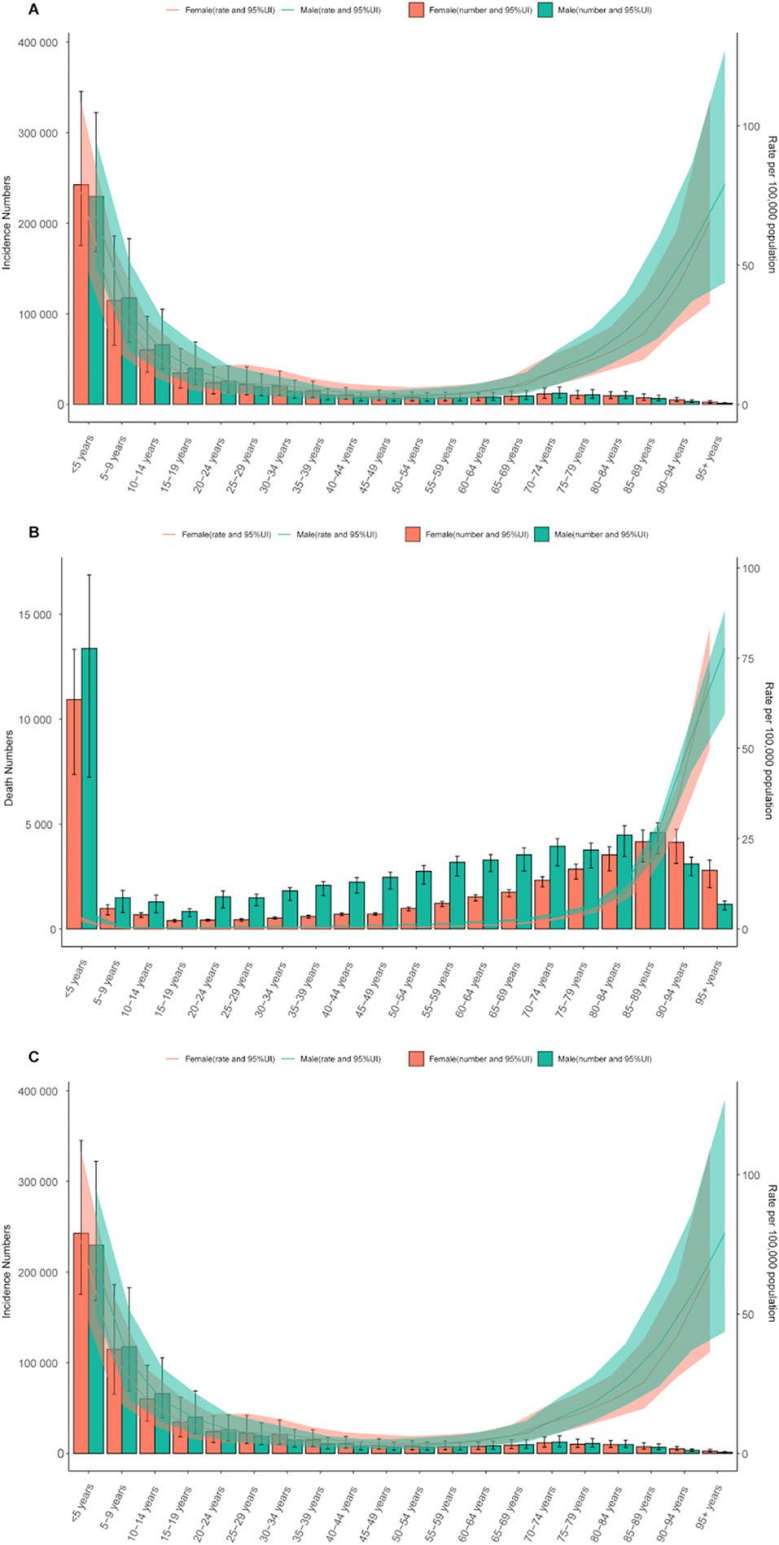
Global burden of foreign body aspiration stratified by age group and sex in 2021. (**A**) Global incidence numbers and age-standardized incidence rate. (**B**) Global death numbers and age-standardized death rate. (**C**) Global disability-adjusted life years (DALYs) numbers and age-standardized DALYs rate

In 2021, the global incidence rate of FBA was highest in children under 5 years, followed by individuals aged 95 years or older (Fig. 2A). Among individuals over 50 years, morbidity rate increased progressively with age in both males and females. Mortality rates for adults aged 14 years and above also rose with advancing age in both genders. Children under 5 years accounted for the highest number of deaths (24,288; 95% UI: 16,219–30,158), while individuals aged ≥ 95 years had the highest mortality rate (72.76 per 100,000; 95% UI: 52.42–84.54) (Fig. 2B). Similarly, the highest DALYs and YLLs rates were observed in individuals aged ≥ 95 years, reflecting a consistent pattern across age groups. The peak YLDs rate also occurred in this age group (Fig. 2C and S2 Fig).

**Fig. 3:**
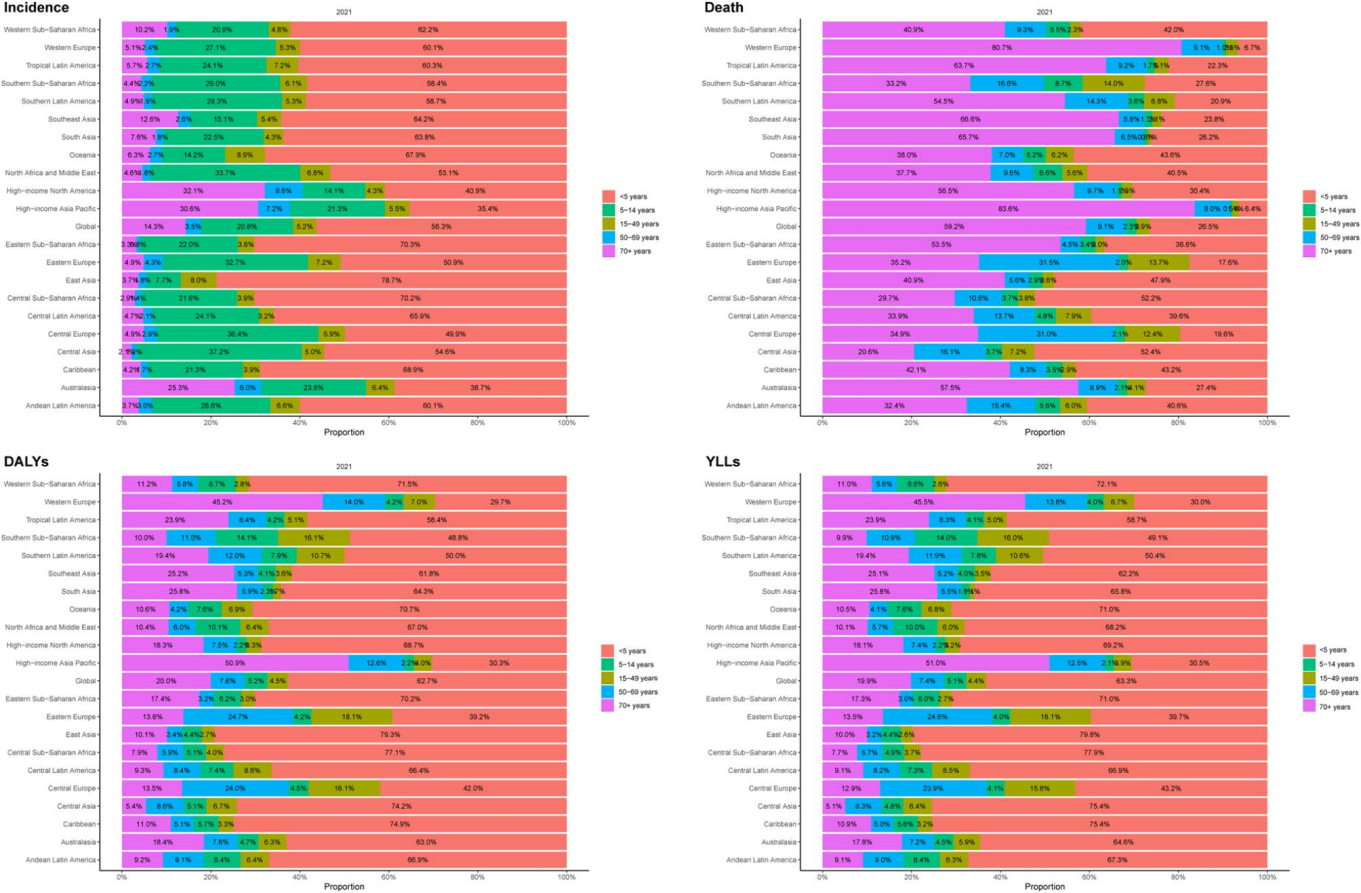
Rate of incidence, deaths, disability-adjusted life years, and years of life lost for foreign body aspiration stratified by age group and region in 2021

Across the 21 GBD regions, children under 5 years exhibited the highest morbidity rates of FBA in 2021 (Fig. 3A). In high-income Asia Pacific and Western Europe, the 70 + age group had the highest mortality rate, while in Central Asia and Central Sub-Saharan Africa, the under-5 age group showed the highest mortality rate (Fig. 3B). In regions outside high-income Asia Pacific and Western Europe, the under-5 age group had the highest DALYs and YLLs rates (Fig. 3C and D).

**Fig. 4:**
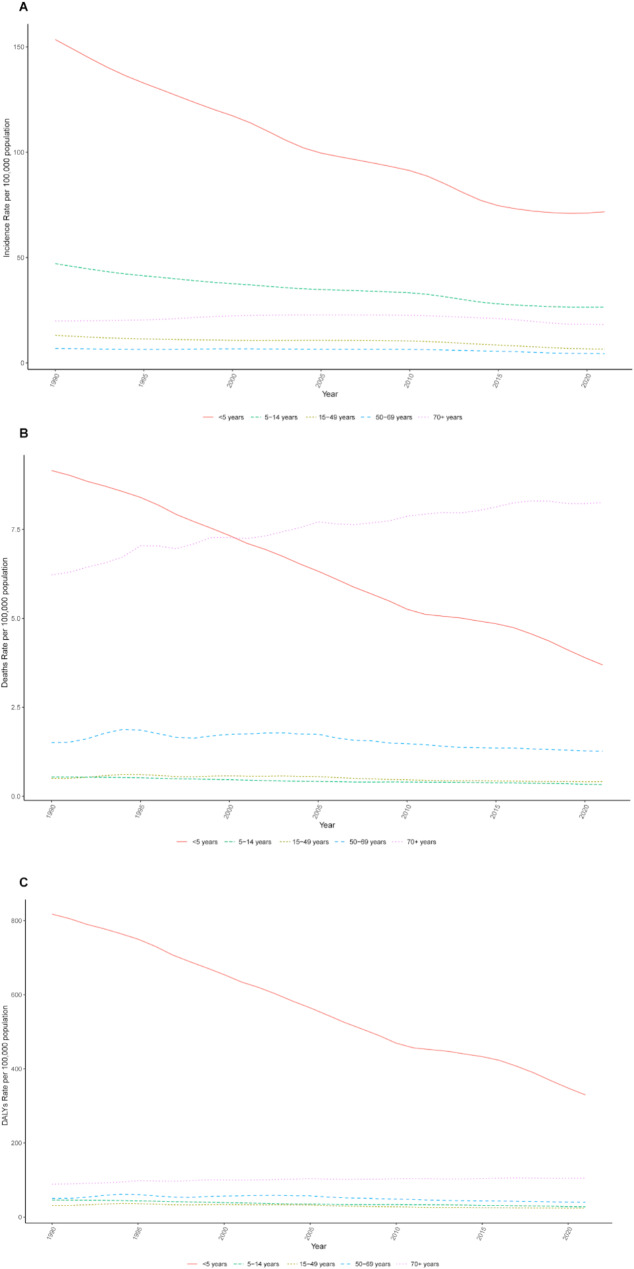
Trend in disease rates for foreign body aspiration. (**A**) Incidence rates stratified by age group from 1990 to 2021. (**B**) Death rates stratified by age group from 1990 to 2021. (**C**) DALYs rates stratified by age group from 1990 to 2021

Temporal trends over the past 32 years revealed that the under-5 age group experienced the largest decreases in incidence, death, and DALYs rates. In contrast, individuals aged 70 years and older showed slight increases in mortality and DALYs rates, despite a decline in morbidity rates (Fig. 4).

Globally in 2021, males exhibited higher mortality, DALYs, and YLLs rates compared to females (Tables 2 and 3 and S5 Table). However, females demonstrated higher incidence and YLDs rates (Table 1 and S6 Table). A gender comparison revealed that females under 10 years of age had higher incidence rates, whereas males had higher rates after the age of 55 (Fig. 2A). Male mortality rates exceeded those of females across all age groups, except for individuals aged ≥ 95 years (Fig. 2B).

### The burden of FBA by SDI

Regions with high SDI recorded the highest morbidity and mortality rates in 2021, with an ASIR of 30.16 per 100,000 (95% UI: 23.87–38.30) and an ASDR of 1.63 per 100,000 (95% UI: 1.48–1.73). The greatest ASRs of DALYs, YLLs, and YLDs were observed in high-middle SDI regions. Middle SDI regions showed the lowest morbidity and YLDs rates, while low-middle SDI regions exhibited the lowest mortality, DALYs, and YLLs rates (Tables 1, 2 and 3 and S5-S6 Table).

**Fig. 5:**
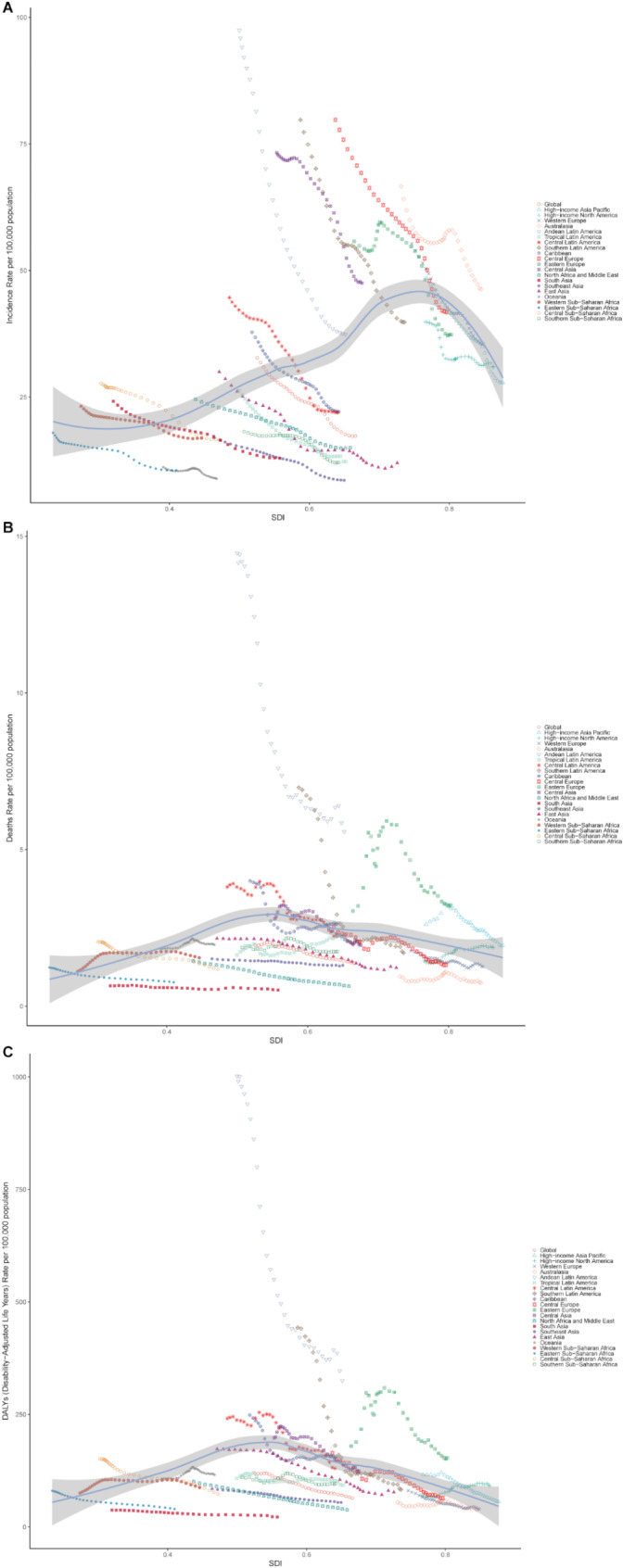
Disease rates for foreign body aspiration by Socio-demographic Index (SDI). Expected values, based on SDI and disease rates in all locations, are shown as a solid line. (**A**) Incidence rates by SDI from 1990 to 2021 globally and in 21 world regions (**B**) Death rates by SDI from 1990 to 2021 globally and in 21 world regions. (**C**) DALYs rates by SDI from 1990 to 2021 globally and in 21 world regions

All five SDI regions showed a consistent decline in ASIR, ASDR, and DALYs rates between 1990 and 2021. Morbidity exhibited a positive correlation with regional SDI up to 0.77, after which ASIR declined with increasing SDI values. For mortality, ASDR initially rose with increasing SDI, peaking at approximately 0.54, before subsequently declining. A similar trend was observed for DALYs and YLLs, which increased with SDI values up to a threshold and then decreased (Fig. 5 and S Fig).

## Discussion

FBA is a life-threatening condition with significant clinical and public health implications, particularly among young children and the elderly [4]. By drawing on data from the GBD 2021, this study provides an extensive global, regional, and national analysis of FBA incidence, mortality, and disease burden trends from 1990 to 2021. It represents the first examination of DALYs, YLLs, and YLDs associated with FBA, thereby offering valuable insights for evidence-based prevention and management strategies.

Our findings show that global FBA incidence declined by 35.3% between 1990 and 2021, with ASIR also showing downward trend. The most pronounced decreases occurred among children under the age of 5, potentially reflecting the impact of improved pediatric safety measures, heightened public awareness, and stricter regulations for food and toy safety [20–22]. Consistent with previous studies, our findings confirm that children under 5 years are at the highest risk for FBA [21, 23]. Several factors contribute to this vulnerability: young children frequently explore their environment by mouthing objects, exhibit active and playful behavior, and are prone to crying or talking while eating. Additionally, they do not have protective laryngeal reflexes completely developed [22]. This is also the reason why current research and prevention strategies disproportionately focus on pediatric cases [24]. Preventive measures for children, such as routine food warning labels and stringent toy safety regulations, have proven effective in reducing FBA incidents [22]. Food Choking Prevention Act in the United State required the Commissioner of Food and Drug to educate parents of young children and to designate week of increased dissemination of choking information to be public. The American Academy of Pediatrics release a policy statement in 2010 with recommendation for government agencies, manufactures, parents, teachers, and health-care professionals to help prevent FBA [22]. Initiatives like the ‘Susy Safe Registry,’ an international surveillance system for foreign body injuries in children aged 0–14 years, play a critical role in identifying risk factors and guiding preventive measures [25]. Despite advancements in safety awareness, FBA remains a significant cause of accidental injuries in children. Previous study [26] reported that some parents are unaware of the risks associated with toy aspiration, even when warning labels are clearly displayed on packaging. Knowledge gaps persist regarding high-risk foods and inappropriate eating behaviors, which may vary across countries due to differences in educational policies. Implementing standardized and comprehensive public education programs is essential to improve awareness of choking hazards and promote safer feeding and play practices.

Although FBA is less prevalent in adults than in children, data from the United States in 2015 indicated that 56% of choking incidents occurred in individuals aged ≥ 74 years [23]. In Japan, individuals aged ≥ 80 years accounted for more than half of all suffocation cases [27]. Our study further supports these findings, demonstrating a high incidence of FBA among adults aged 70 years and older. Notably, in 2021, High-income North America (including the United States) and High-income Asia Pacific (including Japan) reported that this age group accounted for 32.1% and 30.6% of all FBA cases, respectively. This increased risk among the elderly can be attributed to age-related pathological conditions such as dysphagia, poor dental health, neurological disorders, and consuming alcohol and medications such as sedatives, which compromise airway protection and swallowing coordination [28]. However, public awareness regarding knowledge of FBA risks among older individuals remain consistently low [29]. Kramarow, et al. [9] reported that one-quarter of choking incidents occurred in residential institutions, such as nursing homes, rather than in medical or long-term care facilities, where awareness of choking risks is generally higher. They emphasized the need to extend education on FBA risks in the elderly beyond medical institutions to include broader populations. Additionally, targeted prevention strategies for this vulnerable group are essential. These include developing an adult scoring system to assess FBA risk in the elderly, ensuring adequate dentition care and reconstruction from adolescence, and promoting healthy lifestyle habits to mitigate choking risks.

Global FBA-related deaths continued to rise, reaching 103,915 (95% UI: 82,081–113,555) in 2021, particularly in high-risk regions. The ASDR increased in High-income North America, Western Sub-Saharan Africa, Tropical Latin America, and Oceania, accompanied by rising DALYs and YLLs. At the national level, the United States, India, China, and Japan reported the highest numbers of fatalities, collectively accounting for over one-third of the global burden. This trend is likely driven by population aging and a higher proportion of elderly individuals globally, especially in these regions [30]. The elderly patients are at a higher risk of death and poor prognosis [27]. In the United States, the death rate for food-related suffocation is nearly seven times higher among people aged ≥ 65 than among children aged 1–4 [31]. The age- specific analysis in this study also revealed the mortality rate among individuals aged ≥ 70 years was significantly higher than that among those aged < 5 years, with rates of 8.25 per 100,000 and 3.69 per 100,000, respectively, in 2021. Additionally, it was observed that mortality rate among adult patients increased with age, peaking at 95 years and older. From 1990 to 2021, while mortality and DALYs rate have decreased among children aged five and younger, they have increased in individuals aged ≥ 70 years, especially in high-income Asia Pacific and Western Europe. Compared to pediatric cases, elderly patients exhibit distinct risk factors and clinical presentations. Elderly patients often present with multiple comorbidities, which contribute to a poorer prognosis. Non-specific symptoms in this demographic frequently result in missed diagnoses of FBA [24, 32], and neurological impairments often hinder elderly patients from providing a clear history of aspiration [24, 33]. Food particles, rather than metal objects, are the most common foreign bodies in this group, and these are often less visible on imaging modalities like CT scans [34]. All these factors complicate the diagnosis and management of FBA in elderly patients, ultimately leading to serious complications and potential mortality. However, researches involving elderly populations are not as robust as that conducted with children [24]. Investments in advanced diagnostic strategy capable of detecting radiolucent foreign bodies, as well as the development of standardized management protocols, are critical for reducing disease burden in this group.

Moreover, increasing mortality underscores the urgent need for improved treatment strategies for FBA. The huge difference in the value of YLDs and YLLs revealing in this study further highlights this issue. The primary intervention to enhance patient outcomes is the rapid removal of the foreign body, especially in cases involving airway obstruction. However, the approaches for foreign body removal, including prehospital first aid measures (back blows, abdominal thrusts and chest thrusts) [35, 36] and bronchoscopy in hospital settings, have been associated with life-threating complications [2, 37, 38]. Back blows and thrusts may be ineffective in certain situations. For instance, performing these maneuvers can be challenging in patients who are immobilized in wheelchairs, pregnant women, or individuals with morbid obesity. In response to these limitations, suction-based airway clearance devices (ACDs) have been developed and introduced to the market as alternative methods for removing airway obstructions caused by foreign bodies. These devices include non-invasive models such as LifeVac©, LifeVac LLC, Nesconset, New York, NY, USA, and minimally invasive types like DeChoker©, LLC, Wheat Ridge, CO, USA. While existing studies [39] suggest that these devices are effective and safe, both the European Resuscitation Council (ERC) [40]and the International Liaison Committee on Resuscitation (ILCOR) [41] guidelines refrain from making explicit recommendations for or against their use due to a lack of high-quality evidence. The use of ACDs before guideline-recommended first aid maneuvers may delay timely intervention, particularly when employed by untrained individuals. Further high- quality research is necessary to identify optimal first aid techniques tailored to specific choking scenarios, and meticulously design a pre-procedural plan, ensuring both the safety and success of foreign body extraction [42].

Previous studies have consistently reported a higher incidence of FBA in boys compared to girls [43–46], which is often attributed to boys’ more active nature and risk-taking behaviors. However, sex-based analyses in adults reveal significant discrepancies across studies [23, 24, 34, 47, 48], highlighting the need for further investigation. Our study indicates that globally, the incidence rate of FBA is higher in females than in males, while males have higher mortality rates than females across most age groups. This disparity may be influenced by biological, behavioral, and healthcare access factors, though the exact mechanisms remain unclear [49]. Further research is essential to explore the underlying causes of these sex differences, as understanding these dynamics could lead to improved prevention strategies and better clinical outcomes.

This study is the first to explore the relationship between SDI and the global burden of FBA. Our findings reveal that morbidity and mortality rates increase with rising SDI values up to thresholds of 0.77 and 0.54, respectively. Beyond these thresholds, rates decline, suggesting that moderate levels of socioeconomic development are associated with the highest burden of FBA.

Regions with higher SDI levels often report greater morbidity and mortality, which can be attributed to more comprehensive case documentation and access to healthcare services that detect and report FBA cases. Analysis across 21 regions indicates that as SDI rises, signifying improved social and economic development, regional incidence and mortality rates tend to decrease. This trend reflects the impact of preventive measures, advanced medical care, and better public awareness in higher SDI regions.

Despite these benefits, the current high disease burden in high- and middle-high SDI regions is likely driven by population aging. Extended life expectancies, supported by robust healthcare infrastructures, create a demographic profile with a larger proportion of elderly individuals who are particularly susceptible to FBA due to age-related functional decline.

Conversely, low-SDI regions face unique challenges. Inadequate medical resources, limited access to healthcare facilities, and a lack of national surveillance systems contribute to underreporting and misclassification of FBA cases. Many incidents may not result in hospital visits or recorded fatalities, effectively removing them from official statistics. Additionally, insufficient public and healthcare professional awareness of FBA in these regions exacerbates the issue [50]. Given that surveillance plays a crucial role in preventing FBA, establishing a national surveillance registry is urgently needed in low-SDI regions. This registry would enable the identification of high-risk populations, hazardous objects, and common pathogenic pathways, thereby providing essential data to inform targeted and effective preventive interventions.

### Limitations

This study utilized a large, population-based dataset, covering long-term trend analysis from 1990 to 2021; however, several limitations must be acknowledged. First, in regions with limited or scarce data, mathematical modeling was used to estimate incidence and mortality. While these models are robust, they may not fully capture the real-world situation, potentially introducing biases. Second, FBA, as a fatal condition, often results in deaths before diagnosis or outside medical institutions, particularly in low-income regions. This may lead to a significant underestimation of the true disease burden Third, the absence of province-level or urban-rural stratification in GBD 2021 data limits the granularity of our analysis, potentially obscuring important geographic disparities [11, 51]. Fourth, variations in health system infrastructure, particularly emergency medical services, significantly affect FBA prognosis. However, the influence of these systems across different nations was not evaluated in this study. Finally, GBD 2021 does not comprehensively capture non-occupational risk factors for FBA. High-risk populations, such as young children and the elderly, are unlikely to be influenced by occupational risks, leaving gaps in understanding individual-level contributors to FBA burden.

## Conclusion

This study provides a comprehensive analysis of the global, regional, and national burden of foreign body aspiration (FBA) from 1990 to 2021, offering critical insights into its epidemiological trends. While global FBA incidence has generally declined, the number of deaths continues to rise, underscoring the severity of the condition.

Key findings highlight that the greatest burden of disease is observed in high- and middle-high SDI regions, particularly among young children and the elderly. Notably, mortality rates among the elderly are increasing globally, especially in high-income Asia Pacific and Western Europe, reflecting the dual challenges of population aging and region-specific risk factors.

Persistent disparities between regions and nations, alongside variations in affected populations, emphasize the urgent need for tailored public health strategies. Public education activities targeting awareness, prevention, and first-aid training, combined with improvements in emergency medical services and surveillance systems, are critical to reducing FBA morbidity and mortality. Moreover, prevention efforts should consider the unique vulnerabilities of high-risk groups.

## Electronic supplementary material

Below is the link to the electronic supplementary material.

Supplementary Material 1: **Fig. S1** The world map of foreign body aspiration. (A) Age-standardized death rate (ASDR) of foreign body aspiration in 2021, by country. (B) Age-standardized disability-adjusted life years (DALYs) rate of foreign body aspiration in 2021, by country. (C) Estimated annual percentage change of ASDR from 1990 to 2021, by country. (D) Estimated annual percentage change of age-standardized DALYs rate from 1990 to 2021, by country.

Supplementary Material 2: **Table S1** ICD disease codes and clinical descriptions

Supplementary Material 3: **Fig. S2** Global burden of foreign body aspiration stratified by age group and sex in 2021. (A) Global years lived with disability (YLDs) numbers and age-standardized YLDs rate. (B) Global years of life lost (YLLs) numbers and age-standardized YLLs rate.

Supplementary Material 4: **Table S2** Incident cases and ASIR of foreign body aspiration in 1990 and 2021, and temporal trends across 204 countries.

Supplementary Material 5: **Fig.S3** Rate of years lived with disability for foreign body aspiration stratified by age group and region in 2021.

Supplementary Material 6: **Table S3** Deaths and ASDR of foreign body aspiration in 1990 and 2021, and temporal trends across 204 countries.

Supplementary Material 7: **Fig. S4** Trend in disease rates for foreign body aspiration. (A) Years of life lost rates stratified by age group from 1990 to 2021. (B) Years lived with disability rates stratified by age group from 1990 to 2021.

Supplementary Material 8: **Table S4** DALYs and age‑standardized DALYs rate for foreign body aspiration in 1990 and 2021, and temporal trends across 204 countries.

Supplementary Material 9: **Fig. S5** Disease rates for foreign body aspiration by Socio-demographic Index (SDI). Expected values, based on SDI and disease rates in all locations, are shown as a solid line. (A) Years of life lost rates by SDI from 1990 to 2021 globally and in 21 world regions (B) Years lived with disability rates by SDI from 1990 to 2021 globally and in 21 world regions.

Supplementary Material 10: **Table S5** YLLs and age‑standardized YLLs rate for foreign body aspiration in 1990 and 2021, and temporal trends globally, and in 21 regions and 204 countries.

Supplementary Material 11: **Table S6** YLDs and age‑standardized YLDs rate for foreign body aspiration in 1990 and 2021, and temporal trends globally, and in 21 regions and 204 countries.

## Data Availability

No datasets were generated or analysed during the current study.

## Abbreviations

FBA: Foreign body aspiration
GBD: Global Burden of Disease
DALYs: Disability-adjusted life years
YLLs: Years of life lost
YLDs: Years lived with disability
SDI Socio: Demographic Index
ASIR: Age-standardized incidence rate
ASDR: Age-standardized incidence death rate
EAPC: Estimated annual percentage change
ASR: Age-standardized rate
