# Supplementary figures and images for: Global, regional, and national assessment of foreign body aspiration (1990–2021): novel insights into incidence, mortality, and disability-adjusted life years
Source: Scand J Trauma Resusc Emerg Med. 2025 Mar 11;33:40. doi: 10.1186/s13049-025-01352-z (PMC11895196; doi:10.1186/s13049-025-01352-z)

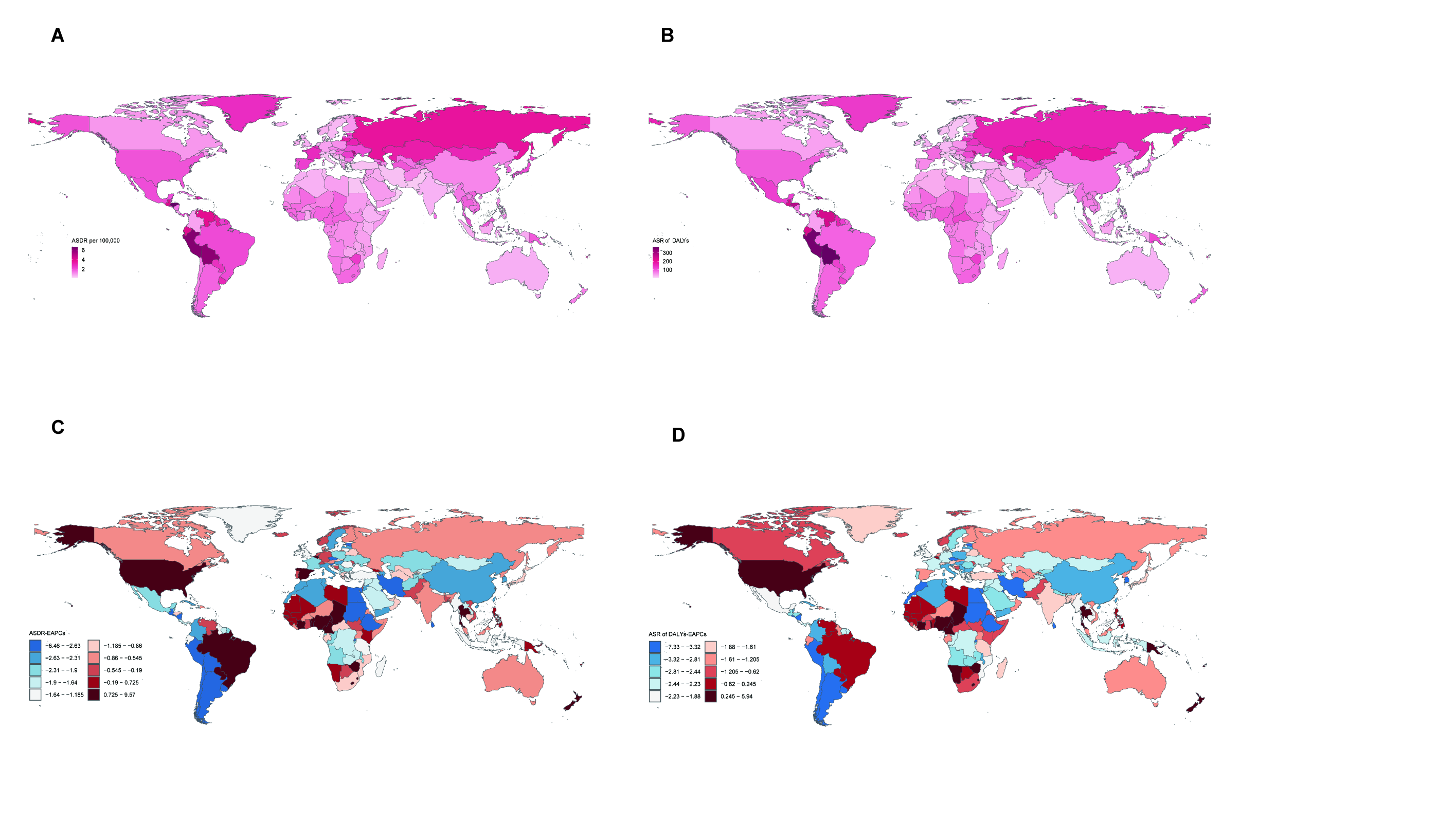

Supplement: Supplementary file 1 — Supplementary Material 1: Fig. S1 The world map of foreign body aspiration. (A) Age-standardized death rate (ASDR) of foreign body aspiration in 2021, by country. (B) Age-standardized disability-adjusted life years (DALYs) rate of foreign body aspiration in 2021, by country. (C) Estimated annual percentage change of ASDR from 1990 to 2021, by country. (D) Estimated annual percentage change of age-standardized DALYs rate from 1990 to 2021, by country. [file 13049_2025_1352_MOESM1_ESM.tif]

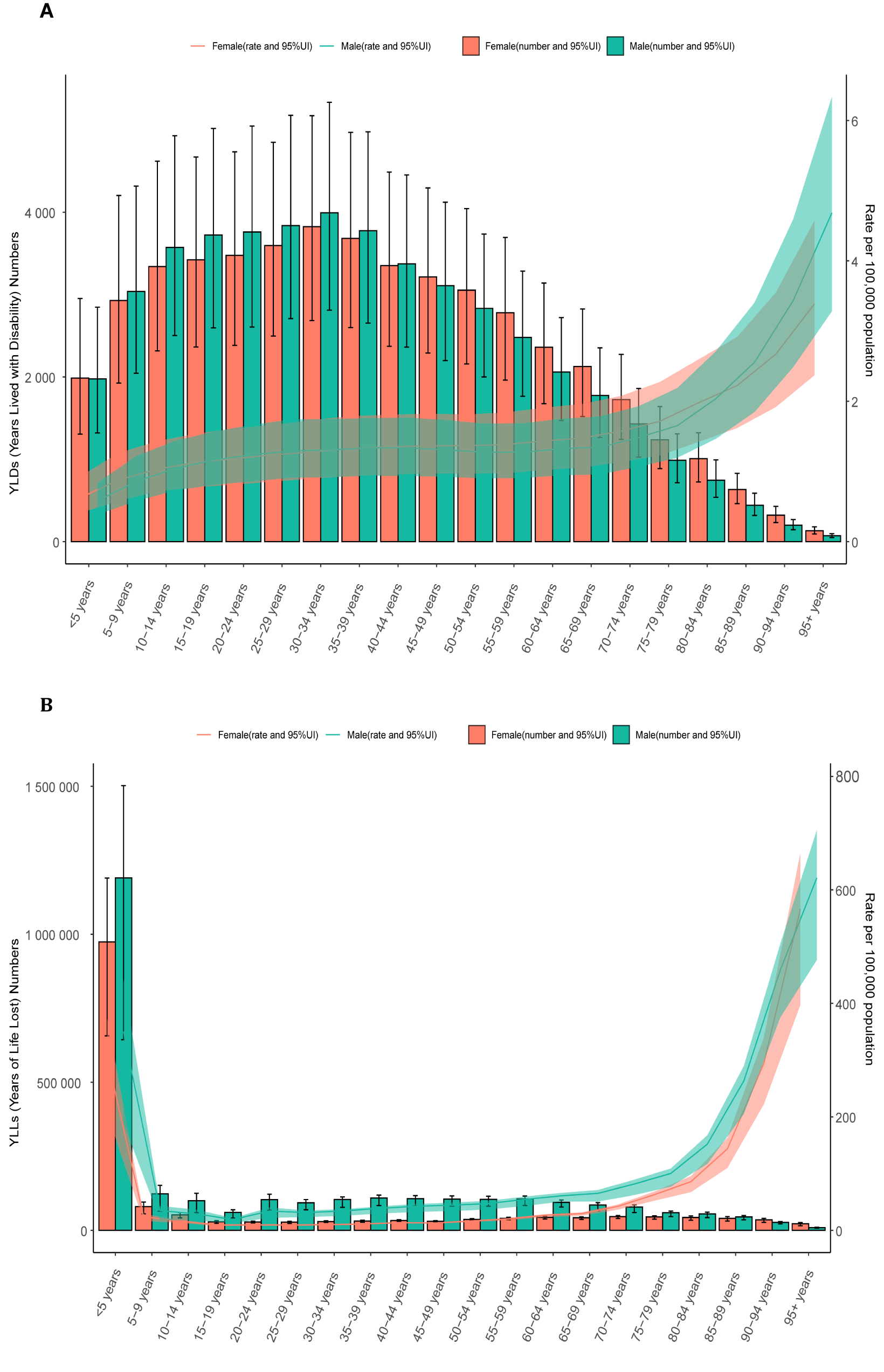

Supplement: Supplementary file 3 — Supplementary Material 3: Fig. S2 Global burden of foreign body aspiration stratified by age group and sex in 2021. (A) Global years lived with disability (YLDs) numbers and age-standardized YLDs rate. (B) Global years of life lost (YLLs) numbers and age-standardized YLLs rate. [file 13049_2025_1352_MOESM3_ESM.tif]

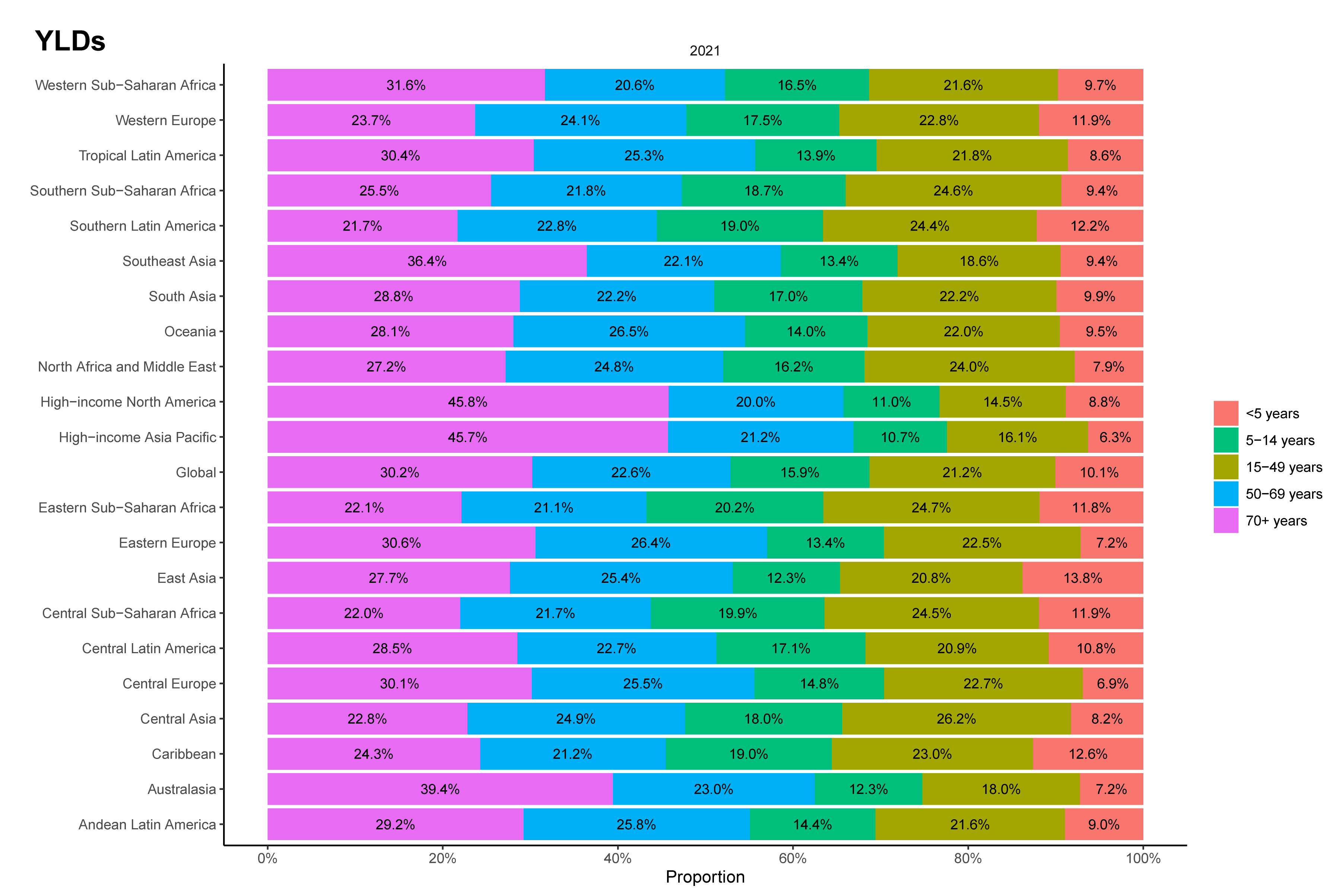

Supplement: Supplementary file 5 — Supplementary Material 5: Fig.S3 Rate of years lived with disability for foreign body aspiration stratified by age group and region in 2021. [file 13049_2025_1352_MOESM5_ESM.tif]

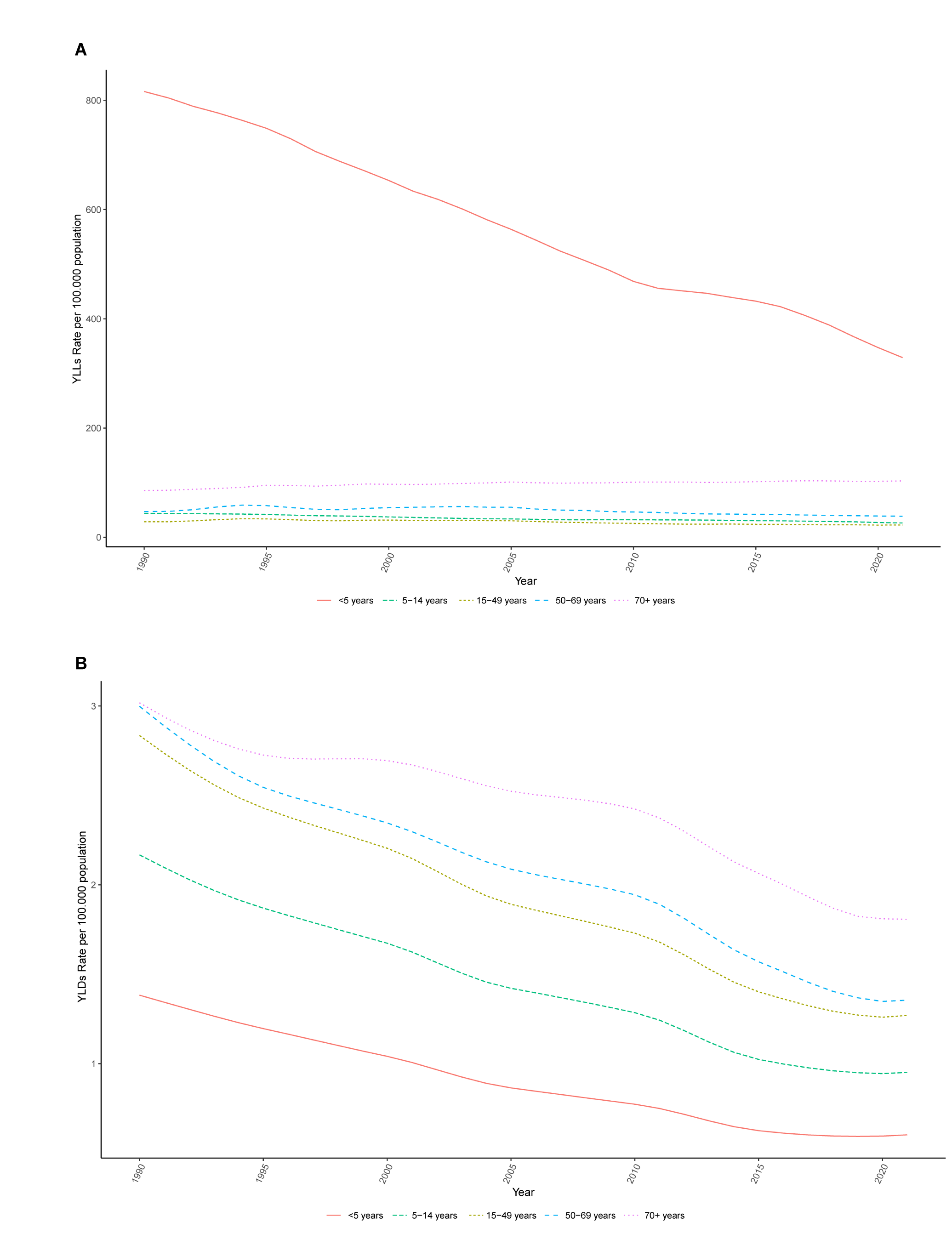

Supplement: Supplementary file 7 — Supplementary Material 7: Fig. S4 Trend in disease rates for foreign body aspiration. (A) Years of life lost rates stratified by age group from 1990 to 2021. (B) Years lived with disability rates stratified by age group from 1990 to 2021. [file 13049_2025_1352_MOESM7_ESM.tif]

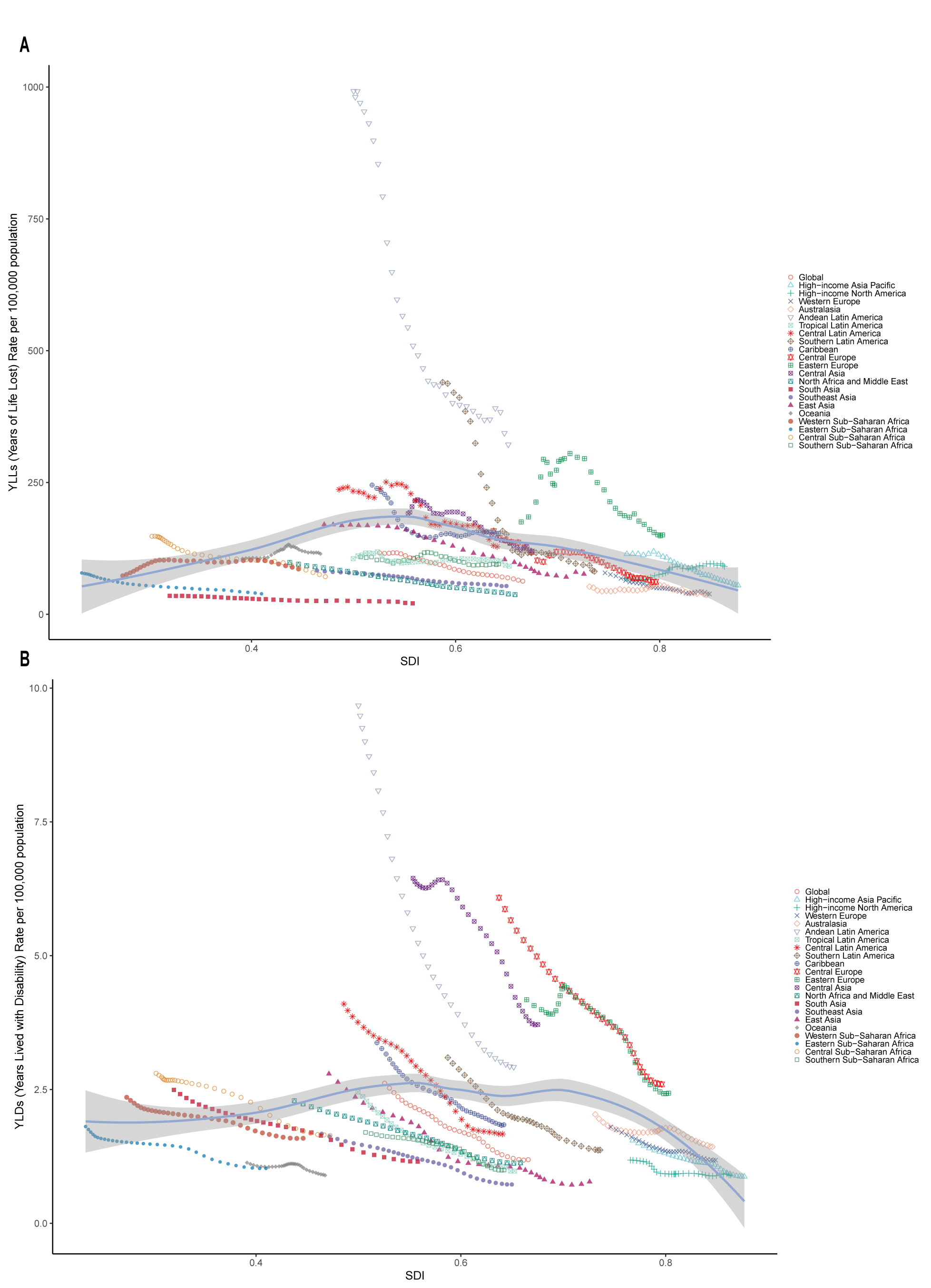

Supplement: Supplementary file 9 — Supplementary Material 9: Fig. S5 Disease rates for foreign body aspiration by Socio-demographic Index (SDI). Expected values, based on SDI and disease rates in all locations, are shown as a solid line. (A) Years of life lost rates by SDI from 1990 to 2021 globally and in 21 world regions (B) Years lived with disability rates by SDI from 1990 to 2021 globally and in 21 world regions. [file 13049_2025_1352_MOESM9_ESM.tif]
