# Supplementary material for: Global, regional, and national assessment of foreign body aspiration (1990–2021): novel insights into incidence, mortality, and disability-adjusted life years
Source: Scand J Trauma Resusc Emerg Med. 2025 Mar 11;33:40. doi: 10.1186/s13049-025-01352-z (PMC11895196; doi:10.1186/s13049-025-01352-z)
Supplement: Supplementary file 2 — Supplementary Material 2: Table S1 ICD disease codes and clinical descriptions [file 13049_2025_1352_MOESM2_ESM.docx]

**S1 Table** ICD disease codes and clinical descriptions

| **Version** | **Code** | **ICD Title** |
| --- | --- | --- |
| **ICD-9** |  |  |
|  | **770.1** | **Massive aspiration syndrome** |
|  | **770.10** | Fetal and newborn aspiration, unspecified |
|  | **770.11** | Meconium aspiration without respiratory symptoms |
|  | **770.12** | Meconium aspiration with respiratory symptoms |
|  | **770.13** | Aspiration of clear amniotic fluid without respiratory symptoms |
|  | **770.14** | Aspiration of clear amniotic fluid with respiratory symptoms |
|  | **770.15** | Aspiration of blood without respiratory symptoms |
|  | **770.16** | Aspiration of blood with respiratory symptoms |
|  | **770.17** | Other fetal and newborn aspiration without respiratory symptoms |
|  | **770.18** | Other fetal and newborn aspiration with respiratory symptoms |
|  | **E911** | **Inhalation and ingestion of food causing obstruction of respiratory tract or suffocation** |
|  |  | Aspiration and inhalation of food [any] (into respiratory tract) NOS^a^ Asphyxia by food [including bone, seed in food, regurgitated food] Choked on food [including bone, seed in food, regurgitated food] Suffocation by food [including bone, seed in food, regurgitated food] Compression of trachea by food lodged in esophagus Interruption of respiration by food lodged in esophagus Obstruction of respiration by food lodged in esophagus Obstruction of pharynx by food (bolus) |
|  | Excludes: | injury, except asphyxia and obstruction of respiratory passage, caused by food (E915) obstruction of esophagus by food without mention of asphyxia or obstruction of respiratory passage (E915) |
|  | **E912** | **Inhalation and ingestion of other object causing obstruction of respiratory tract or suffocation** |
|  |  | Aspiration and inhalation of foreign body except food (into respiratory tract) NOS^a^ Foreign object [bean] [marble] in nose Obstruction of pharynx by foreign body Compression by foreign body in esophagus Interruption of respiration by foreign body in esophagus Obstruction of respiration by foreign body in esophagus |
|  | Excludes: | injury, except asphyxia and obstruction of respiratory passage, caused by foreign body (E915) obstruction of esophagus by foreign body without mention of asphyxia or obstruction in respiratory passage (E915) |
|  | **E913** | **Accidental mechanical suffocation** |
|  | Excludes: | mechanical suffocation from or by: accidental inhalation or ingestion of: food (E911) foreign object (E912) cataclysm (E908-E909) explosion (E921.0-E921.9, E923.0-E923.9) machinery accident (E919.0-E919.9) |
|  | **E913.1** | By plastic bag |
|  | **E913.8** | By other specified means |
|  |  | Accidental hanging, except in bed or cradle |
|  | **E913.9** | By unspecified means |
|  |  | Asphyxia, mechanical NOS^a^ Strangulation NOS^a^ Suffocation NOS^a^ |
| **ICD-10** |  |  |
|  | **W75** | **Accidental suffocation and strangulation in bed** |
|  | **W76** | **Other accidental hanging and strangulation** |
|  | **W78** | **Inhalation of gastric contents** |
|  | **W79** | **Inhalation and ingestion of food causing obstruction of respiratory tract** |
|  | **W80** | **Inhalation and ingestion of other objects causing obstruction of respiratory tract** |
|  | **W83** | **Other specified threats to breathing** |
|  | **W84** | **Unspecified threat to breathing** |

^a^ Not otherwise Specified
