# Supplementary material for: Global, regional, and national assessment of foreign body aspiration (1990–2021): novel insights into incidence, mortality, and disability-adjusted life years
Source: Scand J Trauma Resusc Emerg Med. 2025 Mar 11;33:40. doi: 10.1186/s13049-025-01352-z (PMC11895196; doi:10.1186/s13049-025-01352-z)
Supplement: Supplementary file 4 — Supplementary Material 4: Table S2 Incident cases and ASIR of foreign body aspiration in 1990 and 2021, and temporal trends across 204 countries. [file 13049_2025_1352_MOESM4_ESM.docx]

| **S2 Table** Incident cases and ASIR ^a^ of foreign body aspiration in 1990 and 2021, and temporal trends | | | | | | |
| --- | --- | --- | --- | --- | --- | --- |
|  | **1990** | |  | **2021** | | **1990-2021 EAPC** ^c^**（95%CI**^d^**）** |
|  | **Incident cases (95%UI**^b^**)** | **ASIR**^a^ **per 100,000 (95% UI**^b^**)** |  | **Incident cases (95%UI**^b^**)** | **ASIR**^a^ **per 100,000 (95% UI**^b^**)** |  |
| China | 356486.61(260534.07-476525.75) | 30.55(22.19-40.91) |  | 116833.84(85699.91-152488.61) | 12.15(8.91-16.11) | -3.29(-3.57 to-3.01) |
| Democratic People's Republic of Korea | 2332.03(1676.07-3192.94) | 10.10(7.24-13.74) |  | 1298.75(1027.87-1643.66) | 7.18(5.60-9.23) | -1.21(-1.30 to-1.12) |
| Taiwan (Province of China) | 3174.93(2332.54-4246.91) | 18.43(13.45-25.00) |  | 935.67(701.97-1171.80) | 6.81(4.89-9.00) | -3.04(-3.22 to-2.85) |
| Cambodia | 2161.10(1562.77-3046.91) | 14.26(10.59-19.47) |  | 1393.02(1015.24-1888.03) | 8.12(5.99-10.92) | -1.93(-2.03 to-1.84) |
| Indonesia | 34618.49(24846.69-47856.16) | 15.96(11.55-21.72) |  | 15903.27(11710.24-21515.41) | 6.61(4.78-9.03) | -2.79(-3.08 to-2.50) |
| Lao People's Democratic Republic | 1097.76(804.26-1504.88) | 18.01(13.51-23.98) |  | 668.10(497.51-919.64) | 8.42(6.35-11.46) | -2.78(-2.88 to-2.68) |
| Malaysia | 4775.40(3587.91-6413.57) | 21.81(16.73-28.83) |  | 3706.29(2940.49-4712.48) | 13.56(10.67-17.44) | -1.42(-1.52 to-1.32) |
| Maldives | 78.85(58.40-107.41) | 22.85(17.38-29.58) |  | 38.89(30.39-49.41) | 9.82(7.63-12.47) | -2.92(-3.00 to-2.83) |
| Myanmar | 7934.61(5869.96-10716.97) | 16.76(12.45-22.33) |  | 3949.09(2997.57-5239.79) | 7.42(5.62-9.83) | -3.05(-3.20 to-2.90) |
| Philippines | 12133.94(9101.84-16054.47) | 15.36(11.78-19.80) |  | 9212.59(7162.81-11888.26) | 8.09(6.28-10.36) | -1.98(-2.08 to-1.88) |
| Sri Lanka | 3017.75(2295.92-4010.78) | 16.82(12.92-22.27) |  | 2305.04(1831.15-2908.99) | 12.69(9.93-16.23) | -1.13(-1.64 to-0.62) |
| Thailand | 10010.71(7401.24-14027.75) | 18.65(13.76-25.79) |  | 4981.37(4103.30-6067.92) | 10.93(8.63-14.00) | -1.68(-1.81 to-1.55) |
| Timor-Leste | 177.98(128.93-247.14) | 15.15(11.34-20.61) |  | 134.38(98.23-182.85) | 8.04(5.95-10.65) | -2.31(-2.40 to-2.22) |
| Viet Nam | 16613.22(12111.09-22749.06) | 19.40(14.48-26.04) |  | 9572.48(7363.97-12313.02) | 11.08(8.50-14.19) | -1.79(-1.92 to-1.66) |
| Fiji | 128.42(97.26-171.93) | 14.30(10.96-18.95) |  | 109.46(86.36-136.63) | 11.93(9.43-14.82) | -0.49(-0.62 to-0.36) |
| Kiribati | 7.46(5.08-11.25) | 7.38(5.08-10.97) |  | 6.19(4.22-8.94) | 4.47(3.11-6.40) | -1.27(-1.51 to-1.03) |
| Marshall Islands | 8.03(5.69-11.63) | 12.18(9.04-17.11) |  | 5.26(4.06-6.85) | 9.21(7.19-12.00) | -0.71(-0.93 to-0.49) |
| Micronesia (Federated States of) | 17.53(12.40-25.03) | 12.30(8.95-17.23) |  | 8.18(6.21-10.69) | 8.15(6.20-10.59) | -1.15(-1.30 to-1.00) |
| Papua New Guinea | 584.15(407.55-829.36) | 10.56(7.57-14.39) |  | 1151.64(832.14-1529.61) | 8.82(6.56-11.55) | -0.38(-0.57 to-0.18) |
| Samoa | 28.83(21.04-40.47) | 12.69(9.41-17.35) |  | 20.33(15.19-27.21) | 7.95(6.06-10.34) | -1.32(-1.50 to-1.15) |
| Solomon Islands | 50.35(35.98-71.56) | 10.19(7.47-13.99) |  | 62.35(45.94-84.53) | 7.63(5.68-10.09) | -0.74(-0.96 to-0.52) |
| Tonga | 16.73(12.18-23.38) | 12.72(9.44-17.31) |  | 11.27(8.61-15.06) | 8.93(6.86-11.66) | -0.83(-1.04 to-0.63) |
| Vanuatu | 24.14(17.14-35.93) | 10.65(7.81-15.40) |  | 29.01(21.44-39.42) | 7.60(5.77-10.15) | -0.80(-1.02 to-0.58) |
| Armenia | 3474.18(2783.92-4404.74) | 95.39(76.75-120.59) |  | 1182.08(956.07-1489.03) | 53.95(43.08-68.35) | -2.06(-2.21 to-1.91) |
| Azerbaijan | 7374.50(5922.37-9186.52) | 86.40(69.62-107.45) |  | 5624.19(4537.20-6862.46) | 66.43(53.32-81.30) | -1.06(-1.26 to-0.85) |
| Georgia | 2944.57(2162.79-3977.23) | 60.42(44.23-81.98) |  | 1512.30(1150.25-1963.45) | 56.97(42.92-74.32) | 0.24(0.07 to0.42) |
| Kazakhstan | 17375.82(13904.93-21977.06) | 96.21(77.22-121.70) |  | 10427.69(8481.21-13062.92) | 55.68(45.19-69.88) | -2.38(-2.79 to-1.97) |
| Kyrgyzstan | 4045.26(3164.50-5254.24) | 69.55(54.70-89.91) |  | 3297.44(2558.36-4215.09) | 42.57(33.22-54.12) | -1.79(-2.05 to-1.52) |
| Mongolia | 1873.47(1465.92-2420.54) | 60.93(48.02-78.46) |  | 1866.29(1511.10-2336.55) | 50.45(40.95-63.17) | -0.36(-0.65 to-0.08) |
| Tajikistan | 4414.19(3264.69-5947.80) | 56.47(42.38-75.45) |  | 4554.64(3495.30-6071.64) | 37.72(29.16-50.29) | -1.31(-1.51 to-1.10) |
| Turkmenistan | 3438.26(2678.47-4406.54) | 65.89(51.66-84.00) |  | 2407.78(1905.70-3089.01) | 45.26(35.80-58.13) | -1.35(-1.62 to-1.08) |
| Uzbekistan | 18475.74(14261.98-24255.28) | 62.18(48.63-81.03) |  | 14726.05(11549.49-18995.83) | 41.75(32.58-53.91) | -1.40(-1.56 to-1.25) |
| Albania | 2389.64(1818.82-3122.18) | 62.47(47.65-80.98) |  | 715.58(569.31-900.26) | 39.24(30.39-50.97) | -1.54(-1.65 to-1.42) |
| Bosnia and Herzegovina | 3214.87(2510.71-4110.26) | 79.31(61.21-102.64) |  | 1101.22(873.00-1377.00) | 53.62(41.07-69.06) | -1.32(-1.37 to-1.27) |
| Bulgaria | 4737.24(3792.77-6054.82) | 74.97(59.05-97.81) |  | 1654.21(1315.71-2121.80) | 42.71(32.96-56.67) | -1.97(-2.13 to-1.81) |
| Croatia | 2561.93(1913.87-3424.50) | 69.31(51.46-94.79) |  | 1295.22(1017.47-1713.80) | 51.56(38.80-71.48) | -0.39(-0.63 to-0.16) |
| Czechia | 6895.48(5475.77-8907.38) | 88.25(69.66-116.01) |  | 3089.18(2447.58-4025.45) | 44.55(33.46-60.73) | -1.98(-2.31 to-1.65) |
| Hungary | 5688.09(4445.68-7426.07) | 72.97(55.98-97.85) |  | 2751.75(2173.34-3548.19) | 45.26(34.00-60.98) | -1.64(-1.68 to-1.59) |
| North Macedonia | 1058.47(766.69-1452.58) | 56.68(41.17-78.19) |  | 510.69(386.64-667.71) | 37.75(27.86-50.91) | -1.14(-1.26 to-1.01) |
| Montenegro | 447.46(343.67-584.31) | 79.14(60.44-103.11) |  | 197.95(153.51-258.62) | 46.09(34.98-61.99) | -1.92(-2.13 to-1.70) |
| Poland | 29372.70(22638.28-38080.59) | 89.07(68.26-115.50) |  | 8860.19(7072.26-11231.11) | 35.51(27.12-46.78) | -2.88(-3.00 to-2.76) |
| Romania | 16162.22(12874.57-20640.52) | 82.38(65.07-106.22) |  | 5836.89(4809.68-7239.73) | 47.36(38.05-60.62) | -1.99(-2.09 to-1.88) |
| Serbia | 4957.95(3728.18-6467.00) | 63.68(47.41-84.16) |  | 2107.10(1603.31-2814.60) | 37.77(27.62-53.00) | -1.68(-1.80 to-1.55) |
| Slovakia | 3358.75(2634.73-4398.00) | 71.48(55.80-94.48) |  | 1722.52(1410.04-2137.67) | 48.50(38.20-63.37) | -1.22(-1.36 to-1.07) |
| Slovenia | 850.96(641.57-1168.67) | 55.57(41.21-77.95) |  | 504.58(383.01-673.21) | 39.45(28.51-55.10) | -0.92(-1.11 to-0.72) |
| Belarus | 5304.60(4104.76-6919.40) | 60.78(46.46-79.97) |  | 2625.16(2133.35-3232.61) | 41.17(32.58-52.04) | -1.27(-1.57 to-0.96) |
| Estonia | 1091.40(896.49-1319.80) | 83.40(67.79-102.73) |  | 335.45(268.97-433.86) | 38.52(30.31-51.63) | -2.79(-3.14 to-2.44) |
| Latvia | 1192.98(931.59-1558.37) | 54.87(42.57-73.43) |  | 398.12(313.84-512.21) | 32.31(24.66-43.09) | -1.80(-2.01 to-1.58) |
| Lithuania | 1968.78(1555.34-2512.52) | 63.07(49.53-82.19) |  | 733.19(597.21-901.69) | 40.18(31.91-51.33) | -1.57(-1.88 to-1.26) |
| Republic of Moldova | 3044.95(2516.35-3828.43) | 70.53(58.47-88.57) |  | 1230.87(1034.22-1451.88) | 52.14(42.95-63.20) | -1.25(-1.47 to-1.03) |
| Russian Federation | 68580.81(52677.26-88172.11) | 53.67(40.71-69.67) |  | 37761.89(30934.19-46729.43) | 35.86(28.78-44.91) | -1.47(-1.91 to-1.03) |
| Ukraine | 24195.11(18474.92-31219.01) | 58.18(43.72-76.13) |  | 10628.86(8574.95-13085.11) | 41.49(32.79-52.47) | -1.06(-1.31 to-0.81) |
| Brunei Darussalam | 108.14(80.76-147.48) | 35.84(27.26-47.95) |  | 100.08(79.62-127.93) | 28.53(22.42-37.17) | -0.59(-0.71 to-0.48) |
| Japan | 47156.36(38245.84-58956.95) | 45.91(35.94-58.98) |  | 40773.22(33479.51-48746.78) | 28.82(23.16-36.13) | -1.39(-1.54 to-1.24) |
| Republic of Korea | 21345.94(17276.19-26732.75) | 54.00(43.74-67.55) |  | 8699.78(7066.08-10608.37) | 25.21(19.53-33.71) | -2.46(-2.64 to-2.28) |
| Singapore | 945.80(724.46-1238.94) | 36.61(27.18-49.52) |  | 920.39(712.62-1195.00) | 23.79(17.70-32.12) | -1.29(-1.40 to-1.18) |
| Australia | 7926.00(6253.41-10188.64) | 52.09(40.35-67.80) |  | 8635.52(7134.14-10639.65) | 36.54(28.53-47.57) | -1.07(-1.22 to-0.92) |
| New Zealand | 4235.25(3397.65-5273.12) | 135.94(107.66-172.41) |  | 4202.65(3601.53-4859.63) | 93.98(77.59-112.69) | -0.08(-0.47 to0.32) |
| Andorra | 18.46(14.04-23.82) | 49.77(36.80-66.44) |  | 14.49(11.24-18.27) | 33.20(24.86-43.85) | -1.34(-1.42 to-1.25) |
| Austria | 2613.54(2006.82-3407.59) | 48.34(36.23-64.60) |  | 1750.79(1359.28-2264.68) | 31.38(23.22-42.55) | -1.26(-1.39 to-1.13) |
| Belgium | 3682.54(2873.28-4829.12) | 54.37(41.89-72.79) |  | 2481.30(1987.86-3120.19) | 30.45(23.57-39.72) | -1.80(-2.15 to-1.44) |
| Cyprus | 374.12(280.03-502.19) | 53.84(40.16-72.44) |  | 310.23(238.17-403.89) | 33.24(24.57-44.91) | -1.44(-1.55 to-1.33) |
| Denmark | 1681.25(1286.48-2155.15) | 47.99(35.82-64.01) |  | 1288.90(969.58-1689.41) | 34.38(24.85-46.35) | -0.96(-1.10 to-0.82) |
| Finland | 2323.92(1811.15-2964.45) | 63.10(48.38-82.88) |  | 1397.38(1060.80-1810.29) | 40.60(29.94-54.31) | -1.70(-2.31 to-1.08) |
| France | 29824.35(23330.60-38038.24) | 65.02(50.63-85.45) |  | 19163.01(15248.46-23970.64) | 38.31(29.37-50.68) | -1.68(-1.80 to-1.56) |
| Germany | 28427.89(21790.70-36574.07) | 54.10(40.96-72.28) |  | 16155.91(12460.68-21183.99) | 30.74(22.69-42.18) | -1.69(-1.84 to-1.54) |
| Greece | 4715.26(3663.35-6001.15) | 66.80(51.60-86.12) |  | 1770.03(1360.09-2319.47) | 30.54(22.88-41.59) | -2.68(-2.78 to-2.57) |
| Iceland | 111.91(82.94-149.76) | 48.97(36.08-66.25) |  | 93.11(70.90-124.64) | 36.46(26.72-49.95) | -0.78(-0.94 to-0.62) |
| Ireland | 1629.01(1206.19-2183.97) | 48.90(36.11-65.08) |  | 1217.87(894.42-1607.92) | 33.77(24.44-45.21) | -1.06(-1.20 to-0.92) |
| Israel | 3222.97(2454.35-4284.23) | 62.17(47.63-82.09) |  | 3002.58(2267.79-4014.96) | 32.32(24.31-43.18) | -2.04(-2.20 to-1.88) |
| Italy | 18534.49(13821.34-24583.28) | 50.82(36.64-69.43) |  | 16689.96(13514.02-20910.54) | 60.71(47.44-78.32) | 1.24(0.75 to1.72) |
| Luxembourg | 148.13(116.33-190.81) | 54.95(42.39-73.03) |  | 138.33(106.76-180.30) | 32.11(23.98-43.44) | -1.60(-1.72 to-1.49) |
| Malta | 134.53(99.28-184.16) | 42.66(31.25-58.22) |  | 80.75(62.36-108.41) | 29.12(21.65-40.83) | -1.07(-1.22 to-0.93) |
| Netherlands | 6819.33(5220.64-8722.43) | 61.75(46.28-81.42) |  | 4637.35(3589.81-6073.55) | 41.62(31.00-56.67) | -1.96(-2.30 to-1.62) |
| Norway | 1463.36(1097.79-1892.06) | 45.67(33.69-61.44) |  | 1009.95(770.44-1323.66) | 27.01(20.05-36.90) | -1.55(-1.73 to-1.36) |
| Portugal | 4759.05(3673.52-6095.88) | 68.52(51.92-88.82) |  | 1980.76(1600.72-2478.82) | 32.22(24.71-43.05) | -2.82(-2.94 to-2.70) |
| Spain | 15227.43(11854.55-19739.13) | 54.86(42.14-71.66) |  | 9687.43(7724.90-12280.76) | 32.53(24.47-43.65) | -1.58(-1.72 to-1.45) |
| Sweden | 3067.15(2334.28-3987.01) | 48.76(36.03-65.52) |  | 2026.32(1539.66-2697.53) | 28.11(20.76-38.48) | -1.58(-1.80 to-1.37) |
| Switzerland | 1913.63(1447.50-2479.47) | 39.93(28.98-53.93) |  | 1646.88(1264.20-2161.50) | 29.06(21.36-39.93) | -1.29(-1.44 to-1.14) |
| United Kingdom | 21726.12(16531.53-27821.39) | 50.29(37.62-66.48) |  | 13507.44(10511.22-17330.48) | 29.00(22.18-38.60) | -1.74(-1.88 to-1.59) |
| Argentina | 27927.61(22700.58-34389.88) | 81.11(66.11-99.49) |  | 15982.83(13051.40-19903.99) | 42.97(34.97-53.99) | -1.87(-2.11 to-1.63) |
| Chile | 10790.57(8754.77-13402.64) | 76.79(62.52-95.33) |  | 4119.28(3238.25-5315.48) | 30.70(23.91-40.12) | -2.96(-3.77 to-2.15) |
| Uruguay | 2200.88(1827.97-2725.15) | 76.95(63.80-95.70) |  | 1118.00(918.35-1378.39) | 43.17(35.07-54.26) | -1.94(-2.04 to-1.83) |
| Canada | 9248.44(7653.65-11250.89) | 37.08(29.91-46.42) |  | 9511.26(8127.97-11106.39) | 25.90(21.16-32.53) | -1.12(-1.20 to-1.05) |
| United States of America | 93688.60(75914.03-116687.27) | 40.11(32.02-51.22) |  | 101833.15(86623.96-121469.84) | 31.49(25.92-38.47) | -0.76(-0.95 to-0.57) |
| Antigua and Barbuda | 16.83(12.66-22.59) | 26.82(20.25-35.94) |  | 10.45(8.16-13.11) | 16.23(12.55-20.74) | -1.52(-1.64 to-1.40) |
| Bahamas | 97.07(76.65-122.58) | 36.20(28.76-45.77) |  | 62.99(50.63-78.20) | 22.04(17.72-27.59) | -1.56(-1.68 to-1.44) |
| Barbados | 61.56(45.79-83.34) | 27.85(20.56-38.01) |  | 31.53(24.45-41.68) | 17.23(12.86-23.45) | -1.33(-1.52 to-1.15) |
| Belize | 105.71(83.03-135.58) | 38.99(30.99-49.25) |  | 90.56(73.11-113.42) | 21.72(17.60-26.90) | -1.92(-1.99 to-1.84) |
| Cuba | 3890.09(3042.41-4914.82) | 41.30(32.12-52.66) |  | 1533.61(1222.52-1939.28) | 21.31(16.55-27.81) | -1.98(-2.13 to-1.82) |
| Dominica | 24.86(18.85-32.91) | 29.60(22.43-38.92) |  | 9.14(7.31-11.38) | 18.97(15.07-24.17) | -1.23(-1.35 to-1.11) |
| Dominican Republic | 2860.24(2192.94-3682.12) | 30.85(23.75-39.50) |  | 1748.82(1379.26-2228.92) | 16.62(13.03-21.31) | -1.88(-1.99 to-1.78) |
| Grenada | 39.27(30.11-50.96) | 35.30(27.45-45.19) |  | 17.74(14.27-21.88) | 21.71(17.43-27.43) | -1.51(-1.72 to-1.30) |
| Guyana | 422.74(335.94-532.44) | 41.63(33.42-51.96) |  | 185.71(148.37-228.24) | 24.75(19.74-30.46) | -1.71(-1.76 to-1.65) |
| Haiti | 4068.89(2996.19-5737.36) | 42.85(31.90-59.17) |  | 3774.84(2919.75-4853.29) | 25.09(19.58-32.07) | -1.92(-2.05 to-1.78) |
| Jamaica | 1003.86(761.98-1289.68) | 35.90(27.54-45.80) |  | 570.41(447.21-741.41) | 28.93(22.49-37.85) | -0.39(-0.72 to-0.06) |
| Saint Lucia | 49.93(38.56-65.53) | 29.46(22.99-38.03) |  | 20.32(16.06-25.85) | 17.13(13.34-22.19) | -1.67(-1.75 to-1.58) |
| Saint Vincent and the Grenadines | 41.65(31.43-55.21) | 31.59(24.16-41.37) |  | 18.57(15.14-23.06) | 20.96(16.98-26.44) | -1.29(-1.35 to-1.23) |
| Suriname | 169.99(134.72-213.30) | 38.78(30.90-48.49) |  | 118.17(94.29-148.54) | 23.85(19.15-30.05) | -1.69(-1.76 to-1.63) |
| Trinidad and Tobago | 429.32(330.22-570.31) | 31.65(24.58-41.70) |  | 183.03(148.36-232.14) | 19.16(15.46-24.48) | -1.57(-1.66 to-1.48) |
| Bolivia (Plurinational State of) | 7558.13(5748.48-9950.60) | 82.48(63.84-107.28) |  | 4215.79(3388.93-5152.41) | 34.89(28.10-42.72) | -3.19(-3.32 to-3.06) |
| Ecuador | 6148.86(4872.26-7658.52) | 48.62(38.87-59.89) |  | 4939.18(3952.12-6082.08) | 28.12(22.50-34.66) | -1.82(-1.88 to-1.76) |
| Peru | 34957.22(27276.09-44017.58) | 124.92(97.92-156.54) |  | 14765.01(12215.06-17780.69) | 42.93(35.39-51.72) | -3.92(-4.12 to-3.72) |
| Colombia | 13646.19(10369.57-18316.66) | 34.25(26.21-45.75) |  | 7946.08(6167.56-10431.49) | 21.04(16.13-27.79) | -1.58(-1.83 to-1.32) |
| Costa Rica | 1439.31(1064.66-1997.52) | 37.90(28.40-51.77) |  | 721.39(534.34-994.36) | 19.62(14.39-27.43) | -1.88(-2.03 to-1.73) |
| El Salvador | 4374.63(3399.70-5575.22) | 61.24(47.88-77.66) |  | 1016.83(753.28-1404.04) | 16.05(11.93-22.09) | -4.76(-5.28 to-4.25) |
| Guatemala | 10191.26(7772.22-12935.96) | 76.58(59.54-95.25) |  | 4990.08(4011.07-6190.72) | 30.29(24.48-37.24) | -3.24(-3.46 to-3.02) |
| Honduras | 2407.97(1801.12-3213.14) | 32.46(24.84-42.40) |  | 2099.53(1568.22-2872.24) | 19.10(14.40-26.06) | -1.70(-1.86 to-1.54) |
| Mexico | 46943.75(33728.37-64877.72) | 41.90(30.45-57.42) |  | 21846.10(16752.68-27894.55) | 19.55(14.92-25.16) | -3.07(-3.58 to-2.56) |
| Nicaragua | 2979.60(2292.20-3904.17) | 48.48(38.13-62.45) |  | 1572.93(1217.51-2041.22) | 23.46(18.24-30.47) | -2.51(-2.57 to-2.44) |
| Panama | 1533.30(1248.91-1936.87) | 55.03(45.10-69.05) |  | 882.66(687.54-1175.32) | 22.48(17.60-29.84) | -3.04(-3.13 to-2.95) |
| Venezuela (Bolivarian Republic of) | 13464.57(11056.34-17008.11) | 55.17(45.35-69.44) |  | 7157.46(5846.78-8860.48) | 31.40(25.56-38.97) | -1.84(-2.03 to-1.65) |
| Brazil | 44498.88(31613.76-60336.20) | 26.14(18.69-35.37) |  | 22305.69(17224.11-28574.56) | 12.09(9.16-15.79) | -2.46(-2.57 to-2.36) |
| Paraguay | 1567.86(1213.38-1989.19) | 28.47(22.41-35.70) |  | 1175.54(916.59-1519.62) | 16.84(13.07-21.92) | -1.71(-1.78 to-1.64) |
| Algeria | 8657.62(6442.25-11441.74) | 24.46(18.43-31.92) |  | 7682.28(5937.08-10007.47) | 16.87(13.14-21.85) | -1.29(-1.37 to-1.21) |
| Bahrain | 140.89(103.31-190.45) | 24.41(17.99-33.00) |  | 181.65(138.29-234.92) | 15.62(11.82-20.81) | -1.25(-1.39 to-1.11) |
| Egypt | 16615.59(12574.16-21591.30) | 22.77(17.37-29.17) |  | 17039.48(13035.94-22508.25) | 14.13(10.95-18.39) | -1.62(-1.72 to-1.53) |
| Iran (Islamic Republic of) | 22708.59(16509.17-30398.10) | 27.39(20.30-35.86) |  | 9751.07(7501.85-12448.09) | 13.64(10.42-17.48) | -2.33(-2.44 to-2.23) |
| Iraq | 6718.36(5108.40-8816.40) | 24.71(19.26-31.95) |  | 6806.05(5210.08-8878.89) | 14.67(11.37-18.96) | -1.95(-2.10 to-1.80) |
| Jordan | 1489.06(1112.18-2011.95) | 27.44(20.81-36.60) |  | 2155.18(1635.62-2829.31) | 17.25(13.09-22.66) | -1.64(-1.78 to-1.51) |
| Kuwait | 490.64(366.99-651.24) | 25.36(19.16-33.37) |  | 500.45(377.61-660.34) | 15.08(11.17-20.42) | -1.53(-1.65 to-1.40) |
| Lebanon | 1085.91(799.34-1457.15) | 30.08(22.18-39.95) |  | 914.85(705.26-1192.31) | 19.78(15.11-26.17) | -1.36(-1.48 to-1.24) |
| Libya | 1287.18(937.72-1724.35) | 21.72(15.99-28.58) |  | 871.20(686.94-1101.87) | 15.67(12.13-20.11) | -1.07(-1.12 to-1.02) |
| Morocco | 8846.01(6761.18-11307.39) | 26.74(20.59-33.88) |  | 5776.68(4401.26-7315.85) | 16.69(12.66-21.25) | -1.64(-1.77 to-1.51) |
| Palestine | 715.14(523.90-991.38) | 22.88(17.16-30.81) |  | 1002.71(739.90-1327.94) | 16.18(12.06-21.27) | -1.00(-1.15 to-0.84) |
| Oman | 500.98(345.94-716.26) | 18.10(12.76-25.25) |  | 535.36(389.57-742.04) | 12.33(9.04-17.27) | -1.14(-1.34 to-0.95) |
| Qatar | 112.54(85.68-140.33) | 24.75(18.85-31.20) |  | 270.21(204.61-349.62) | 13.35(9.75-17.99) | -2.13(-2.22 to-2.04) |
| Saudi Arabia | 4305.61(3123.28-5797.73) | 19.71(14.53-26.10) |  | 4555.16(3579.03-5745.50) | 15.40(11.81-19.83) | -0.67(-0.81 to-0.52) |
| Syrian Arab Republic | 5652.75(4277.99-7364.36) | 29.51(22.66-37.92) |  | 2230.58(1725.97-2893.55) | 17.10(13.39-21.85) | -1.97(-2.06 to-1.89) |
| Tunisia | 2637.87(1960.49-3451.23) | 25.48(19.16-32.99) |  | 1577.24(1215.83-2040.93) | 15.92(12.18-20.73) | -1.46(-1.57 to-1.35) |
| Türkiye | 14165.77(10230.95-20147.03) | 20.91(15.35-29.25) |  | 9218.49(7061.52-11972.68) | 13.23(9.96-17.28) | -1.35(-1.46 to-1.24) |
| United Arab Emirates | 467.49(342.72-628.38) | 22.29(16.52-29.85) |  | 830.74(641.13-1093.31) | 16.01(12.15-21.51) | -1.03(-1.15 to-0.91) |
| Yemen | 5526.24(4058.23-7478.89) | 23.95(17.88-31.53) |  | 7147.31(5475.64-9364.93) | 16.07(12.51-20.76) | -1.45(-1.53 to-1.36) |
| Afghanistan | 3617.93(2677.08-4852.30) | 24.93(18.88-32.61) |  | 7154.88(5495.87-9288.44) | 15.55(12.26-19.63) | -1.89(-2.12 to-1.67) |
| Bangladesh | 42414.07(30767.17-58532.45) | 25.53(18.90-34.39) |  | 19491.14(14555.02-26453.19) | 12.63(9.45-17.06) | -2.55(-2.66 to-2.44) |
| Bhutan | 184.70(137.04-244.32) | 21.24(15.93-27.80) |  | 75.89(57.02-102.00) | 11.18(8.37-15.19) | -2.16(-2.30 to-2.02) |
| India | 268246.91(190759.74-369949.14) | 24.77(17.96-33.58) |  | 164599.72(121857.64-220494.59) | 13.19(9.76-17.75) | -1.91(-2.10 to-1.72) |
| Nepal | 6043.37(4358.93-8278.63) | 21.38(15.80-28.69) |  | 3792.91(2835.33-5106.33) | 11.96(8.95-16.16) | -1.93(-2.07 to-1.79) |
| Pakistan | 30818.72(21833.51-42800.15) | 19.19(13.89-26.00) |  | 33934.51(24673.37-46241.57) | 12.00(8.87-16.12) | -1.24(-1.45 to-1.04) |
| Angola | 4899.03(3425.80-6834.86) | 28.26(20.20-38.41) |  | 6278.04(4726.28-8414.96) | 12.38(9.46-16.31) | -3.08(-3.44 to-2.71) |
| Central African Republic | 1185.63(844.88-1646.22) | 26.28(19.07-36.00) |  | 1767.62(1352.70-2357.15) | 22.40(17.36-29.57) | -0.49(-0.54 to-0.44) |
| Congo | 916.47(647.18-1275.37) | 25.01(17.90-34.54) |  | 849.04(641.88-1103.14) | 13.30(10.20-17.08) | -2.26(-2.45 to-2.07) |
| Democratic Republic of the Congo | 18009.01(13076.94-25381.79) | 27.82(20.28-38.89) |  | 22879.03(17821.97-30193.34) | 18.09(14.26-23.43) | -1.48(-1.78 to-1.19) |
| Equatorial Guinea | 234.28(168.92-319.70) | 32.19(23.49-43.49) |  | 200.20(145.63-272.12) | 10.37(7.60-13.99) | -4.12(-4.37 to-3.87) |
| Gabon | 333.74(240.90-454.17) | 23.17(16.82-31.53) |  | 299.89(227.76-390.43) | 14.00(10.72-18.10) | -1.47(-1.66 to-1.28) |
| Burundi | 1494.36(1054.01-2105.19) | 15.71(11.18-21.55) |  | 2244.57(1658.21-3082.25) | 11.29(8.45-15.48) | -1.01(-1.23 to-0.79) |
| Comoros | 128.31(91.10-181.90) | 17.23(12.37-24.17) |  | 97.31(71.86-135.51) | 11.94(8.89-16.62) | -1.05(-1.16 to-0.93) |
| Djibouti | 89.01(62.84-126.22) | 14.82(10.53-20.77) |  | 133.58(97.05-191.32) | 9.43(6.90-13.42) | -1.34(-1.50 to-1.18) |
| Eritrea | 864.63(611.42-1215.57) | 15.49(11.16-21.66) |  | 875.51(631.74-1225.91) | 10.16(7.39-14.16) | -1.14(-1.27 to-1.01) |
| Ethiopia | 18002.89(12458.89-25362.06) | 21.01(14.74-29.33) |  | 15321.26(11046.72-21446.98) | 10.17(7.37-14.17) | -2.29(-2.61 to-1.97) |
| Kenya | 7337.31(4993.90-10538.47) | 19.04(13.30-26.92) |  | 6347.64(4499.33-9005.87) | 10.32(7.41-14.54) | -1.63(-1.85 to-1.40) |
| Madagascar | 3201.61(2253.13-4491.60) | 16.57(11.74-22.96) |  | 4308.77(3106.49-6131.19) | 11.04(8.06-15.52) | -1.26(-1.41 to-1.11) |
| Malawi | 2610.25(1825.52-3747.17) | 15.63(11.05-22.55) |  | 2691.67(1954.35-3730.64) | 10.10(7.38-13.82) | -1.38(-1.54 to-1.22) |
| Mauritius | 200.49(155.00-264.63) | 18.14(14.01-24.09) |  | 119.57(99.23-145.72) | 14.55(11.88-18.13) | -0.71(-1.02 to-0.40) |
| Mozambique | 3758.42(2705.39-5223.51) | 17.18(12.38-23.43) |  | 5482.09(3909.58-7713.20) | 11.43(8.29-15.86) | -1.28(-1.39 to-1.17) |
| Rwanda | 1965.36(1409.01-2774.94) | 16.33(11.75-22.77) |  | 1688.07(1228.06-2358.08) | 10.04(7.38-13.92) | -1.60(-1.81 to-1.40) |
| Seychelles | 11.96(8.97-16.41) | 15.06(11.34-20.32) |  | 8.21(6.38-10.62) | 9.43(7.27-12.28) | -1.39(-1.53 to-1.25) |
| Somalia | 2253.19(1591.78-3223.25) | 16.45(11.51-23.47) |  | 3983.56(2886.25-5593.64) | 11.16(8.14-15.44) | -1.17(-1.32 to-1.01) |
| United Republic of Tanzania | 7517.38(5386.68-10407.80) | 17.41(12.58-23.90) |  | 9351.61(6895.19-12653.96) | 11.26(8.34-15.12) | -1.43(-1.60 to-1.26) |
| Uganda | 4955.60(3530.60-7020.81) | 16.03(11.47-22.22) |  | 6678.55(4853.16-9355.89) | 9.94(7.31-13.81) | -1.52(-1.71 to-1.33) |
| Zambia | 2044.06(1455.21-2850.80) | 15.07(10.73-21.01) |  | 2680.91(1924.60-3831.24) | 9.63(7.05-13.66) | -1.47(-1.66 to-1.29) |
| Botswana | 285.24(210.58-389.79) | 14.94(11.28-20.14) |  | 260.22(199.80-343.67) | 10.67(8.24-14.08) | -1.07(-1.17 to-0.96) |
| Lesotho | 364.27(264.18-499.54) | 16.43(12.25-22.10) |  | 317.77(248.67-407.40) | 14.86(11.75-18.88) | -0.09(-0.22 to0.04) |
| Namibia | 298.46(217.06-429.55) | 15.18(11.31-21.45) |  | 338.70(260.77-446.05) | 12.16(9.42-15.86) | -0.63(-0.76 to-0.51) |
| South Africa | 8932.42(6678.92-11798.65) | 19.43(14.64-25.53) |  | 5843.91(4533.56-7671.12) | 10.97(8.52-14.44) | -2.02(-2.37 to-1.67) |
| Eswatini | 182.82(134.74-252.06) | 14.58(10.83-19.76) |  | 184.11(140.33-236.96) | 13.31(10.28-17.05) | -0.10(-0.32 to0.12) |
| Zimbabwe | 2434.41(1780.44-3330.00) | 15.84(11.79-21.49) |  | 3028.08(2385.23-3889.24) | 15.17(12.17-19.17) | 0.19(0.00 to0.38) |
| Benin | 1854.72(1358.75-2467.26) | 22.56(16.96-29.46) |  | 2999.48(2320.23-3828.12) | 15.70(12.47-19.47) | -1.11(-1.20 to-1.03) |
| Burkina Faso | 3419.63(2485.10-4663.75) | 21.19(15.54-28.51) |  | 5474.70(4245.54-7094.32) | 15.53(12.14-19.66) | -0.90(-1.02 to-0.79) |
| Cameroon | 3589.97(2606.54-4811.35) | 21.45(15.99-28.26) |  | 7847.24(6103.61-9979.19) | 18.46(14.68-22.88) | -0.33(-0.50 to-0.16) |
| Cabo Verde | 189.07(143.03-249.33) | 35.89(27.72-46.44) |  | 109.24(88.86-137.58) | 21.84(17.77-27.59) | -1.72(-1.84 to-1.60) |
| Chad | 2177.09(1561.79-2934.31) | 21.38(15.60-28.23) |  | 5265.82(4093.23-6786.15) | 18.22(14.46-22.89) | -0.42(-0.54 to-0.30) |
| Côte d'Ivoire | 4217.54(3066.32-5695.31) | 21.44(15.89-28.16) |  | 6372.80(4942.81-8055.88) | 17.07(13.43-21.12) | -0.60(-0.72 to-0.49) |
| Gambia | 338.80(252.35-447.20) | 21.57(16.31-28.49) |  | 520.23(399.13-679.35) | 16.71(13.06-21.21) | -0.62(-0.74 to-0.51) |
| Ghana | 3995.21(2832.96-5605.87) | 17.59(12.73-24.37) |  | 4834.78(3558.73-6727.52) | 11.29(8.43-15.55) | -1.15(-1.35 to-0.95) |
| Guinea | 2273.83(1671.79-3015.51) | 23.27(17.55-30.45) |  | 3435.58(2654.52-4335.66) | 18.12(14.35-22.35) | -0.67(-0.77 to-0.56) |
| Guinea-Bissau | 387.74(279.80-518.27) | 23.76(17.65-31.14) |  | 431.41(338.99-556.74) | 15.47(12.44-19.35) | -1.39(-1.49 to-1.28) |
| Liberia | 1058.50(784.21-1423.11) | 26.54(20.06-35.11) |  | 1143.57(898.23-1444.11) | 16.88(13.56-20.87) | -1.61(-1.68 to-1.55) |
| Mali | 3792.02(2835.70-4955.26) | 26.25(19.99-33.61) |  | 7783.32(6013.50-9945.22) | 20.43(16.28-25.51) | -0.75(-0.82 to-0.69) |
| Mauritania | 635.28(462.02-853.90) | 20.48(15.20-27.16) |  | 890.99(691.30-1195.35) | 15.12(11.95-19.86) | -0.81(-1.04 to-0.58) |
| Niger | 3607.62(2634.40-4832.89) | 25.60(19.22-33.53) |  | 7124.73(5483.70-9038.31) | 17.91(14.15-22.15) | -1.20(-1.25 to-1.16) |
| Nigeria | 33413.32(24242.82-45658.88) | 24.36(17.80-33.06) |  | 56164.90(42722.44-73792.56) | 16.95(13.19-21.77) | -1.21(-1.33 to-1.10) |
| Sao Tome and Principe | 61.05(47.56-79.03) | 32.66(25.95-41.72) |  | 57.53(45.47-72.64) | 23.22(18.67-29.12) | -1.30(-1.38 to-1.21) |
| Senegal | 2796.24(2058.79-3722.61) | 22.65(17.07-29.56) |  | 3123.61(2426.51-4017.67) | 15.48(12.15-19.48) | -1.15(-1.29 to-1.01) |
| Sierra Leone | 1646.66(1211.84-2170.58) | 25.09(18.97-32.68) |  | 2241.09(1749.77-2915.69) | 19.15(15.28-24.48) | -0.84(-0.98 to-0.69) |
| Togo | 1316.06(974.51-1746.62) | 22.40(16.92-29.31) |  | 1672.43(1293.95-2136.07) | 16.25(12.88-20.30) | -0.86(-1.00 to-0.72) |
| American Samoa | 9.31(6.95-12.50) | 14.53(10.94-19.06) |  | 5.67(4.50-7.36) | 13.01(10.36-16.71) | -0.08(-0.31 to0.14) |
| Bermuda | 14.39(10.93-18.89) | 31.35(23.37-42.13) |  | 6.39(4.98-8.37) | 18.58(13.99-25.23) | -1.42(-1.66 to-1.18) |
| Cook Islands | 3.24(2.40-4.39) | 14.94(11.14-19.96) |  | 1.35(1.04-1.76) | 9.90(7.47-13.33) | -1.03(-1.21 to-0.84) |
| Greenland | 25.49(21.14-30.83) | 51.66(43.18-61.66) |  | 16.03(13.78-18.73) | 32.94(28.07-39.32) | -1.60(-1.66 to-1.53) |
| Guam | 18.44(13.57-26.03) | 12.08(8.80-17.14) |  | 11.74(8.99-15.52) | 8.73(6.57-11.78) | -0.74(-0.93 to-0.54) |
| Monaco | 7.84(6.08-9.94) | 49.51(36.39-66.91) |  | 7.59(5.96-9.78) | 35.30(26.83-47.41) | -1.06(-1.13 to-0.99) |
| Nauru | 1.16(0.81-1.64) | 8.36(6.04-11.39) |  | 0.85(0.63-1.15) | 6.66(5.01-8.78) | -0.66(-1.07 to-0.26) |
| Niue | 0.30(0.22-0.42) | 11.88(8.91-15.97) |  | 0.11(0.09-0.14) | 8.25(6.48-10.75) | -1.08(-1.25 to-0.90) |
| Northern Mariana Islands | 5.96(4.39-8.35) | 12.80(9.34-18.04) |  | 4.21(3.35-5.20) | 11.00(8.64-13.54) | -0.09(-0.31 to0.13) |
| Palau | 2.21(1.68-2.88) | 14.27(10.87-18.60) |  | 1.28(1.02-1.58) | 10.11(7.95-12.89) | -1.03(-1.12 to-0.94) |
| Puerto Rico | 1222.44(909.28-1677.29) | 36.19(26.83-49.70) |  | 387.50(304.91-496.21) | 20.95(15.68-28.85) | -1.59(-1.71 to-1.48) |
| Saint Kitts and Nevis | 10.90(7.94-14.98) | 22.98(16.88-31.28) |  | 5.30(4.08-6.80) | 13.82(10.57-18.17) | -1.42(-1.56 to-1.27) |
| San Marino | 6.98(5.21-9.12) | 42.86(30.70-58.66) |  | 5.78(4.38-7.50) | 32.07(23.31-43.69) | -0.84(-0.98 to-0.70) |
| Tokelau | 0.29(0.21-0.39) | 15.20(11.50-20.51) |  | 0.12(0.09-0.16) | 9.82(7.36-13.06) | -1.24(-1.40 to-1.07) |
| Tuvalu | 1.49(1.06-2.01) | 11.90(8.63-15.69) |  | 0.92(0.68-1.20) | 7.24(5.41-9.50) | -1.45(-1.60 to-1.31) |
| United States Virgin Islands | 39.22(30.14-51.26) | 35.99(27.60-47.16) |  | 10.54(8.57-13.50) | 19.79(15.24-25.88) | -1.70(-1.83 to-1.57) |
| South Sudan | 1316.41(919.25-1917.67) | 14.39(10.05-20.74) |  | 1520.89(1113.07-2119.23) | 10.56(7.74-14.60) | -0.91(-1.04 to-0.78) |
| Sudan | 8170.05(5977.30-11229.42) | 27.16(20.06-36.85) |  | 8522.32(6632.97-11198.69) | 15.52(12.16-20.07) | -2.08(-2.20 to-1.97) |

^a^ Age-standardized incidence rate, ^b^ Uncertainty interval, ^c^ Estimated annual percentage change, ^d^ Confidence interval,
