# Supplementary material for: Global, regional, and national assessment of foreign body aspiration (1990–2021): novel insights into incidence, mortality, and disability-adjusted life years
Source: Scand J Trauma Resusc Emerg Med. 2025 Mar 11;33:40. doi: 10.1186/s13049-025-01352-z (PMC11895196; doi:10.1186/s13049-025-01352-z)
Supplement: Supplementary file 6 — Supplementary Material 6: Table S3 Deaths and ASDR of foreign body aspiration in 1990 and 2021, and temporal trends across 204 countries. [file 13049_2025_1352_MOESM6_ESM.docx]

| **S3 Table** Deaths and ASDR^a^ of foreign body aspiration in 1990 and 2021, and temporal trends | | | | | | |
| --- | --- | --- | --- | --- | --- | --- |
|  | **1990** | |  | **2021** | | **1990-2021 EAPC**^c^**（95%CI**^d^**）** |
|  | **Death cases (95%UI**^b^**)** | **ASDR**^a^ **per 100,000 (95% UI**^b^**)** |  | **Death cases (95%UI**^b^**)** | **ASDR**^a^ **per 100,000 (95% UI**^b^**)** |  |
| China | 22784.05(18264.59-36062.81) | 2.17(1.75-3.43) |  | 13140.33(7589.32-15947.67) | 1.22(0.74-1.47) | -2.38(-2.60 to-2.15) |
| Democratic People's Republic of Korea | 384.75(254.64-617.70) | 1.70(1.15-2.73) |  | 253.80(171.08-396.36) | 1.17(0.77-1.93) | -0.81(-1.09 to-0.54) |
| Taiwan (Province of China) | 369.61(353.81-387.04) | 2.31(2.20-2.42) |  | 403.67(362.85-434.13) | 1.57(1.41-1.71) | -1.33(-1.67 to-0.99) |
| Cambodia | 216.90(106.10-349.11) | 1.83(1.25-2.74) |  | 182.52(124.03-279.03) | 1.43(1.00-2.09) | -1.04(-1.17 to-0.90) |
| Indonesia | 1779.98(420.60-2319.50) | 1.00(0.25-1.27) |  | 1323.79(395.24-1620.37) | 0.72(0.21-0.87) | -1.24(-1.33 to-1.16) |
| Lao People's Democratic Republic | 113.53(45.07-187.30) | 2.22(1.27-3.46) |  | 83.58(55.82-127.72) | 1.38(0.97-2.10) | -1.64(-1.70 to-1.57) |
| Malaysia | 293.40(168.20-361.75) | 2.23(1.27-2.70) |  | 488.31(348.12-661.63) | 1.96(1.43-2.67) | -0.76(-0.97 to-0.55) |
| Maldives | 10.91(5.32-14.99) | 5.28(3.20-6.63) |  | 7.74(5.51-10.23) | 2.40(1.74-3.12) | -2.53(-2.58 to-2.49) |
| Myanmar | 838.69(383.71-1302.00) | 2.10(1.21-3.10) |  | 679.84(490.21-980.98) | 1.44(1.06-2.08) | -1.50(-1.66 to-1.34) |
| Philippines | 684.89(423.41-816.62) | 1.17(0.72-1.37) |  | 940.28(584.94-1105.36) | 1.06(0.69-1.23) | 0.08(-0.10 to0.27) |
| Sri Lanka | 379.75(201.07-436.67) | 3.13(1.71-3.61) |  | 317.48(202.32-470.20) | 1.48(0.97-2.19) | -3.06(-3.45 to-2.68) |
| Thailand | 448.58(341.48-693.28) | 1.24(0.87-1.73) |  | 1553.69(940.37-2013.97) | 1.81(1.11-2.29) | 1.56(1.09 to2.03) |
| Timor-Leste | 14.67(6.38-23.56) | 1.45(0.94-2.23) |  | 14.15(10.23-21.83) | 1.14(0.82-1.70) | -0.76(-1.03 to-0.50) |
| Viet Nam | 1258.86(580.69-1596.45) | 1.96(1.00-2.39) |  | 1330.86(776.18-1650.69) | 1.76(0.95-2.19) | -0.31(-0.38 to-0.24) |
| Fiji | 18.88(12.92-25.42) | 3.03(1.99-3.96) |  | 25.62(16.82-33.84) | 3.26(2.14-4.23) | 0.21(0.02 to0.40) |
| Kiribati | 0.23(0.14-0.31) | 0.27(0.17-0.34) |  | 0.24(0.15-0.35) | 0.23(0.14-0.32) | -0.39(-0.47 to-0.31) |
| Marshall Islands | 0.64(0.40-0.83) | 1.75(1.05-2.24) |  | 1.02(0.54-1.39) | 2.22(1.16-2.95) | 0.90(0.67 to1.12) |
| Micronesia (Federated States of) | 1.74(1.07-2.44) | 1.91(1.18-2.58) |  | 1.60(0.83-2.17) | 1.89(0.98-2.56) | -0.03(-0.06 to0.00) |
| Papua New Guinea | 76.89(43.14-111.75) | 1.68(0.88-2.41) |  | 204.39(104.52-280.68) | 1.84(0.92-2.58) | 0.43(0.23 to0.63) |
| Samoa | 2.10(1.40-3.05) | 1.40(0.86-1.97) |  | 2.78(1.59-3.75) | 1.54(0.87-2.06) | 0.42(0.38 to0.46) |
| Solomon Islands | 4.82(2.50-6.95) | 1.58(0.73-2.23) |  | 10.06(5.26-14.93) | 1.74(0.88-2.59) | 0.39(0.27 to0.52) |
| Tonga | 0.86(0.58-1.24) | 0.99(0.62-1.43) |  | 1.24(0.71-1.71) | 1.27(0.72-1.76) | 0.95(0.70 to1.20) |
| Vanuatu | 1.87(1.11-2.63) | 1.43(0.80-2.10) |  | 4.50(2.41-6.08) | 1.73(0.90-2.40) | 0.60(0.43 to0.77) |
| Armenia | 116.25(95.03-142.29) | 3.32(2.72-4.03) |  | 69.40(57.71-81.67) | 2.34(1.90-2.83) | -1.57(-1.80 to-1.35) |
| Azerbaijan | 179.09(107.24-261.99) | 2.23(1.37-3.26) |  | 103.88(61.46-158.30) | 1.16(0.72-1.81) | -2.42(-2.57 to-2.28) |
| Georgia | 74.16(62.84-89.12) | 1.48(1.25-1.79) |  | 62.22(54.49-71.04) | 1.52(1.31-1.73) | -0.03(-0.31 to0.27) |
| Kazakhstan | 713.10(627.37-791.63) | 4.40(3.89-4.88) |  | 623.87(527.44-723.76) | 3.23(2.74-3.74) | -1.90(-2.57 to-1.23) |
| Kyrgyzstan | 134.61(118.50-154.70) | 2.58(2.29-2.91) |  | 115.47(99.42-132.42) | 1.73(1.49-1.99) | -1.87(-2.31 to-1.43) |
| Mongolia | 141.89(102.77-219.65) | 5.08(3.72-7.90) |  | 99.84(61.93-130.65) | 2.98(1.85-3.92) | -1.65(-1.79 to-1.52) |
| Tajikistan | 177.27(85.74-256.12) | 2.31(1.25-3.29) |  | 153.29(91.56-230.36) | 1.34(0.79-1.96) | -2.24(-2.48 to-2.00) |
| Turkmenistan | 108.19(91.29-128.15) | 2.26(1.94-2.60) |  | 98.74(81.67-119.72) | 1.93(1.59-2.33) | -0.89(-1.13 to-0.65) |
| Uzbekistan | 516.48(414.28-629.53) | 1.83(1.48-2.22) |  | 535.67(429.63-649.20) | 1.51(1.21-1.82) | -0.98(-1.19 to-0.76) |
| Albania | 38.24(23.95-49.57) | 1.08(0.67-1.37) |  | 15.94(9.37-21.81) | 0.63(0.39-0.88) | -1.76(-1.89 to-1.64) |
| Bosnia and Herzegovina | 15.29(8.30-18.97) | 0.35(0.19-0.43) |  | 15.18(7.33-20.30) | 0.36(0.18-0.48) | -0.26(-0.45 to-0.07) |
| Bulgaria | 211.11(194.41-228.20) | 2.62(2.41-2.83) |  | 125.94(103.75-148.37) | 1.50(1.26-1.76) | -2.18(-2.45 to-1.90) |
| Croatia | 57.16(54.17-60.57) | 1.15(1.09-1.22) |  | 48.24(42.01-54.56) | 0.78(0.68-0.90) | -1.22(-1.50 to-0.93) |
| Czechia | 302.83(286.75-320.66) | 2.96(2.80-3.12) |  | 190.48(165.69-217.78) | 1.24(1.07-1.42) | -2.64(-3.02 to-2.26) |
| Hungary | 340.23(320.53-360.94) | 3.11(2.91-3.30) |  | 216.53(187.31-249.78) | 1.56(1.34-1.81) | -2.38(-2.57 to-2.19) |
| North Macedonia | 16.72(10.51-20.85) | 0.94(0.59-1.17) |  | 10.01(5.49-12.64) | 0.40(0.22-0.50) | -2.26(-2.46 to-2.06) |
| Montenegro | 7.01(4.16-9.65) | 1.19(0.71-1.63) |  | 6.44(3.93-8.06) | 0.85(0.53-1.07) | -1.16(-1.43 to-0.88) |
| Poland | 825.61(806.44-846.13) | 2.27(2.22-2.33) |  | 535.18(489.00-581.65) | 1.09(0.99-1.18) | -2.17(-2.60 to-1.74) |
| Romania | 802.55(757.96-845.76) | 4.06(3.80-4.32) |  | 581.31(508.96-650.75) | 2.35(2.07-2.62) | -1.39(-1.96 to-0.81) |
| Serbia | 110.12(64.09-135.93) | 1.24(0.74-1.56) |  | 79.26(46.92-95.88) | 0.67(0.41-0.81) | -2.11(-2.33 to-1.90) |
| Slovakia | 142.81(87.17-188.02) | 2.73(1.69-3.60) |  | 121.26(75.93-154.16) | 1.84(1.21-2.33) | -1.12(-1.27 to-0.96) |
| Slovenia | 28.06(26.83-29.26) | 1.40(1.33-1.47) |  | 18.14(15.60-20.45) | 0.57(0.49-0.64) | -2.72(-3.14 to-2.30) |
| Belarus | 319.90(289.97-373.55) | 3.11(2.78-3.65) |  | 386.51(312.47-467.41) | 3.06(2.52-3.68) | -0.87(-1.53 to-0.21) |
| Estonia | 91.73(84.88-98.66) | 5.64(5.22-6.08) |  | 43.70(38.55-49.03) | 2.35(2.07-2.65) | -3.94(-4.67 to-3.21) |
| Latvia | 82.94(77.66-91.21) | 2.92(2.72-3.21) |  | 54.90(48.12-63.54) | 2.11(1.84-2.43) | -2.35(-2.83 to-1.87) |
| Lithuania | 139.55(130.65-150.11) | 3.72(3.48-4.01) |  | 126.66(111.86-141.64) | 3.29(2.93-3.68) | -1.31(-2.00 to-0.62) |
| Republic of Moldova | 207.51(193.70-222.35) | 4.82(4.50-5.19) |  | 218.71(191.73-246.42) | 4.97(4.27-5.71) | -0.38(-0.90 to0.15) |
| Russian Federation | 4480.04(4429.17-4529.02) | 2.93(2.89-2.97) |  | 6555.75(6032.81-7027.10) | 3.58(3.32-3.82) | -0.83(-1.68 to0.04) |
| Ukraine | 1559.80(1393.79-1875.33) | 3.03(2.67-3.59) |  | 1004.53(719.18-1342.77) | 2.12(1.62-2.71) | -2.11(-2.78 to-1.43) |
| Brunei Darussalam | 3.10(2.04-3.91) | 1.97(1.32-2.44) |  | 4.07(2.65-5.07) | 1.42(0.95-1.73) | -0.31(-0.59 to-0.04) |
| Japan | 2970.90(2796.81-3063.81) | 2.23(2.10-2.30) |  | 8786.11(7153.25-9676.16) | 2.09(1.84-2.22) | -0.86(-1.27 to-0.45) |
| Republic of Korea | 1203.20(678.67-1373.21) | 3.82(2.30-4.35) |  | 1303.50(955.76-1744.81) | 1.66(1.26-2.24) | -2.60(-2.86 to-2.35) |
| Singapore | 20.73(19.78-21.68) | 0.95(0.90-1.00) |  | 32.45(28.71-34.94) | 0.44(0.39-0.47) | -2.79(-3.02 to-2.56) |
| Australia | 151.16(144.07-158.15) | 0.93(0.88-0.97) |  | 233.56(208.79-249.20) | 0.63(0.58-0.68) | -0.66(-1.12 to-0.20) |
| New Zealand | 32.71(30.94-34.35) | 1.01(0.95-1.06) |  | 65.93(60.88-70.15) | 1.31(1.19-1.41) | 1.63(1.11 to2.16) |
| Andorra | 0.06(0.03-0.08) | 0.13(0.06-0.18) |  | 0.11(0.06-0.15) | 0.08(0.04-0.10) | -1.41(-1.56 to-1.26) |
| Austria | 80.87(76.81-84.57) | 0.91(0.87-0.94) |  | 108.93(96.77-117.22) | 0.64(0.58-0.68) | -0.55(-0.84 to-0.27) |
| Belgium | 230.76(216.67-241.73) | 2.09(1.98-2.19) |  | 666.48(571.54-726.96) | 2.75(2.44-2.97) | 1.27(1.06 to1.48) |
| Cyprus | 15.13(9.31-20.01) | 2.69(1.69-3.68) |  | 19.77(13.33-24.46) | 1.28(0.92-1.57) | -2.50(-2.69 to-2.31) |
| Denmark | 33.30(31.51-34.97) | 0.52(0.50-0.55) |  | 51.11(45.20-55.27) | 0.47(0.42-0.50) | -0.65(-0.92 to-0.38) |
| Finland | 66.49(62.72-69.17) | 1.12(1.06-1.16) |  | 109.15(94.77-117.26) | 0.96(0.87-1.02) | -0.57(-0.79 to-0.35) |
| France | 3863.66(3584.38-4063.10) | 5.16(4.83-5.39) |  | 4234.62(3630.89-4598.18) | 2.80(2.48-2.98) | -2.18(-2.37 to-2.00) |
| Germany | 993.58(948.10-1032.47) | 1.19(1.15-1.25) |  | 1488.69(1323.14-1595.86) | 0.82(0.75-0.86) | -0.54(-0.82 to-0.27) |
| Greece | 130.71(123.23-136.18) | 1.23(1.17-1.27) |  | 209.35(185.14-225.71) | 0.93(0.85-1.00) | -1.01(-1.30 to-0.73) |
| Iceland | 2.11(1.96-2.25) | 0.76(0.71-0.81) |  | 3.24(2.76-3.61) | 0.58(0.51-0.64) | -0.40(-0.62 to-0.17) |
| Ireland | 41.37(39.82-42.78) | 1.16(1.11-1.21) |  | 47.00(41.05-52.45) | 0.66(0.58-0.74) | -1.44(-1.62 to-1.26) |
| Israel | 88.57(83.98-92.99) | 1.89(1.79-1.98) |  | 194.37(165.39-211.58) | 1.49(1.29-1.61) | -0.53(-0.75 to-0.31) |
| Italy | 383.88(361.98-396.76) | 0.71(0.68-0.74) |  | 470.47(395.91-512.39) | 0.38(0.34-0.41) | -2.57(-2.89 to-2.26) |
| Luxembourg | 11.32(10.80-11.83) | 2.65(2.51-2.78) |  | 19.14(16.77-21.09) | 1.84(1.65-2.04) | -0.88(-1.09 to-0.67) |
| Malta | 3.47(3.23-3.69) | 0.96(0.89-1.02) |  | 7.40(6.35-8.28) | 0.96(0.85-1.08) | -0.06(-0.27 to0.14) |
| Netherlands | 124.42(115.56-129.55) | 0.76(0.71-0.79) |  | 201.10(176.75-216.50) | 0.63(0.57-0.68) | -0.45(-0.63 to-0.26) |
| Norway | 52.61(49.75-54.42) | 0.92(0.88-0.95) |  | 62.00(54.95-66.24) | 0.65(0.59-0.69) | -0.19(-0.70 to0.33) |
| Portugal | 185.40(177.67-192.48) | 2.06(1.97-2.16) |  | 430.45(372.49-472.46) | 1.75(1.57-1.90) | -0.44(-0.88 to-0.00) |
| Spain | 708.04(676.01-731.66) | 1.85(1.77-1.91) |  | 2684.16(2221.46-2969.01) | 2.31(2.00-2.51) | 1.53(1.02 to2.06) |
| Sweden | 150.54(141.24-157.62) | 1.17(1.11-1.21) |  | 110.84(96.11-122.77) | 0.53(0.47-0.58) | -2.31(-2.66 to-1.96) |
| Switzerland | 51.24(48.04-53.69) | 0.65(0.61-0.67) |  | 107.56(91.69-117.81) | 0.59(0.52-0.64) | -0.26(-0.48 to-0.03) |
| United Kingdom | 641.16(623.96-651.09) | 1.01(0.98-1.02) |  | 569.27(528.39-591.89) | 0.60(0.57-0.62) | -1.50(-1.67 to-1.34) |
| Argentina | 1808.36(1733.51-1887.35) | 5.55(5.32-5.79) |  | 792.28(742.11-835.79) | 1.71(1.59-1.83) | -3.75(-4.36 to-3.12) |
| Chile | 1283.37(1225.98-1339.05) | 10.09(9.69-10.50) |  | 293.56(273.07-309.58) | 1.36(1.28-1.44) | -6.46(-7.99 to-4.90) |
| Uruguay | 296.79(281.97-311.18) | 9.53(9.04-10.01) |  | 139.72(128.42-148.82) | 2.94(2.73-3.15) | -4.07(-4.74 to-3.39) |
| Canada | 352.86(335.13-366.03) | 1.25(1.19-1.30) |  | 568.56(505.63-611.44) | 0.98(0.88-1.05) | -0.67(-0.74 to-0.60) |
| United States of America | 4037.15(3820.76-4155.98) | 1.46(1.39-1.50) |  | 8430.55(7569.29-8896.89) | 1.97(1.82-2.09) | 1.38(1.18 to1.57) |
| Antigua and Barbuda | 1.04(0.95-1.14) | 1.81(1.65-1.97) |  | 1.10(1.04-1.15) | 1.31(1.24-1.37) | -1.15(-1.92 to-0.37) |
| Bahamas | 10.62(9.77-11.62) | 5.17(4.79-5.60) |  | 8.44(6.90-10.30) | 2.42(1.98-2.94) | -2.02(-2.74 to-1.30) |
| Barbados | 3.51(3.29-3.74) | 1.32(1.23-1.41) |  | 3.93(3.14-4.78) | 0.96(0.76-1.20) | -1.31(-2.12 to-0.50) |
| Belize | 9.44(8.51-10.56) | 4.36(4.00-4.74) |  | 7.83(6.96-8.77) | 2.32(2.07-2.60) | -2.11(-2.60 to-1.62) |
| Cuba | 244.38(235.02-253.82) | 2.50(2.40-2.60) |  | 210.42(184.72-236.17) | 1.29(1.13-1.44) | -2.53(-3.41 to-1.65) |
| Dominica | 1.18(0.60-1.40) | 1.74(0.88-2.03) |  | 0.94(0.61-1.37) | 1.55(1.02-2.29) | -0.23(-0.79 to0.33) |
| Dominican Republic | 329.55(193.14-392.75) | 4.19(2.45-4.90) |  | 297.25(150.71-378.77) | 2.88(1.47-3.67) | -0.56(-0.96 to-0.16) |
| Grenada | 3.36(3.00-3.75) | 3.75(3.40-4.14) |  | 2.89(2.51-3.22) | 2.98(2.60-3.30) | -1.06(-1.87 to-0.26) |
| Guyana | 40.21(35.11-45.71) | 5.37(4.84-5.95) |  | 22.82(17.62-29.34) | 3.40(2.62-4.30) | -1.20(-1.72 to-0.67) |
| Haiti | 524.58(209.92-754.17) | 6.07(3.21-8.44) |  | 442.54(225.42-642.92) | 3.43(2.10-5.02) | -1.64(-1.90 to-1.38) |
| Jamaica | 40.03(36.90-43.75) | 1.72(1.61-1.86) |  | 27.24(21.32-35.15) | 0.94(0.73-1.21) | -1.67(-2.44 to-0.89) |
| Saint Lucia | 3.43(3.10-3.77) | 3.09(2.88-3.31) |  | 3.06(2.52-3.63) | 1.60(1.31-1.94) | -2.58(-3.38 to-1.78) |
| Saint Vincent and the Grenadines | 2.49(2.21-2.81) | 2.54(2.30-2.81) |  | 3.04(2.65-3.49) | 2.60(2.24-3.02) | -0.39(-1.00 to0.22) |
| Suriname | 16.26(8.80-19.53) | 4.51(2.50-5.38) |  | 12.55(9.21-17.51) | 2.32(1.71-3.24) | -1.78(-2.23 to-1.33) |
| Trinidad and Tobago | 26.36(24.55-28.31) | 2.74(2.59-2.91) |  | 24.04(18.76-30.05) | 1.60(1.26-2.00) | -2.01(-2.69 to-1.33) |
| Bolivia (Plurinational State of) | 1049.86(697.19-1494.25) | 13.21(8.79-18.31) |  | 660.71(491.34-1053.37) | 6.06(4.52-9.69) | -2.63(-2.71 to-2.55) |
| Ecuador | 601.92(568.50-640.24) | 6.37(6.08-6.68) |  | 675.60(560.05-803.10) | 4.01(3.31-4.75) | -1.54(-2.17 to-0.91) |
| Peru | 4626.59(3045.75-5369.50) | 18.59(12.13-21.66) |  | 2137.34(1527.48-3004.39) | 6.18(4.45-8.71) | -3.97(-4.47 to-3.47) |
| Colombia | 626.79(571.90-685.98) | 1.85(1.72-2.00) |  | 390.40(323.29-465.41) | 0.80(0.65-0.96) | -2.32(-2.99 to-1.64) |
| Costa Rica | 40.36(38.28-42.83) | 1.50(1.43-1.58) |  | 44.17(38.90-48.91) | 0.91(0.80-1.01) | -1.84(-2.08 to-1.61) |
| El Salvador | 295.10(179.51-351.96) | 4.92(2.82-5.82) |  | 134.30(96.63-203.23) | 2.13(1.54-3.24) | -2.71(-3.13 to-2.30) |
| Guatemala | 747.99(699.04-801.78) | 8.80(8.47-9.23) |  | 594.72(507.33-689.43) | 4.21(3.60-4.88) | -2.66(-3.22 to-2.09) |
| Honduras | 490.82(318.44-631.59) | 9.41(5.48-11.29) |  | 507.52(283.70-687.82) | 6.63(3.48-8.73) | -1.10(-1.15 to-1.06) |
| Mexico | 3139.28(2966.76-3376.69) | 3.68(3.53-3.88) |  | 2424.15(2118.76-2759.63) | 2.05(1.77-2.36) | -2.05(-2.63 to-1.46) |
| Nicaragua | 180.68(116.84-225.50) | 3.49(2.17-4.21) |  | 85.93(58.83-121.03) | 1.45(0.99-2.02) | -2.81(-2.89 to-2.73) |
| Panama | 79.24(71.76-87.67) | 3.38(3.11-3.67) |  | 88.97(72.97-106.07) | 2.14(1.76-2.56) | -1.88(-2.12 to-1.65) |
| Venezuela (Bolivarian Republic of) | 904.84(854.71-960.20) | 4.50(4.29-4.73) |  | 978.09(749.35-1248.24) | 3.86(2.96-4.93) | -0.44(-0.86 to-0.01) |
| Brazil | 2157.44(1996.63-2308.15) | 1.61(1.51-1.71) |  | 4617.66(4222.01-4906.99) | 2.10(1.92-2.24) | 0.78(0.48 to1.09) |
| Paraguay | 95.89(69.62-126.00) | 2.19(1.57-2.91) |  | 159.61(105.18-209.61) | 2.65(1.74-3.45) | 0.87(0.69 to1.05) |
| Algeria | 393.81(201.89-550.97) | 1.32(0.70-1.77) |  | 234.10(138.52-294.87) | 0.61(0.35-0.75) | -2.31(-2.38 to-2.23) |
| Bahrain | 3.52(1.71-4.15) | 0.99(0.47-1.14) |  | 4.51(2.72-5.74) | 0.46(0.29-0.57) | -2.63(-2.76 to-2.50) |
| Egypt | 938.01(477.85-1123.23) | 1.78(0.82-2.07) |  | 340.49(248.90-488.78) | 0.37(0.28-0.56) | -5.67(-6.19 to-5.15) |
| Iran (Islamic Republic of) | 973.32(512.92-1219.45) | 1.41(0.77-1.75) |  | 320.93(197.94-408.53) | 0.43(0.26-0.54) | -3.05(-3.49 to-2.62) |
| Iraq | 270.32(155.04-355.66) | 1.20(0.68-1.55) |  | 244.49(154.04-325.15) | 0.70(0.45-0.92) | -1.78(-1.87 to-1.68) |
| Jordan | 51.10(34.80-61.80) | 1.30(0.87-1.58) |  | 64.39(44.31-82.95) | 0.66(0.45-0.85) | -2.52(-2.75 to-2.28) |
| Kuwait | 18.56(17.19-19.99) | 1.33(1.24-1.43) |  | 10.32(8.52-12.26) | 0.32(0.26-0.38) | -4.13(-4.71 to-3.54) |
| Lebanon | 22.72(12.42-29.24) | 0.77(0.43-0.99) |  | 24.49(14.38-29.67) | 0.43(0.26-0.53) | -1.74(-1.86 to-1.61) |
| Libya | 43.12(24.81-55.29) | 0.92(0.53-1.18) |  | 47.02(25.63-65.35) | 0.86(0.48-1.20) | 0.42(0.15 to0.69) |
| Morocco | 448.85(213.63-637.68) | 1.44(0.73-2.00) |  | 211.37(120.04-280.92) | 0.64(0.37-0.84) | -2.56(-2.64 to-2.49) |
| Palestine | 19.35(11.58-25.64) | 0.80(0.48-1.05) |  | 17.99(10.84-22.90) | 0.44(0.26-0.54) | -1.78(-1.94 to-1.62) |
| Oman | 8.20(4.48-11.47) | 0.43(0.23-0.59) |  | 7.67(4.27-9.86) | 0.24(0.13-0.30) | -1.04(-1.33 to-0.75) |
| Qatar | 1.25(0.55-1.75) | 0.46(0.19-0.64) |  | 3.97(2.31-5.52) | 0.26(0.16-0.35) | -1.50(-2.06 to-0.94) |
| Saudi Arabia | 259.87(150.19-363.67) | 1.63(0.92-2.23) |  | 299.18(186.53-416.68) | 0.95(0.59-1.26) | -1.69(-1.80 to-1.58) |
| Syrian Arab Republic | 218.99(129.93-286.96) | 1.59(0.94-2.02) |  | 112.66(70.03-151.15) | 0.95(0.58-1.26) | -1.74(-2.17 to-1.30) |
| Tunisia | 102.65(54.73-139.04) | 1.12(0.59-1.50) |  | 57.66(33.44-79.57) | 0.53(0.30-0.73) | -2.45(-2.58 to-2.32) |
| Türkiye | 408.75(249.19-562.82) | 0.72(0.44-0.97) |  | 317.97(195.19-390.25) | 0.42(0.26-0.52) | -1.38(-1.60 to-1.15) |
| United Arab Emirates | 10.24(6.16-15.34) | 0.77(0.46-1.11) |  | 17.16(9.49-21.80) | 0.39(0.24-0.48) | -1.06(-1.46 to-0.66) |
| Yemen | 388.20(169.28-583.04) | 1.72(0.79-2.50) |  | 291.56(151.83-388.60) | 0.84(0.43-1.14) | -2.40(-2.46 to-2.34) |
| Afghanistan | 377.03(185.51-551.73) | 2.64(1.38-3.67) |  | 469.12(264.40-645.77) | 1.31(0.77-1.76) | -2.08(-2.24 to-1.91) |
| Bangladesh | 48.75(8.59-61.91) | 0.15(0.01-0.20) |  | 130.92(5.81-172.14) | 0.15(0.00-0.20) | -0.29(-0.61 to0.04) |
| Bhutan | 2.04(0.18-2.98) | 0.33(0.04-0.45) |  | 1.18(0.25-1.59) | 0.21(0.05-0.29) | -1.62(-1.74 to-1.51) |
| India | 5879.89(2655.22-7188.72) | 0.77(0.37-0.90) |  | 5811.31(3179.30-6919.47) | 0.59(0.33-0.69) | -0.83(-0.98 to-0.69) |
| Nepal | 78.83(9.92-111.81) | 0.34(0.05-0.45) |  | 45.61(8.11-62.98) | 0.21(0.04-0.28) | -1.51(-1.68 to-1.34) |
| Pakistan | 539.51(67.42-727.76) | 0.40(0.06-0.51) |  | 588.70(99.86-759.33) | 0.32(0.06-0.40) | -0.49(-0.65 to-0.33) |
| Angola | 373.32(166.06-536.12) | 2.29(1.23-3.16) |  | 467.07(223.72-645.51) | 1.24(0.62-1.72) | -2.01(-2.15 to-1.86) |
| Central African Republic | 95.23(48.45-139.56) | 2.35(1.31-3.34) |  | 111.34(56.51-160.37) | 1.75(0.92-2.47) | -0.90(-0.97 to-0.83) |
| Congo | 57.22(29.29-76.87) | 1.98(1.03-2.53) |  | 55.06(25.03-74.74) | 1.19(0.58-1.64) | -1.97(-2.16 to-1.77) |
| Democratic Republic of the Congo | 1189.18(550.96-1715.56) | 1.97(0.94-2.71) |  | 994.39(462.69-1485.53) | 1.10(0.57-1.69) | -1.75(-1.88 to-1.61) |
| Equatorial Guinea | 12.62(6.43-17.93) | 2.00(1.06-2.74) |  | 16.12(6.13-24.96) | 1.21(0.51-1.85) | -1.90(-2.06 to-1.74) |
| Gabon | 19.57(9.16-25.41) | 1.73(0.82-2.19) |  | 18.22(7.12-25.95) | 1.17(0.51-1.61) | -1.11(-1.28 to-0.94) |
| Burundi | 104.88(49.67-156.05) | 1.40(0.75-1.99) |  | 87.01(40.07-154.26) | 0.74(0.44-1.23) | -2.09(-2.21 to-1.98) |
| Comoros | 7.24(3.81-10.17) | 1.23(0.76-1.66) |  | 5.36(3.72-7.68) | 0.89(0.64-1.25) | -1.18(-1.28 to-1.08) |
| Djibouti | 4.18(2.49-5.76) | 0.95(0.63-1.29) |  | 6.12(3.85-9.37) | 0.72(0.49-1.06) | -1.07(-1.27 to-0.87) |
| Eritrea | 41.38(24.22-59.72) | 1.11(0.72-1.49) |  | 46.15(27.91-77.66) | 0.94(0.61-1.47) | -0.55(-0.62 to-0.49) |
| Ethiopia | 861.51(542.05-1163.75) | 1.33(0.93-1.71) |  | 532.07(343.99-946.99) | 0.60(0.41-0.94) | -2.99(-3.19 to-2.79) |
| Kenya | 180.37(116.89-240.93) | 0.67(0.48-0.97) |  | 184.58(132.37-285.41) | 0.61(0.44-0.90) | 0.01(-0.11 to0.13) |
| Madagascar | 189.64(115.42-247.96) | 1.15(0.73-1.52) |  | 184.75(114.98-260.97) | 0.75(0.50-1.04) | -1.27(-1.36 to-1.17) |
| Malawi | 247.31(128.68-345.32) | 1.57(0.92-2.07) |  | 137.43(72.28-202.90) | 0.86(0.54-1.19) | -1.92(-2.00 to-1.84) |
| Mauritius | 9.34(8.96-9.73) | 1.20(1.14-1.25) |  | 14.01(12.91-14.75) | 0.98(0.90-1.04) | -1.96(-2.88 to-1.04) |
| Mozambique | 289.74(181.22-429.17) | 1.50(1.03-2.08) |  | 262.78(169.90-477.36) | 0.95(0.67-1.45) | -1.11(-1.26 to-0.96) |
| Rwanda | 134.87(72.11-188.26) | 1.53(0.94-2.01) |  | 80.45(47.86-143.29) | 0.81(0.55-1.26) | -2.83(-3.10 to-2.56) |
| Seychelles | 0.90(0.49-1.04) | 1.45(0.78-1.67) |  | 1.03(0.66-1.30) | 1.04(0.68-1.31) | -1.03(-1.24 to-0.83) |
| Somalia | 122.42(69.17-177.87) | 1.20(0.77-1.68) |  | 201.16(127.18-304.09) | 0.94(0.62-1.37) | -0.62(-0.77 to-0.47) |
| United Republic of Tanzania | 519.71(315.84-683.57) | 1.37(0.87-1.73) |  | 486.98(292.23-964.47) | 0.85(0.58-1.42) | -1.41(-1.47 to-1.35) |
| Uganda | 273.87(138.71-399.81) | 1.03(0.61-1.40) |  | 346.89(163.89-578.99) | 0.84(0.51-1.27) | -0.85(-1.01 to-0.70) |
| Zambia | 151.05(91.83-198.93) | 1.30(0.86-1.62) |  | 125.68(73.83-207.86) | 0.79(0.50-1.13) | -1.73(-1.84 to-1.62) |
| Botswana | 15.29(10.45-24.10) | 1.31(0.89-2.18) |  | 26.44(18.08-41.80) | 1.22(0.86-1.88) | -0.35(-0.58 to-0.13) |
| Lesotho | 16.21(11.74-25.89) | 1.08(0.78-1.70) |  | 33.67(22.20-48.33) | 2.00(1.33-2.87) | 2.65(2.20 to3.11) |
| Namibia | 15.68(10.71-22.05) | 1.23(0.87-1.78) |  | 30.64(20.91-54.08) | 1.44(0.99-2.44) | 0.40(0.12 to0.68) |
| South Africa | 758.95(442.27-870.53) | 2.05(1.18-2.37) |  | 880.39(524.07-1103.21) | 1.64(0.98-2.03) | -0.91(-1.18 to-0.65) |
| Eswatini | 11.59(8.40-17.94) | 1.49(1.07-2.29) |  | 20.72(13.75-33.34) | 1.99(1.34-3.16) | 1.31(0.76 to1.87) |
| Zimbabwe | 137.61(67.37-169.59) | 1.58(0.75-1.97) |  | 323.09(179.45-441.33) | 2.42(1.26-3.23) | 1.76(1.31 to2.21) |
| Benin | 82.46(48.94-117.83) | 1.15(0.76-1.53) |  | 215.60(87.41-315.64) | 1.53(0.65-2.08) | 0.75(0.36 to1.13) |
| Burkina Faso | 219.44(119.46-302.24) | 1.54(0.95-1.97) |  | 324.05(155.22-615.93) | 1.15(0.63-1.99) | -0.82(-0.94 to-0.70) |
| Cameroon | 140.98(85.00-189.32) | 1.07(0.70-1.35) |  | 486.44(189.79-662.58) | 1.71(0.69-2.27) | 1.31(0.80 to1.82) |
| Cabo Verde | 0.15(0.05-0.95) | 0.04(0.01-0.23) |  | 5.83(0.74-7.66) | 1.23(0.15-1.61) | 9.57(6.04 to13.21) |
| Chad | 85.57(53.83-120.50) | 0.97(0.67-1.40) |  | 351.43(171.75-479.63) | 1.71(0.84-2.32) | 1.64(1.17 to2.12) |
| Côte d'Ivoire | 146.85(80.86-209.10) | 0.97(0.57-1.33) |  | 379.77(151.39-543.69) | 1.52(0.64-2.07) | 1.22(0.75 to1.69) |
| Gambia | 9.49(5.83-14.23) | 0.84(0.55-1.18) |  | 25.67(13.27-38.04) | 1.43(0.77-1.94) | 1.30(0.84 to1.77) |
| Ghana | 158.90(109.15-223.21) | 0.83(0.59-1.27) |  | 214.79(143.76-352.53) | 0.68(0.48-1.14) | -0.35(-0.53 to-0.17) |
| Guinea | 122.04(68.88-180.13) | 1.32(0.85-1.87) |  | 226.32(95.29-307.95) | 1.65(0.71-2.24) | 0.70(0.23 to1.16) |
| Guinea-Bissau | 18.44(10.34-27.46) | 1.46(0.92-2.08) |  | 27.53(13.56-36.17) | 1.80(0.93-2.30) | 0.46(-0.01 to0.93) |
| Liberia | 60.40(32.10-86.99) | 1.60(0.93-2.21) |  | 67.55(26.26-101.07) | 1.47(0.59-2.15) | -0.64(-0.88 to-0.41) |
| Mali | 160.23(90.18-247.92) | 1.30(0.86-1.85) |  | 399.26(174.97-539.37) | 1.61(0.75-2.12) | 0.51(0.10 to0.91) |
| Mauritania | 18.66(13.19-26.07) | 0.84(0.63-1.20) |  | 40.32(20.76-54.84) | 1.16(0.59-1.53) | 0.57(0.09 to1.04) |
| Niger | 178.67(99.18-271.91) | 1.37(0.86-2.00) |  | 352.48(150.68-528.77) | 1.33(0.60-1.92) | -0.55(-0.91 to-0.20) |
| Nigeria | 1276.77(759.01-1773.22) | 1.08(0.70-1.44) |  | 4044.71(1514.23-5772.52) | 1.70(0.70-2.28) | 1.37(0.97 to1.77) |
| Sao Tome and Principe | 4.20(3.03-5.79) | 2.85(2.08-3.80) |  | 2.65(1.75-4.00) | 1.75(1.21-2.43) | -1.71(-1.86 to-1.57) |
| Senegal | 104.83(61.86-144.03) | 1.04(0.66-1.40) |  | 171.32(85.32-252.17) | 1.34(0.65-1.90) | 0.57(0.15 to0.99) |
| Sierra Leone | 96.98(50.73-144.83) | 1.49(0.89-2.19) |  | 155.51(68.86-225.32) | 1.73(0.81-2.38) | 0.10(-0.30 to0.50) |
| Togo | 39.82(23.77-53.41) | 0.90(0.59-1.18) |  | 93.53(38.09-133.69) | 1.47(0.60-2.06) | 1.38(0.95 to1.81) |
| American Samoa | 1.46(0.95-2.01) | 3.52(2.22-4.82) |  | 1.80(1.11-2.38) | 4.22(2.62-5.56) | 0.84(0.71 to0.97) |
| Bermuda | 0.94(0.89-1.00) | 1.67(1.56-1.78) |  | 0.92(0.78-1.10) | 0.80(0.66-0.96) | -2.58(-3.49 to-1.66) |
| Cook Islands | 0.18(0.12-0.23) | 1.15(0.75-1.44) |  | 0.16(0.11-0.21) | 0.81(0.54-1.05) | -1.62(-1.87 to-1.36) |
| Greenland | 2.02(1.33-2.54) | 4.30(2.95-5.29) |  | 1.40(0.97-1.82) | 2.75(1.97-3.51) | -1.19(-1.40 to-0.99) |
| Guam | 0.90(0.56-1.22) | 0.79(0.47-1.04) |  | 1.46(0.79-1.78) | 0.89(0.49-1.10) | 1.16(0.88 to1.43) |
| Monaco | 0.65(0.42-0.81) | 1.26(0.82-1.57) |  | 1.47(0.95-2.17) | 1.76(1.15-2.55) | 1.04(0.81 to1.26) |
| Nauru | 0.21(0.12-0.28) | 2.37(1.37-3.16) |  | 0.26(0.14-0.35) | 2.76(1.49-3.59) | 0.56(0.18 to0.94) |
| Niue | 0.04(0.02-0.05) | 1.65(1.07-2.30) |  | 0.06(0.04-0.07) | 3.92(2.51-4.97) | 1.48(0.99 to1.97) |
| Northern Mariana Islands | 0.49(0.32-0.75) | 1.50(1.03-2.33) |  | 1.13(0.60-1.34) | 2.75(1.53-3.23) | 2.57(2.23 to2.92) |
| Palau | 0.42(0.25-0.57) | 3.16(1.86-4.20) |  | 0.52(0.31-0.66) | 3.21(1.99-4.01) | 0.25(0.12 to0.37) |
| Puerto Rico | 88.70(84.86-92.13) | 2.62(2.50-2.71) |  | 101.15(84.40-117.52) | 1.59(1.33-1.84) | -1.89(-2.78 to-1.00) |
| Saint Kitts and Nevis | 0.79(0.73-0.84) | 2.01(1.88-2.14) |  | 0.65(0.54-0.76) | 1.23(1.03-1.44) | -1.65(-2.57 to-0.73) |
| San Marino | 0.22(0.14-0.28) | 0.84(0.53-1.05) |  | 0.31(0.19-0.44) | 0.41(0.26-0.57) | -1.54(-1.82 to-1.26) |
| Tokelau | 0.02(0.02-0.03) | 1.59(1.03-2.23) |  | 0.04(0.03-0.05) | 3.46(2.17-4.43) | 0.70(0.03 to1.38) |
| Tuvalu | 0.24(0.13-0.35) | 2.26(1.34-3.11) |  | 0.21(0.12-0.27) | 1.89(1.05-2.48) | -0.42(-0.47 to-0.36) |
| United States Virgin Islands | 2.06(1.11-2.52) | 2.35(1.29-2.85) |  | 1.84(1.13-2.53) | 1.55(0.96-2.13) | -0.94(-1.26 to-0.62) |
| South Sudan | 110.14(62.16-156.29) | 1.34(0.81-1.85) |  | 129.56(70.76-198.86) | 1.12(0.68-1.69) | -0.47(-0.88 to-0.06) |
| Sudan | 784.26(329.97-1211.55) | 2.53(1.17-3.80) |  | 423.41(205.31-586.00) | 0.93(0.46-1.28) | -3.09(-3.18 to-2.99) |

^a^Age-standardized death rate, ^b^ Uncertainty interval, ^c^ Estimated annual percentage change, ^d^Confidence interval,
