# Supplementary material for: Global, regional, and national assessment of foreign body aspiration (1990–2021): novel insights into incidence, mortality, and disability-adjusted life years
Source: Scand J Trauma Resusc Emerg Med. 2025 Mar 11;33:40. doi: 10.1186/s13049-025-01352-z (PMC11895196; doi:10.1186/s13049-025-01352-z)
Supplement: Supplementary file 8 — Supplementary Material 8: Table S4 DALYs and age‑standardized DALYs rate for foreign body aspiration in 1990 and 2021, and temporal trends across 204 countries. [file 13049_2025_1352_MOESM8_ESM.docx]

| **S4** **Table** DALYs^a^ and age‑standardized DALYs^a^ rate for foreign body aspiration in 1990 and 2021, and temporal trends | | | | | | |
| --- | --- | --- | --- | --- | --- | --- |
|  | **1990** | |  | **2021** | | **1990-2021 EAPC**^c^**（95%CI**^d^**）** |
|  | **DALYs**^a^ **number (95%UI**^b^**)** | **Age-standardized DALYs**^a^ **rate per 100,000 (95% UI**^b^**)** |  | **DALYs**^a^ **number (95%UI**^b^**)** | **Age-standardized DALYs**^a^ **rate per 100,000 (95% UI**^b^**)** |  |
| China | 1924610.85(1532055.78-3009107.36) | 174.47(138.93-272.70) |  | 619544.51(379102.51-748821.36) | 77.14(49.67-94.27) | -3.31(-3.60 to-3.03) |
| Democratic People's Republic of Korea | 31146.55(20260.24-50581.47) | 128.78(84.51-207.94) |  | 13833.81(9107.90-22648.69) | 74.32(46.95-128.74) | -1.31(-1.63 to-0.99) |
| Taiwan (Province of China) | 24513.93(23162.57-25984.88) | 144.84(136.69-153.83) |  | 13367.87(12110.25-14402.49) | 86.64(75.08-97.50) | -1.56(-1.90 to-1.21) |
| Cambodia | 17257.43(7193.90-27740.65) | 109.40(57.37-173.60) |  | 10371.46(7043.52-16187.38) | 64.13(43.84-99.38) | -2.12(-2.29 to-1.95) |
| Indonesia | 146912.29(35679.39-193576.15) | 69.67(17.37-91.05) |  | 79415.48(22549.82-100868.94) | 37.10(9.91-47.23) | -2.24(-2.36 to-2.13) |
| Lao People's Democratic Republic | 9148.88(3125.28-15281.32) | 141.51(59.82-227.92) |  | 5614.53(3587.67-8833.96) | 73.29(47.74-113.81) | -2.22(-2.33 to-2.10) |
| Malaysia | 16921.29(10183.01-21335.37) | 92.73(54.74-115.92) |  | 17978.42(13016.45-24820.43) | 63.65(47.64-88.91) | -1.42(-1.71 to-1.14) |
| Maldives | 829.94(373.26-1164.76) | 263.72(135.65-354.09) |  | 349.44(256.97-485.09) | 93.82(70.07-128.00) | -3.14(-3.23 to-3.05) |
| Myanmar | 64539.10(25015.68-104151.49) | 135.59(57.95-215.08) |  | 41141.65(27687.43-60550.37) | 78.85(53.13-116.07) | -2.01(-2.22 to-1.81) |
| Philippines | 51307.89(32621.83-63052.36) | 65.55(41.38-78.08) |  | 52201.89(31353.73-61816.96) | 48.44(29.71-57.07) | -0.55(-0.77 to-0.32) |
| Sri Lanka | 20601.87(10985.36-23652.61) | 126.11(68.04-144.36) |  | 10912.78(7131.78-16189.71) | 52.66(35.17-78.18) | -3.61(-4.07 to-3.15) |
| Thailand | 26284.71(19529.90-42104.87) | 55.36(41.07-82.98) |  | 43843.75(26664.06-56105.35) | 70.58(46.73-88.61) | 1.13(0.68 to1.58) |
| Timor-Leste | 1224.78(478.39-1998.43) | 94.39(44.58-147.12) |  | 965.89(679.61-1481.65) | 58.59(42.15-89.70) | -1.64(-1.92 to-1.36) |
| Viet Nam | 92151.58(39128.31-122681.73) | 108.99(49.09-141.47) |  | 55144.38(29432.62-72533.16) | 68.92(34.87-91.61) | -1.41(-1.51 to-1.31) |
| Fiji | 1235.05(874.31-1677.18) | 154.30(107.12-207.91) |  | 1489.41(970.93-2011.30) | 166.34(108.41-223.82) | 0.30(0.07 to0.54) |
| Kiribati | 18.27(11.41-24.55) | 17.89(11.39-23.64) |  | 16.96(10.47-25.68) | 13.19(8.21-19.61) | -0.86(-0.97 to-0.74) |
| Marshall Islands | 43.45(28.05-57.91) | 88.57(55.36-115.10) |  | 60.03(32.82-81.52) | 107.90(58.97-146.61) | 0.76(0.47 to1.05) |
| Micronesia (Federated States of) | 118.11(73.84-167.83) | 101.96(62.62-142.56) |  | 88.37(47.94-120.27) | 88.84(47.90-120.68) | -0.42(-0.48 to-0.37) |
| Papua New Guinea | 6059.61(3437.91-8963.07) | 106.68(59.94-153.80) |  | 15626.13(7995.88-21920.21) | 115.67(59.80-158.80) | 0.41(0.15 to0.68) |
| Samoa | 143.74(100.92-200.41) | 72.33(48.51-104.71) |  | 158.01(94.35-213.52) | 70.88(41.07-96.09) | 0.10(0.05 to0.16) |
| Solomon Islands | 345.92(190.81-509.56) | 82.27(42.19-117.37) |  | 634.21(345.80-945.39) | 86.73(45.99-128.44) | 0.28(0.12 to0.44) |
| Tonga | 57.66(40.45-79.83) | 49.51(33.80-70.79) |  | 67.97(40.16-94.43) | 59.68(34.45-82.32) | 0.75(0.46 to1.04) |
| Vanuatu | 130.14(78.83-184.51) | 71.43(41.90-101.02) |  | 277.72(153.20-379.99) | 84.71(46.04-113.90) | 0.56(0.30 to0.82) |
| Armenia | 8902.82(7202.38-10913.85) | 246.44(199.61-301.56) |  | 3469.53(2816.32-4202.00) | 142.01(111.83-177.96) | -2.19(-2.41 to-1.98) |
| Azerbaijan | 14651.58(9044.48-21301.75) | 174.74(108.98-252.00) |  | 6985.37(4489.60-10590.80) | 83.02(53.64-128.38) | -2.74(-2.87 to-2.61) |
| Georgia | 5217.85(4379.91-6243.72) | 109.60(91.61-131.75) |  | 2843.93(2476.39-3239.41) | 83.34(70.93-97.06) | -1.07(-1.36 to-0.78) |
| Kazakhstan | 47825.85(41849.81-53663.77) | 278.77(244.56-311.74) |  | 36679.93(31031.52-43025.87) | 185.99(157.52-218.09) | -2.29(-2.97 to-1.60) |
| Kyrgyzstan | 10687.14(9337.07-12348.01) | 188.94(166.58-216.84) |  | 7596.58(6589.47-8689.41) | 105.63(91.72-120.79) | -2.47(-2.92 to-2.02) |
| Mongolia | 11780.88(8503.31-17744.40) | 382.07(279.11-591.93) |  | 6655.38(4215.54-8736.19) | 186.46(117.99-245.34) | -2.23(-2.39 to-2.07) |
| Tajikistan | 14878.64(6979.82-21375.99) | 176.26(89.49-251.64) |  | 12109.76(7446.52-18661.11) | 97.60(61.21-146.85) | -2.35(-2.61 to-2.09) |
| Turkmenistan | 9002.68(7535.00-10714.42) | 171.43(145.61-201.69) |  | 6428.83(5356.64-7676.48) | 121.57(101.28-144.96) | -1.46(-1.71 to-1.22) |
| Uzbekistan | 43557.90(34864.07-52935.40) | 143.57(116.40-173.52) |  | 39090.79(30734.04-48191.90) | 105.46(83.20-129.40) | -1.34(-1.56 to-1.13) |
| Albania | 3183.88(2075.58-4086.58) | 85.13(55.44-108.52) |  | 849.62(539.03-1159.42) | 41.51(26.56-57.76) | -2.43(-2.55 to-2.31) |
| Bosnia and Herzegovina | 1091.74(780.04-1326.25) | 24.85(17.82-30.42) |  | 694.76(399.67-892.39) | 20.54(12.59-26.74) | -0.96(-1.16 to-0.76) |
| Bulgaria | 11802.47(10907.56-12787.20) | 168.97(155.81-183.18) |  | 5028.24(4220.81-5850.91) | 79.52(66.79-93.07) | -2.84(-3.14 to-2.54) |
| Croatia | 2901.91(2738.32-3081.00) | 65.34(61.31-69.78) |  | 1706.32(1487.92-1923.83) | 37.35(32.45-42.85) | -1.76(-2.03 to-1.49) |
| Czechia | 15812.05(14970.66-16714.51) | 175.72(164.59-186.97) |  | 6494.33(5634.63-7403.04) | 56.09(48.48-64.55) | -3.48(-3.81 to-3.15) |
| Hungary | 16367.98(15341.52-17444.66) | 172.90(160.83-185.63) |  | 7506.05(6484.50-8687.06) | 70.55(60.46-82.07) | -2.95(-3.15 to-2.76) |
| North Macedonia | 1265.00(850.49-1554.31) | 71.96(48.31-88.48) |  | 445.94(272.42-549.94) | 20.73(12.91-25.78) | -3.30(-3.59 to-3.02) |
| Montenegro | 444.47(288.18-601.13) | 77.90(50.91-104.77) |  | 264.13(170.31-329.72) | 40.51(27.16-51.80) | -2.18(-2.51 to-1.86) |
| Poland | 50302.93(48722.87-51962.46) | 147.14(142.21-152.38) |  | 21454.83(19750.96-23188.52) | 52.85(48.54-57.03) | -3.09(-3.43 to-2.75) |
| Romania | 53739.76(50120.07-57392.18) | 294.83(273.01-317.59) |  | 22480.30(19836.70-25114.51) | 115.37(101.95-128.32) | -2.59(-3.19 to-1.99) |
| Serbia | 6859.36(4246.58-8486.65) | 83.42(51.39-104.23) |  | 3004.43(1874.21-3638.17) | 32.19(21.18-39.33) | -3.21(-3.48 to-2.93) |
| Slovakia | 7510.45(4799.78-9872.88) | 152.22(98.55-200.64) |  | 5066.82(3345.06-6350.47) | 95.26(67.60-120.75) | -1.38(-1.55 to-1.22) |
| Slovenia | 1406.59(1337.65-1475.13) | 78.65(73.94-83.29) |  | 614.35(537.17-685.87) | 26.24(22.98-29.32) | -3.28(-3.69 to-2.86) |
| Belarus | 17247.89(15319.87-20200.69) | 186.19(163.43-215.12) |  | 14697.09(12033.15-17695.67) | 138.55(114.49-165.38) | -1.78(-2.40 to-1.16) |
| Estonia | 4674.77(4339.66-5039.51) | 320.19(296.31-346.29) |  | 1509.69(1334.71-1698.81) | 101.72(90.33-114.14) | -4.71(-5.41 to-4.01) |
| Latvia | 4117.64(3826.68-4514.98) | 161.55(149.65-177.35) |  | 2020.16(1765.16-2326.83) | 93.22(81.89-106.23) | -3.09(-3.53 to-2.64) |
| Lithuania | 7343.03(6842.39-7894.36) | 213.13(198.19-229.52) |  | 4543.66(4040.95-5087.97) | 145.27(130.83-161.23) | -2.15(-2.75 to-1.55) |
| Republic of Moldova | 12947.50(11899.74-14201.19) | 307.40(281.70-339.37) |  | 8784.59(7617.63-10020.66) | 249.51(209.77-296.24) | -1.18(-1.60 to-0.77) |
| Russian Federation | 238099.07(234682.87-242133.61) | 169.14(166.25-172.15) |  | 250135.72(231946.00-267446.48) | 164.56(154.69-174.62) | -1.43(-2.26 to-0.60) |
| Ukraine | 85193.38(74803.03-101421.39) | 188.70(163.71-222.65) |  | 44563.53(33030.43-58646.05) | 119.06(95.43-145.22) | -2.29(-2.86 to-1.71) |
| Brunei Darussalam | 188.43(127.60-246.78) | 76.08(51.13-96.07) |  | 189.34(132.61-239.33) | 55.19(39.10-70.82) | -0.43(-0.64 to-0.23) |
| Japan | 92145.70(89463.01-94362.93) | 89.96(87.46-92.58) |  | 135721.96(118321.11-145084.85) | 60.74(56.70-64.12) | -1.64(-1.94 to-1.34) |
| Republic of Korea | 65583.86(37689.11-77693.55) | 183.60(109.19-218.53) |  | 26160.34(20958.37-38224.96) | 47.74(38.60-73.00) | -4.34(-4.64 to-4.04) |
| Singapore | 992.40(933.85-1059.22) | 39.94(37.50-42.60) |  | 809.03(744.12-867.94) | 13.98(12.58-15.39) | -3.47(-3.67 to-3.27) |
| Australia | 7574.17(7188.08-7948.55) | 50.49(47.81-53.30) |  | 6952.21(6471.80-7434.20) | 27.99(25.49-30.78) | -1.26(-1.70 to-0.82) |
| New Zealand | 2215.69(2070.30-2349.27) | 71.54(66.74-76.15) |  | 3352.41(3063.04-3621.27) | 86.28(76.99-94.87) | 1.52(0.94 to2.10) |
| Andorra | 3.40(2.26-4.44) | 7.81(5.03-10.19) |  | 3.44(2.36-4.37) | 3.59(2.53-4.55) | -2.21(-2.36 to-2.06) |
| Austria | 3020.15(2895.90-3140.22) | 44.09(41.82-46.32) |  | 2436.20(2263.77-2575.94) | 21.88(20.36-23.40) | -1.61(-1.92 to-1.29) |
| Belgium | 9072.15(8661.57-9509.52) | 107.31(101.43-113.63) |  | 12467.98(11345.90-13368.68) | 79.54(73.37-86.07) | -0.58(-0.83 to-0.33) |
| Cyprus | 621.83(387.53-811.48) | 92.35(57.04-120.37) |  | 481.43(327.33-618.48) | 33.81(24.16-43.18) | -3.33(-3.46 to-3.20) |
| Denmark | 1180.81(1119.90-1241.79) | 25.02(23.51-26.57) |  | 1134.59(1048.52-1218.89) | 15.50(14.27-16.84) | -1.62(-1.83 to-1.41) |
| Finland | 2368.62(2277.87-2468.25) | 47.84(45.61-50.10) |  | 2380.60(2187.35-2520.07) | 33.16(30.56-35.56) | -1.23(-1.46 to-0.99) |
| France | 102471.13(98284.53-106533.30) | 178.84(171.71-186.27) |  | 77641.05(70217.42-82279.67) | 86.15(79.72-91.60) | -2.39(-2.68 to-2.11) |
| Germany | 43436.80(41845.20-45233.56) | 69.58(66.33-73.51) |  | 31046.77(28935.27-32755.11) | 25.94(24.52-27.36) | -2.38(-2.68 to-2.08) |
| Greece | 5078.21(4840.53-5279.83) | 60.79(57.22-64.11) |  | 4305.85(3993.41-4574.25) | 33.49(30.87-36.31) | -2.02(-2.44 to-1.59) |
| Iceland | 81.26(76.13-86.83) | 32.30(30.13-34.63) |  | 79.62(71.94-87.77) | 19.93(17.93-22.20) | -1.11(-1.30 to-0.93) |
| Ireland | 1618.43(1554.76-1691.84) | 48.50(46.45-50.97) |  | 1200.61(1081.13-1341.31) | 22.29(19.81-25.29) | -2.10(-2.35 to-1.84) |
| Israel | 4671.90(4357.37-5014.50) | 93.90(87.74-100.69) |  | 4502.75(4101.25-4851.99) | 41.82(38.38-45.09) | -2.09(-2.37 to-1.80) |
| Italy | 17379.21(16878.38-17895.43) | 42.44(41.13-43.82) |  | 10850.76(9928.56-11590.44) | 17.19(15.61-18.85) | -3.31(-3.64 to-2.97) |
| Luxembourg | 399.50(380.69-421.28) | 114.62(107.73-122.15) |  | 413.82(373.40-458.19) | 57.12(50.67-64.92) | -2.08(-2.42 to-1.73) |
| Malta | 138.85(128.04-150.25) | 41.25(37.70-45.04) |  | 181.92(160.38-204.07) | 39.54(33.88-45.58) | -0.22(-0.47 to0.03) |
| Netherlands | 4621.94(4417.77-4835.46) | 34.95(33.19-36.94) |  | 4406.47(4060.35-4674.73) | 21.71(20.04-23.20) | -1.26(-1.46 to-1.06) |
| Norway | 1668.60(1616.86-1715.58) | 37.96(36.78-39.16) |  | 1414.63(1323.59-1483.68) | 20.67(19.58-21.70) | -1.01(-1.50 to-0.52) |
| Portugal | 9071.92(8614.35-9576.49) | 120.75(113.14-129.38) |  | 8013.69(7253.87-8636.74) | 52.92(48.27-57.49) | -2.71(-3.24 to-2.17) |
| Spain | 30564.02(29497.01-31490.49) | 99.83(95.42-103.99) |  | 41779.22(36709.90-45170.28) | 59.76(54.21-64.32) | -1.30(-1.69 to-0.89) |
| Sweden | 4203.60(4032.28-4347.71) | 43.18(41.58-44.78) |  | 2333.61(2105.37-2555.29) | 16.61(15.14-18.00) | -2.68(-3.05 to-2.32) |
| Switzerland | 1958.52(1868.47-2054.65) | 32.94(31.03-34.94) |  | 2177.83(1979.64-2335.53) | 19.60(17.96-21.32) | -1.66(-1.83 to-1.49) |
| United Kingdom | 28799.12(28333.51-29317.61) | 54.47(53.50-55.54) |  | 18636.19(17984.71-19185.71) | 27.65(26.47-28.72) | -1.98(-2.18 to-1.79) |
| Argentina | 120859.84(114756.95-127437.39) | 363.13(344.86-382.78) |  | 35199.35(32685.73-37876.49) | 88.46(79.96-98.02) | -4.61(-5.14 to-4.07) |
| Chile | 83636.93(79014.18-88331.21) | 603.49(572.61-635.84) |  | 10838.06(10239.49-11450.14) | 60.10(55.94-64.14) | -7.33(-8.94 to-5.68) |
| Uruguay | 17512.22(16467.18-18569.42) | 607.66(569.82-646.39) |  | 4131.86(3845.07-4429.81) | 122.00(110.77-134.02) | -5.55(-6.09 to-5.00) |
| Canada | 13979.23(13429.66-14544.91) | 56.21(53.77-58.79) |  | 14825.43(13700.42-15912.73) | 40.77(36.57-45.43) | -0.70(-0.78 to-0.62) |
| United States of America | 169714.93(166268.58-173158.00) | 71.00(69.64-72.47) |  | 271402.71(254222.39-287874.17) | 96.86(89.16-105.16) | 1.59(1.39 to1.78) |
| Antigua and Barbuda | 45.34(40.15-51.32) | 77.34(68.56-87.56) |  | 40.18(37.26-43.07) | 53.32(48.59-58.31) | -1.20(-1.85 to-0.55) |
| Bahamas | 635.38(567.80-715.28) | 260.26(234.57-290.97) |  | 344.75(278.85-427.86) | 105.01(84.27-131.17) | -2.48(-3.17 to-1.79) |
| Barbados | 147.77(134.82-161.22) | 62.06(55.72-68.62) |  | 110.26(86.55-139.38) | 38.35(28.97-49.92) | -1.73(-2.50 to-0.95) |
| Belize | 731.34(649.31-829.20) | 277.87(250.17-311.12) |  | 410.50(361.22-470.22) | 106.65(93.49-122.38) | -3.00(-3.56 to-2.44) |
| Cuba | 11179.13(10710.16-11680.57) | 114.42(109.26-119.89) |  | 5749.07(5051.25-6450.84) | 51.40(44.89-58.08) | -2.81(-3.58 to-2.03) |
| Dominica | 60.94(33.30-75.87) | 79.06(42.75-97.07) |  | 41.20(27.02-61.39) | 83.21(56.28-125.10) | 0.45(-0.18 to1.08) |
| Dominican Republic | 25986.58(15756.45-31263.06) | 277.66(164.94-331.52) |  | 15273.61(8352.14-19686.04) | 144.18(79.54-187.79) | -1.47(-1.92 to-1.01) |
| Grenada | 179.83(155.15-208.36) | 182.63(160.46-208.17) |  | 114.39(100.41-128.00) | 123.63(107.93-139.73) | -1.40(-2.25 to-0.55) |
| Guyana | 2891.13(2474.24-3380.34) | 297.84(259.35-339.39) |  | 1241.34(942.63-1610.19) | 169.30(129.01-219.38) | -1.32(-1.84 to-0.79) |
| Haiti | 43464.76(15252.37-62959.28) | 433.78(178.78-620.12) |  | 34573.22(15702.41-50978.15) | 231.65(112.81-337.49) | -1.81(-2.10 to-1.53) |
| Jamaica | 2518.08(2258.41-2828.08) | 97.39(87.92-108.50) |  | 996.39(781.44-1271.87) | 40.58(31.50-52.37) | -2.43(-3.21 to-1.64) |
| Saint Lucia | 201.22(174.56-228.51) | 140.05(124.73-156.00) |  | 107.16(87.30-131.04) | 68.92(54.84-87.00) | -2.45(-3.18 to-1.71) |
| Saint Vincent and the Grenadines | 164.41(139.56-191.25) | 142.59(123.21-163.79) |  | 118.56(100.66-139.32) | 114.63(95.08-138.63) | -1.07(-1.67 to-0.47) |
| Suriname | 1136.81(620.71-1402.21) | 275.64(150.81-336.60) |  | 635.91(471.52-915.70) | 125.97(91.46-181.08) | -2.24(-2.70 to-1.79) |
| Trinidad and Tobago | 1531.07(1383.41-1687.85) | 132.49(120.53-145.44) |  | 911.71(720.17-1147.24) | 75.12(59.04-96.01) | -2.05(-2.62 to-1.48) |
| Bolivia (Plurinational State of) | 84078.10(56594.55-120213.08) | 913.80(608.67-1296.25) |  | 42734.35(32521.20-65364.84) | 367.97(279.41-564.51) | -3.04(-3.13 to-2.96) |
| Ecuador | 41488.89(38721.52-44531.08) | 359.36(338.88-382.42) |  | 39027.78(32256.88-46778.28) | 230.52(189.55-276.30) | -1.51(-2.03 to-0.99) |
| Peru | 368455.89(243083.51-431300.37) | 1328.88(879.52-1540.92) |  | 123034.52(87590.39-167196.77) | 356.00(251.66-486.04) | -4.57(-5.02 to-4.11) |
| Colombia | 47019.62(42056.88-52040.28) | 121.83(110.33-133.65) |  | 18452.74(15222.24-22259.45) | 41.57(33.50-50.89) | -2.82(-3.60 to-2.03) |
| Costa Rica | 2630.15(2437.80-2837.82) | 79.17(74.40-84.70) |  | 1954.97(1732.81-2169.46) | 45.03(39.49-50.76) | -2.00(-2.22 to-1.79) |
| El Salvador | 22461.44(14390.62-27116.26) | 326.92(201.98-389.15) |  | 6977.63(5275.95-10904.38) | 112.16(84.68-174.91) | -3.42(-3.82 to-3.02) |
| Guatemala | 55154.72(50799.77-59760.87) | 487.83(458.45-519.02) |  | 35538.44(30005.27-41744.79) | 233.59(196.79-275.26) | -2.49(-3.05 to-1.92) |
| Honduras | 37106.84(25115.20-48233.65) | 572.57(366.31-725.60) |  | 24824.00(15784.37-36023.85) | 268.48(162.37-375.31) | -2.54(-2.60 to-2.48) |
| Mexico | 230350.75(215649.03-251050.12) | 226.68(214.33-243.89) |  | 140507.28(120670.17-165618.06) | 123.85(104.70-149.86) | -2.06(-2.70 to-1.41) |
| Nicaragua | 15052.84(9917.61-19016.42) | 253.81(164.52-312.92) |  | 5315.28(3817.85-7726.17) | 83.41(59.72-121.08) | -3.57(-3.65 to-3.48) |
| Panama | 5725.03(5081.53-6465.72) | 216.53(194.02-242.22) |  | 4686.96(3843.81-5617.48) | 118.89(97.07-143.88) | -2.35(-2.63 to-2.07) |
| Venezuela (Bolivarian Republic of) | 70321.77(65742.11-75423.64) | 299.13(281.48-319.08) |  | 57141.83(43682.14-74330.76) | 238.66(182.72-311.44) | -0.48(-0.96 to0.00) |
| Brazil | 162513.32(148933.03-176405.82) | 107.05(98.39-115.76) |  | 181196.32(164966.16-197683.15) | 91.30(81.57-101.46) | -0.45(-0.65 to-0.25) |
| Paraguay | 7217.14(5278.75-9573.17) | 133.69(97.80-174.70) |  | 7892.40(5430.38-11019.45) | 121.62(84.14-170.00) | -0.20(-0.38 to-0.03) |
| Algeria | 32786.38(16564.31-46814.82) | 96.17(49.87-134.62) |  | 15586.60(9337.11-20043.26) | 35.48(21.19-45.47) | -2.99(-3.06 to-2.91) |
| Bahrain | 252.04(131.20-299.50) | 49.41(25.21-58.15) |  | 252.10(159.48-324.25) | 20.04(13.18-25.95) | -2.86(-3.08 to-2.64) |
| Egypt | 68063.01(36082.56-83425.77) | 99.19(51.82-118.16) |  | 23629.71(17244.69-32792.45) | 21.06(15.58-29.22) | -5.30(-5.66 to-4.95) |
| Iran (Islamic Republic of) | 82705.81(43020.79-103923.66) | 110.18(57.75-137.74) |  | 17192.84(11053.68-21881.04) | 22.71(14.62-28.96) | -3.94(-4.54 to-3.34) |
| Iraq | 21456.05(12651.45-28461.42) | 81.69(47.76-106.66) |  | 15933.17(10346.87-21357.07) | 38.89(25.24-51.96) | -2.29(-2.45 to-2.13) |
| Jordan | 4009.31(2785.64-4867.45) | 80.23(55.27-96.43) |  | 4179.41(2982.68-5489.34) | 36.73(26.17-47.83) | -2.80(-3.01 to-2.58) |
| Kuwait | 1328.54(1222.79-1442.84) | 77.58(71.65-83.64) |  | 592.27(494.92-689.96) | 16.76(13.95-19.51) | -4.35(-4.97 to-3.72) |
| Lebanon | 1556.63(921.86-2026.83) | 45.69(27.08-58.51) |  | 1093.24(691.97-1358.01) | 21.40(13.53-27.23) | -2.41(-2.55 to-2.28) |
| Libya | 3412.98(1985.33-4458.79) | 62.06(36.50-79.46) |  | 2893.31(1649.71-3876.03) | 53.79(31.20-72.44) | 0.20(-0.13 to0.54) |
| Morocco | 37229.40(17850.71-53282.95) | 109.12(53.17-154.81) |  | 12721.74(7476.61-17376.68) | 37.56(21.95-51.39) | -3.36(-3.51 to-3.21) |
| Palestine | 1529.25(940.55-2045.23) | 49.34(30.75-64.51) |  | 1213.13(769.41-1567.64) | 22.91(14.41-28.99) | -2.15(-2.32 to-1.97) |
| Oman | 631.34(369.24-868.88) | 25.37(14.60-34.76) |  | 490.14(301.03-619.66) | 11.88(7.32-14.95) | -1.51(-1.86 to-1.16) |
| Qatar | 95.34(49.05-129.19) | 22.80(11.37-30.56) |  | 247.30(160.27-331.77) | 10.91(7.25-14.36) | -1.88(-2.27 to-1.49) |
| Saudi Arabia | 19817.75(11502.23-27690.80) | 98.88(57.60-137.43) |  | 15681.20(9990.57-21686.92) | 41.25(26.61-55.59) | -2.75(-2.89 to-2.61) |
| Syrian Arab Republic | 16975.19(10033.76-22734.50) | 96.52(59.15-125.74) |  | 5693.03(3846.85-8038.80) | 44.59(30.25-62.77) | -2.28(-2.86 to-1.70) |
| Tunisia | 8383.33(4520.97-11565.24) | 83.52(45.15-114.74) |  | 3046.73(1829.34-4162.72) | 29.53(17.28-40.65) | -3.26(-3.40 to-3.12) |
| Türkiye | 30750.21(18710.01-43699.47) | 47.45(29.40-66.02) |  | 15925.53(10122.86-19954.75) | 23.00(14.81-29.54) | -1.83(-2.04 to-1.62) |
| United Arab Emirates | 753.67(479.41-1123.59) | 40.94(25.80-60.62) |  | 1013.30(641.85-1267.54) | 16.21(10.60-19.99) | -2.21(-2.53 to-1.90) |
| Yemen | 33311.13(14285.20-50217.76) | 128.84(59.26-193.84) |  | 22955.87(12214.12-30721.38) | 55.33(29.35-73.19) | -2.81(-2.87 to-2.75) |
| Afghanistan | 30477.84(14310.52-45244.37) | 187.77(92.56-272.38) |  | 37471.76(20973.56-51997.22) | 83.22(48.12-114.07) | -2.37(-2.56 to-2.18) |
| Bangladesh | 3871.38(2989.88-4903.93) | 4.79(3.27-5.89) |  | 3693.45(2124.47-4565.28) | 2.97(1.35-3.66) | -1.84(-1.99 to-1.69) |
| Bhutan | 179.59(27.26-260.66) | 20.72(3.94-29.08) |  | 60.14(17.45-82.77) | 9.74(2.66-13.44) | -2.76(-2.91 to-2.61) |
| India | 462817.79(223644.49-576212.03) | 44.79(21.93-54.78) |  | 280414.08(152941.46-378298.02) | 25.35(13.79-34.31) | -1.72(-1.81 to-1.62) |
| Nepal | 6831.18(1213.74-9762.91) | 22.37(4.83-30.94) |  | 2672.89(692.83-3813.32) | 9.42(2.41-13.23) | -2.74(-2.79 to-2.68) |
| Pakistan | 45651.98(7377.69-61963.90) | 26.39(5.07-35.17) |  | 45431.98(9597.29-59615.69) | 17.36(3.85-22.33) | -0.77(-1.07 to-0.47) |
| Angola | 31897.32(12909.86-46090.40) | 171.75(82.88-242.54) |  | 37687.68(18030.86-52454.50) | 79.30(38.81-109.22) | -2.45(-2.66 to-2.25) |
| Central African Republic | 8014.72(3922.69-11807.40) | 168.87(90.50-244.96) |  | 9042.70(4665.79-13098.72) | 119.38(62.41-170.64) | -1.01(-1.10 to-0.92) |
| Congo | 4636.40(2407.65-6328.67) | 131.91(69.50-175.88) |  | 3854.46(1709.19-5197.14) | 68.50(31.08-91.98) | -2.43(-2.67 to-2.19) |
| Democratic Republic of the Congo | 101720.62(45713.62-147240.90) | 145.81(70.35-207.92) |  | 77344.67(36295.11-117248.01) | 67.79(32.68-100.16) | -2.24(-2.42 to-2.05) |
| Equatorial Guinea | 1053.00(488.38-1512.70) | 141.09(75.07-198.85) |  | 1240.73(465.81-1949.72) | 75.02(29.13-115.58) | -2.40(-2.60 to-2.20) |
| Gabon | 1504.56(701.16-2000.46) | 109.78(52.06-142.25) |  | 1218.45(460.27-1791.48) | 64.55(25.25-93.49) | -1.38(-1.62 to-1.13) |
| Burundi | 8645.18(3953.15-13016.96) | 88.97(44.25-130.10) |  | 6815.00(3042.24-12118.54) | 38.44(18.98-66.82) | -2.47(-2.69 to-2.25) |
| Comoros | 603.99(313.74-853.87) | 78.09(42.27-108.35) |  | 359.96(236.37-529.30) | 47.43(31.99-69.55) | -1.68(-1.81 to-1.56) |
| Djibouti | 351.74(207.49-486.96) | 58.01(36.27-79.17) |  | 434.53(259.78-676.22) | 34.37(21.70-52.56) | -1.82(-2.11 to-1.53) |
| Eritrea | 3437.47(2014.25-4978.61) | 64.27(39.44-90.50) |  | 3433.72(2061.83-5967.32) | 45.30(28.02-75.50) | -1.10(-1.20 to-1.01) |
| Ethiopia | 71920.67(44418.72-98085.13) | 81.80(52.82-110.46) |  | 39774.97(25405.91-71861.21) | 29.50(19.40-51.83) | -3.59(-3.75 to-3.42) |
| Kenya | 15036.65(9772.11-19755.36) | 39.74(26.43-53.73) |  | 12230.47(8219.25-19167.91) | 25.13(18.16-38.90) | -1.08(-1.24 to-0.93) |
| Madagascar | 15920.99(9751.16-20901.64) | 78.08(48.40-101.65) |  | 14629.69(8610.19-21239.18) | 41.72(26.61-58.78) | -1.76(-1.87 to-1.64) |
| Malawi | 21217.90(10949.77-29957.50) | 110.87(59.17-151.87) |  | 10425.70(5173.06-16009.75) | 44.77(24.21-66.17) | -2.81(-2.93 to-2.70) |
| Mauritius | 496.49(471.86-525.40) | 49.09(46.90-51.77) |  | 474.32(432.02-502.03) | 40.55(36.31-43.82) | -1.88(-2.81 to-0.94) |
| Mozambique | 24597.41(15145.93-36828.48) | 104.20(65.40-153.80) |  | 20562.65(13065.54-39076.01) | 48.83(32.33-86.04) | -2.11(-2.24 to-1.98) |
| Rwanda | 11067.03(5890.59-15512.31) | 93.81(51.37-129.73) |  | 5754.61(3227.81-10592.34) | 38.70(23.07-68.86) | -3.55(-3.85 to-3.25) |
| Seychelles | 38.54(21.67-45.26) | 54.85(30.30-63.46) |  | 37.13(24.67-49.29) | 37.34(25.55-49.93) | -1.00(-1.25 to-0.75) |
| Somalia | 10383.36(5802.73-15172.65) | 73.97(43.30-105.81) |  | 16483.09(10219.27-24845.23) | 49.03(32.06-72.63) | -1.11(-1.32 to-0.91) |
| United Republic of Tanzania | 44159.63(26220.32-58564.99) | 93.61(58.55-122.22) |  | 38228.81(21908.64-79038.57) | 48.95(29.75-96.33) | -1.83(-1.93 to-1.73) |
| Uganda | 23314.40(11760.79-34476.46) | 66.99(35.66-95.94) |  | 27574.08(12422.72-47347.11) | 44.75(22.39-73.38) | -1.36(-1.52 to-1.20) |
| Zambia | 12847.45(7710.10-17142.09) | 88.15(54.73-114.58) |  | 9611.40(5459.93-16387.46) | 40.11(24.18-65.36) | -2.57(-2.74 to-2.40) |
| Botswana | 1045.10(722.06-1590.84) | 70.82(48.50-114.00) |  | 1690.52(1177.21-2676.82) | 70.58(49.18-111.00) | -0.05(-0.23 to0.14) |
| Lesotho | 1121.47(813.57-1771.45) | 61.12(44.31-96.54) |  | 2083.98(1376.71-2914.68) | 109.17(72.27-153.15) | 2.50(2.10 to2.89) |
| Namibia | 1075.57(757.35-1500.92) | 66.86(45.90-93.70) |  | 1864.80(1284.97-3341.57) | 75.13(51.98-132.59) | 0.39(0.18 to0.60) |
| South Africa | 52005.59(30638.15-60408.30) | 125.71(73.87-144.84) |  | 50948.09(30839.74-62802.36) | 91.04(55.61-111.16) | -1.16(-1.40 to-0.92) |
| Eswatini | 841.21(617.14-1291.14) | 84.15(61.24-128.37) |  | 1311.17(878.23-2103.36) | 108.69(72.91-173.17) | 1.20(0.71 to1.69) |
| Zimbabwe | 9428.68(4814.89-11810.44) | 79.88(39.60-98.12) |  | 20507.17(11839.11-27783.87) | 125.39(70.41-170.74) | 1.93(1.51 to2.35) |
| Benin | 6873.88(4023.92-9937.34) | 79.11(49.20-110.10) |  | 16764.00(6851.33-24757.98) | 86.76(36.43-126.52) | 0.21(-0.14 to0.57) |
| Burkina Faso | 18517.09(9799.38-25735.12) | 104.05(59.07-142.43) |  | 26750.13(12396.35-51823.35) | 71.33(35.87-133.35) | -0.99(-1.14 to-0.85) |
| Cameroon | 11426.10(6893.05-15510.40) | 67.89(42.27-89.35) |  | 36030.13(13857.56-50026.97) | 91.98(37.36-124.94) | 0.89(0.42 to1.37) |
| Cabo Verde | 21.88(12.77-78.86) | 5.57(3.44-17.02) |  | 252.19(46.89-342.79) | 50.13(9.15-68.07) | 5.94(3.19 to8.76) |
| Chad | 7060.69(4287.83-9983.83) | 65.11(42.08-90.11) |  | 28140.66(13835.52-38299.40) | 97.77(48.25-131.70) | 1.20(0.80 to1.60) |
| Côte d'Ivoire | 12205.45(6733.78-17403.54) | 62.06(35.43-86.76) |  | 28167.40(11155.11-40302.18) | 80.74(33.61-114.62) | 0.69(0.26 to1.12) |
| Gambia | 770.28(472.15-1152.98) | 51.19(32.76-75.07) |  | 1693.17(870.03-2675.43) | 64.48(34.06-94.69) | 0.29(-0.16 to0.74) |
| Ghana | 13081.51(8706.42-18212.41) | 56.88(39.99-79.85) |  | 15639.18(9846.41-25905.99) | 39.42(26.41-63.19) | -0.81(-1.00 to-0.62) |
| Guinea | 10180.74(5635.03-15083.34) | 95.03(55.36-138.37) |  | 17235.33(7266.92-23658.63) | 93.16(40.50-126.04) | -0.03(-0.47 to0.41) |
| Guinea-Bissau | 1499.74(819.91-2254.19) | 94.56(54.55-138.49) |  | 1918.15(942.74-2612.03) | 82.60(41.59-107.63) | -0.61(-1.11 to-0.11) |
| Liberia | 5115.94(2594.98-7468.22) | 117.67(65.40-167.17) |  | 4914.73(1871.96-7462.81) | 77.88(31.45-115.60) | -1.68(-2.02 to-1.35) |
| Mali | 13294.31(7449.12-20651.90) | 87.30(51.79-133.19) |  | 31056.92(13441.35-42538.69) | 86.58(39.79-114.57) | -0.15(-0.55 to0.25) |
| Mauritania | 1425.80(974.76-1991.54) | 48.32(35.09-66.87) |  | 2589.85(1391.52-3590.98) | 52.47(27.95-70.78) | -0.14(-0.67 to0.39) |
| Niger | 15297.77(8409.93-23413.97) | 99.42(57.34-147.98) |  | 27934.58(12018.86-42021.04) | 71.28(31.90-105.39) | -1.55(-1.93 to-1.17) |
| Nigeria | 104664.09(61556.86-149589.78) | 72.92(44.19-100.99) |  | 315575.25(119620.35-460630.07) | 99.10(38.43-140.95) | 1.00(0.65 to1.36) |
| Sao Tome and Principe | 328.10(231.30-463.78) | 181.96(131.45-250.32) |  | 147.74(95.85-259.84) | 73.50(48.82-119.81) | -3.03(-3.27 to-2.79) |
| Senegal | 8600.74(4989.11-11963.81) | 68.81(42.13-92.76) |  | 11292.56(5804.22-17072.11) | 63.49(32.64-93.83) | -0.41(-0.86 to0.05) |
| Sierra Leone | 8192.22(4177.97-12248.22) | 110.11(59.92-162.47) |  | 12017.91(5328.20-17391.72) | 102.78(46.76-147.61) | -0.62(-1.00 to-0.24) |
| Togo | 3284.28(1977.36-4470.08) | 57.27(35.93-75.31) |  | 6288.88(2623.38-9097.68) | 69.12(29.10-97.46) | 0.45(0.08 to0.83) |
| American Samoa | 95.41(64.28-131.24) | 170.86(111.64-235.90) |  | 88.26(56.12-118.06) | 200.02(127.40-266.44) | 0.78(0.65 to0.91) |
| Bermuda | 36.85(34.04-39.89) | 66.90(60.99-73.16) |  | 21.73(18.15-26.02) | 29.73(23.77-36.29) | -2.72(-3.63 to-1.80) |
| Cook Islands | 11.18(7.77-14.36) | 57.20(39.63-72.07) |  | 5.98(4.09-7.72) | 36.24(25.59-47.85) | -2.33(-2.69 to-1.97) |
| Greenland | 138.22(91.26-173.83) | 248.68(166.79-314.50) |  | 64.05(44.98-82.52) | 131.25(93.18-169.63) | -1.83(-2.06 to-1.60) |
| Guam | 57.31(37.58-78.10) | 39.07(25.04-52.40) |  | 74.95(41.75-93.23) | 50.37(28.33-63.33) | 1.61(1.31 to1.90) |
| Monaco | 16.46(10.56-20.59) | 56.12(37.73-73.15) |  | 29.83(19.69-43.27) | 66.64(43.71-96.66) | 0.17(-0.16 to0.50) |
| Nauru | 14.19(8.24-18.53) | 120.87(69.80-159.32) |  | 16.85(9.54-22.60) | 143.08(78.79-191.45) | 0.61(0.11 to1.11) |
| Niue | 2.01(1.30-2.80) | 87.04(55.77-121.85) |  | 3.50(2.28-4.45) | 260.57(172.00-328.86) | 1.69(1.01 to2.37) |
| Northern Mariana Islands | 28.93(19.34-44.71) | 62.34(42.55-96.91) |  | 47.51(25.92-57.64) | 107.39(61.13-132.09) | 2.45(2.09 to2.81) |
| Palau | 25.98(16.12-36.64) | 170.20(105.98-239.74) |  | 24.66(15.08-31.77) | 159.36(101.66-206.09) | 0.04(-0.10 to0.18) |
| Puerto Rico | 3673.45(3502.01-3834.62) | 106.96(101.81-111.74) |  | 2215.05(1855.20-2555.34) | 60.96(51.89-70.31) | -2.23(-3.12 to-1.33) |
| Saint Kitts and Nevis | 41.10(37.62-45.00) | 97.32(89.36-106.03) |  | 24.62(20.18-29.81) | 51.49(41.34-63.54) | -2.04(-2.94 to-1.15) |
| San Marino | 7.46(4.83-9.33) | 41.07(26.81-52.93) |  | 6.05(3.96-8.37) | 15.69(10.56-21.74) | -2.49(-2.68 to-2.31) |
| Tokelau | 1.35(0.85-1.95) | 81.68(51.54-115.92) |  | 2.67(1.72-3.46) | 234.46(151.89-311.82) | 0.77(-0.24 to1.79) |
| Tuvalu | 17.30(8.69-25.66) | 135.82(72.93-196.74) |  | 11.53(6.39-15.02) | 93.24(51.48-121.62) | -1.03(-1.09 to-0.96) |
| United States Virgin Islands | 113.40(60.96-141.51) | 108.71(59.26-134.76) |  | 54.31(32.75-74.69) | 66.04(40.05-93.26) | -1.06(-1.49 to-0.63) |
| South Sudan | 9287.22(5094.51-13292.68) | 92.06(52.02-129.26) |  | 10717.08(5758.78-16508.09) | 71.48(40.05-109.33) | -0.62(-1.10 to-0.14) |
| Sudan | 66844.91(27609.83-102813.26) | 198.01(85.88-303.70) |  | 33439.78(16069.88-46257.18) | 64.49(31.41-88.73) | -3.41(-3.55 to-3.27) |

^a^Disability-adjusted life years, ^b^Uncertainty interval ^c^ , Estimated annual percentage change, ^d^Confidence interval
