# Supplementary material for: Global, regional, and national assessment of foreign body aspiration (1990–2021): novel insights into incidence, mortality, and disability-adjusted life years
Source: Scand J Trauma Resusc Emerg Med. 2025 Mar 11;33:40. doi: 10.1186/s13049-025-01352-z (PMC11895196; doi:10.1186/s13049-025-01352-z)
Supplement: Supplementary file 10 — Supplementary Material 10: Table S5 YLLs and age‑standardized YLLs rate for foreign body aspiration in 1990 and 2021, and temporal trends globally, and in 21 regions and 204 countries. [file 13049_2025_1352_MOESM10_ESM.docx]

| **S5** **Table** YLLs^a^ and age‑standardized YLLs ^a^ rate for foreign body aspiration in 1990 and 2021, and temporal trends | | | | | | |
| --- | --- | --- | --- | --- | --- | --- |
|  | **1990** | |  | **2021** | | **1990-2021 EAPC**^c^**（95%CI**^d^**）** |
|  | **YYLs** ^a^ **number (95%UI**^b^**)** | **Age-standardized YYLs** ^a^ **rate per 100,000 (95% UI**^b^**)** |  | **YYLs** ^a^ **number (95%UI**^b^**)** | **Age-standardized YYLs** ^a^ **rate per 100,000 (95% UI**^b^**)** |  |
| **Global** | 6819050.30(5358395.91-7924634.56) | 116.44(92.42-134.67) |  | 4481114.74(3288799.82-5157241.89) | 62.93(45.77-73.04) | -2.09(-2.18 to-2.00) |
| **Sex** |  |  |  |  |  |  |
| **Males** | 4011666.18(2666610.69 -4851648.36) | 136.44(92.75 -163.11) |  | 2765692.44(1802837.82-3192493.91) | 76.54(49.29-88.94) | -2.02(-2.12 to-1.92) |
| **Females** | 2807384.12(2336423.34 -3107849.74) | 96.48(80.81 -106.57) |  | 1715422.30(1270071.96-1961482.63) | 49.66(36.35-57.57) | -2.19(-2.27 to-2.11) |
| **Socio-demographic index** |  |  |  |  |  |  |
| **High SDI**^e^ | 689347.51(653650.31-711284.53) | 88.08(83.34-91.33) |  | 691457.28(642379.34-727041.03) | 63.79(59.00-67.98) | -0.84(-0.91 to-0.76) |
| **High-middle SDI**^e^ | 1398049.53(1270144.44-1731508.63) | 147.49(133.34-183.88) |  | 777681.85(675932.79-841673.46) | 70.79(60.17-78.09) | -2.95(-3.20 to-2.70) |
| **Middle SDI**^e^ | 2829120.08(2218382.79-3549026.14) | 147.75(115.62-184.28) |  | 1330559.03(950136.67-1526315.33) | 68.42(49.58-79.44) | -2.67(-2.78 to-2.56) |
| **Low-middle SDI**^e^ | 1184895.82(786239.17-1387242.10) | 74.82(49.30-86.69) |  | 831262.94(547009.41-992162.95) | 45.56(30.15-54.15) | -1.47(-1.55 to-1.39) |
| **Low SDI**^e^ | 709786.36(402358.64-925916.33) | 82.91(47.42-107.64) |  | 844993.67(404760.08-1139763.95) | 57.81(28.37-76.52) | -1.08(-1.18 to-0.98) |
| **Region** |  |  |  |  |  |  |
| **East Asia** | 1946886.29(1549486.13-3050498.24) | 170.34(135.66-266.70) |  | 634140.87(394338.97-765176.39) | 76.42(49.95-93.39) | -3.23(-3.50 to-2.96) |
| **Southeast Asia** | 441032.74(267111.13-528452.40) | 83.05(50.49-98.44) |  | 313847.06(199296.22-380086.49) | 53.68(33.91-65.14) | -1.45(-1.50 to-1.40) |
| **Oceania** | 8910.34(5289.86-12937.69) | 105.58(60.82-149.16) |  | 19457.92(10157.44-26668.06) | 114.83(60.93-154.19) | 0.42(0.18 to0.65) |
| **Central Asia** | 162163.39(140412.33-184712.50) | 191.20(167.84-216.94) |  | 118293.63(100345.33-139600.29) | 120.03(101.95-141.49) | -2.02(-2.29 to-1.76) |
| **Central Europe** | 167672.44(160178.35-174883.65) | 157.92(150.17-165.40) |  | 73359.04(66946.43-79973.59) | 61.89(55.78-68.14) | -2.82(-3.13 to-2.50) |
| **Eastern Europe** | 359788.02(346945.62-378650.38) | 175.28(168.10-185.37) |  | 320622.60(296899.39-341290.16) | 150.75(140.95-159.14) | -1.67(-2.39 to-0.93) |
| **High-income Asia Pacific** | 156106.56(127297.49-169022.79) | 114.43(92.66-126.51) |  | 160666.78(140472.77-172961.25) | 54.85(50.70-61.79) | -2.60(-2.73 to-2.47) |
| **Australasia** | 9360.87(8938.43-9761.87) | 52.45(49.88-54.87) |  | 9797.37(9039.88-10452.67) | 36.57(33.25-39.88) | -0.45(-0.88 to-0.01) |
| **Western Europe** | 265602.85(258914.29-271600.23) | 78.73(76.86-80.72) |  | 222631.22(204033.46-233475.01) | 38.70(36.20-40.80) | -2.07(-2.28 to-1.86) |
| **Southern Latin America** | 220488.35(211574.80-229903.89) | 440.08(422.48-458.72) |  | 49219.84(46057.97-52486.35) | 81.52(74.48-89.20) | -5.38(-6.12 to-4.63) |
| **High-income North America** | 180315.85(176520.94-183507.81) | 68.51(67.19-69.78) |  | 282349.70(263318.60-299263.31) | 90.66(83.34-98.49) | 1.48(1.29 to1.67) |
| **Caribbean** | 96884.34(64433.30-119665.06) | 245.12(166.16-300.04) |  | 64305.15(41008.56-83203.83) | 153.40(95.13-200.67) | -1.28(-1.72 to-0.84) |
| **Andean Latin America** | 490499.41(350351.41-576113.65) | 992.16(704.20-1154.04) |  | 202865.10(157034.14-267435.70) | 321.95(247.98-424.99) | -3.86(-4.24 to-3.49) |
| **Central Latin America** | 479298.84(446026.19-511605.53) | 236.84(220.65-251.05) |  | 291125.10(246252.07-350370.91) | 128.32(106.89-156.82) | -1.94(-2.27 to-1.61) |
| **Tropical Latin America** | 166100.87(152394.80-179934.80) | 105.46(97.17-113.98) |  | 186768.82(168710.60-205701.58) | 91.41(81.21-102.41) | -0.40(-0.60 to-0.20) |
| **North Africa and Middle East** | 455371.15(243378.99-575220.99) | 98.42(52.36-122.64) |  | 224471.64(131375.97-279075.95) | 37.38(21.82-46.35) | -2.99(-3.05 to-2.94) |
| **South Asia** | 493326.78(207176.71-612367.11) | 35.04(14.66-42.82) |  | 311090.35(144926.22-408179.86) | 20.79(9.88-27.01) | -1.54(-1.64 to-1.43) |
| **Central Sub-Saharan Africa** | 147315.38(65735.09-203489.65) | 148.03(71.93-201.74) |  | 128226.19(59023.36-179225.57) | 71.51(33.44-99.14) | -2.19(-2.36 to-2.02) |
| **Eastern Sub-Saharan Africa** | 269629.96(162036.85-352733.54) | 78.53(48.77-102.39) |  | 212977.31(121763.58-373283.72) | 38.96(23.57-65.35) | -2.19(-2.25 to-2.13) |
| **Southern Sub-Saharan Africa** | 64672.39(38569.56-75167.73) | 108.84(64.11-125.96) |  | 77606.68(46626.65-96619.35) | 95.58(57.35-118.34) | -0.42(-0.67 to-0.18) |
| **Western Sub-Saharan Africa** | 237623.50(142057.05-330697.79) | 73.12(44.94-99.28) |  | 577292.37(236215.36-786714.64) | 85.85(36.12-116.28) | 0.49(0.14 to0.83) |
| **Country** |  |  |  |  |  |  |
| **China** | 1891670.14(1497431.08-2984994.08) | 171.61(135.93-270.59) |  | 607199.77(368717.06-737524.46) | 76.35(48.98-93.56) | -3.30(-3.58 to-3.01) |
| **Democratic People's Republic of Korea** | 30958.03(20062.35-50374.18) | 127.86(83.74-206.93) |  | 13685.10(8953.54-22497.40) | 73.78(46.45-128.17) | -1.31(-1.63 to-0.98) |
| **Taiwan (Province of China)** | 24258.12(22932.95-25725.28) | 143.57(135.33-152.67) |  | 13255.99(12015.63-14298.96) | 86.22(74.69-97.05) | -1.55(-1.89 to-1.20) |
| **Cambodia** | 17116.40(7042.85-27615.11) | 107.89(55.95-172.12) |  | 10249.20(6907.10-16061.98) | 63.38(42.95-98.61) | -2.11(-2.28 to-1.94) |
| **Indonesia** | 143998.04(32347.38-190454.43) | 68.02(15.50-89.41) |  | 77728.45(20746.95-99459.27) | 36.51(9.22-46.62) | -2.23(-2.34 to-2.11) |
| **Lao People's Democratic Republic** | 9072.35(3047.12-15206.04) | 139.54(57.70-226.14) |  | 5555.69(3530.58-8781.05) | 72.46(46.93-113.01) | -2.20(-2.32 to-2.09) |
| **Malaysia** | 16578.37(9735.81-20950.22) | 90.68(53.01-113.70) |  | 17651.38(12689.22-24500.27) | 62.62(46.65-87.94) | -1.41(-1.70 to-1.12) |
| **Maldives** | 825.46(368.80-1160.98) | 261.45(133.03-351.87) |  | 345.80(253.31-481.24) | 93.11(69.30-127.24) | -3.13(-3.23 to-3.04) |
| **Myanmar** | 63867.42(24405.35-103445.01) | 133.84(56.37-212.90) |  | 40761.51(27402.94-60159.55) | 78.16(52.57-115.36) | -2.00(-2.21 to-1.79) |
| **Philippines** | 50475.71(31867.13-62136.64) | 64.05(39.57-76.49) |  | 51396.22(30565.00-61025.61) | 47.70(29.00-56.35) | -0.51(-0.74 to-0.29) |
| **Sri Lanka** | 20349.86(10681.49-23403.83) | 124.58(66.18-142.90) |  | 10702.45(6885.39-15978.21) | 51.75(34.10-77.26) | -3.64(-4.11 to-3.17) |
| **Thailand** | 25402.84(18598.10-41316.26) | 53.76(39.21-81.52) |  | 43257.98(26186.50-55573.23) | 69.82(45.95-87.93) | 1.19(0.73 to1.65) |
| **Timor-Leste** | 1213.21(468.80-1984.13) | 92.77(42.95-145.20) |  | 955.90(669.77-1471.56) | 57.81(41.54-88.98) | -1.63(-1.91 to-1.34) |
| **Viet Nam** | 90977.91(37873.10-121385.67) | 107.14(47.12-139.79) |  | 54308.90(28495.98-71800.66) | 68.09(33.93-90.79) | -1.39(-1.49 to-1.29) |
| **Fiji** | 1225.08(863.94-1666.26) | 152.90(105.74-206.42) |  | 1479.31(960.97-2002.40) | 165.23(107.32-222.80) | 0.31(0.07 to0.55) |
| **Kiribati** | 17.70(10.82-24.07) | 17.08(10.57-22.97) |  | 16.41(9.97-25.05) | 12.72(7.79-19.08) | -0.83(-0.94 to-0.72) |
| **Marshall Islands** | 42.92(27.48-57.33) | 87.30(54.20-113.73) |  | 59.55(32.29-81.12) | 107.00(58.11-145.86) | 0.78(0.49 to1.07) |
| **Micronesia (Federated States of)** | 116.87(72.67-166.60) | 100.69(61.52-141.24) |  | 87.62(47.23-119.48) | 88.10(47.26-119.81) | -0.41(-0.47 to-0.35) |
| **Papua New Guinea** | 6019.00(3400.85-8917.62) | 105.59(58.57-152.56) |  | 15539.04(7912.46-21836.07) | 114.76(58.81-157.90) | 0.42(0.15 to0.69) |
| **Samoa** | 141.84(98.76-198.95) | 71.11(47.33-103.42) |  | 156.66(93.14-212.08) | 70.20(40.46-95.42) | 0.13(0.07 to0.18) |
| **Solomon Islands** | 342.59(186.78-506.61) | 81.19(40.90-116.39) |  | 629.51(341.46-939.27) | 85.97(45.20-127.74) | 0.29(0.14 to0.45) |
| **Tonga** | 56.59(39.36-78.89) | 48.31(32.57-69.59) |  | 67.22(39.45-93.73) | 58.92(33.74-81.59) | 0.78(0.49 to1.08) |
| **Vanuatu** | 128.63(77.16-182.96) | 70.36(40.70-99.94) |  | 275.53(150.99-377.56) | 83.98(45.30-113.16) | 0.57(0.32 to0.83) |
| **Armenia** | 8640.29(6945.82-10663.05) | 238.72(192.09-294.21) |  | 3353.36(2703.90-4069.56) | 138.34(108.50-173.99) | -2.18(-2.40 to-1.96) |
| **Azerbaijan** | 14093.26(8456.01-20791.83) | 167.01(100.17-244.76) |  | 6424.14(3961.15-10080.24) | 77.92(48.74-123.84) | -2.80(-2.94 to-2.67) |
| **Georgia** | 4956.84(4140.06-5978.46) | 104.95(86.92-126.36) |  | 2690.11(2325.80-3078.29) | 79.21(66.80-92.74) | -1.12(-1.42 to-0.82) |
| **Kazakhstan** | 46527.08(40575.94-52175.38) | 270.83(236.52-302.92) |  | 35948.08(30349.64-42243.87) | 182.12(153.80-213.99) | -2.27(-2.96 to-1.57) |
| **Kyrgyzstan** | 10424.73(9029.99-12118.64) | 182.88(160.50-210.30) |  | 7379.88(6399.86-8471.12) | 102.42(88.79-117.48) | -2.48(-2.93 to-2.02) |
| **Mongolia** | 11652.55(8369.43-17614.93) | 375.93(272.68-585.74) |  | 6520.41(4088.07-8597.61) | 182.38(114.27-241.31) | -2.25(-2.41 to-2.09) |
| **Tajikistan** | 14614.10(6690.38-21150.54) | 170.89(84.20-247.34) |  | 11777.74(7099.81-18372.33) | 94.21(56.93-143.88) | -2.38(-2.66 to-2.11) |
| **Turkmenistan** | 8790.90(7303.11-10499.11) | 165.48(139.84-195.71) |  | 6241.18(5176.56-7481.90) | 117.94(97.87-141.18) | -1.46(-1.70 to-1.21) |
| **Uzbekistan** | 42463.62(33831.64-51997.36) | 138.09(110.99-168.25) |  | 37958.73(29772.49-46976.85) | 102.16(80.34-125.87) | -1.33(-1.55 to-1.10) |
| **Albania** | 3013.57(1901.70-3919.18) | 79.74(50.19-103.31) |  | 776.60(479.33-1092.26) | 39.01(24.21-55.10) | -2.42(-2.55 to-2.29) |
| **Bosnia and Herzegovina** | 794.64(452.36-1011.88) | 18.45(10.67-23.69) |  | 567.88(276.52-763.73) | 17.09(9.21-23.07) | -0.67(-0.91 to-0.43) |
| **Bulgaria** | 11296.17(10371.76-12226.73) | 163.37(150.50-176.67) |  | 4820.25(4015.61-5689.46) | 76.71(63.83-90.26) | -2.86(-3.16 to-2.55) |
| **Croatia** | 2665.19(2515.04-2832.58) | 60.72(56.95-64.99) |  | 1558.61(1352.32-1780.52) | 34.23(29.12-40.06) | -1.84(-2.14 to-1.55) |
| **Czechia** | 15159.18(14341.57-16004.85) | 169.62(158.85-180.87) |  | 6168.91(5309.94-7078.32) | 53.38(45.90-61.75) | -3.53(-3.87 to-3.19) |
| **Hungary** | 15784.68(14777.03-16798.20) | 167.58(155.62-179.90) |  | 7207.78(6179.11-8351.63) | 67.83(57.83-79.35) | -2.98(-3.18 to-2.78) |
| **North Macedonia** | 1171.15(748.46-1462.97) | 67.33(43.27-84.64) |  | 386.91(215.99-490.81) | 18.26(10.83-23.36) | -3.43(-3.74 to-3.12) |
| **Montenegro** | 412.17(255.30-565.13) | 72.81(45.31-99.67) |  | 245.93(156.43-313.96) | 37.76(25.04-49.42) | -2.18(-2.51 to-1.84) |
| **Poland** | 47626.19(46343.68-48915.12) | 140.26(135.83-144.61) |  | 20494.16(18749.46-22249.37) | 50.64(46.37-54.89) | -3.07(-3.42 to-2.72) |
| **Romania** | 52141.71(48634.87-55607.50) | 288.16(266.35-310.46) |  | 21843.89(19199.83-24402.54) | 112.38(99.25-124.91) | -2.58(-3.20 to-1.96) |
| **Serbia** | 6369.25(3815.25-8036.87) | 78.51(46.72-99.86) |  | 2774.67(1698.43-3395.62) | 29.88(19.41-37.25) | -3.26(-3.55 to-2.96) |
| **Slovakia** | 7226.05(4525.16-9628.24) | 146.97(93.49-195.75) |  | 4887.58(3181.16-6172.89) | 92.31(64.43-117.83) | -1.37(-1.54 to-1.20) |
| **Slovenia** | 1331.10(1264.92-1397.14) | 75.02(70.54-79.76) |  | 558.11(475.84-629.90) | 23.84(20.45-26.92) | -3.42(-3.86 to-2.98) |
| **Belarus** | 16760.46(14895.37-19632.66) | 181.67(159.24-210.77) |  | 14436.39(11780.41-17400.50) | 136.04(112.17-162.69) | -1.78(-2.40 to-1.15) |
| **Estonia** | 4575.20(4235.73-4937.87) | 314.12(290.34-339.85) |  | 1474.62(1299.79-1668.49) | 99.36(88.22-111.69) | -4.74(-5.45 to-4.03) |
| **Latvia** | 4005.83(3732.73-4418.63) | 157.54(145.61-172.80) |  | 1976.16(1723.33-2284.22) | 91.16(79.69-104.18) | -3.11(-3.56 to-2.65) |
| **Lithuania** | 7169.83(6680.52-7741.58) | 208.59(193.74-224.91) |  | 4460.80(3960.52-5003.19) | 142.67(128.22-159.12) | -2.16(-2.77 to-1.55) |
| **Republic of Moldova** | 12693.81(11632.36-13959.34) | 301.73(275.88-333.95) |  | 8637.09(7460.36-9859.46) | 245.91(205.75-292.46) | -1.18(-1.59 to-0.76) |
| **Russian Federation** | 231702.36(228648.72-234828.37) | 165.07(162.38-167.82) |  | 246435.99(228136.07-263167.61) | 162.28(152.11-172.44) | -1.42(-2.26 to-0.58) |
| **Ukraine** | 82880.53(72595.35-98792.57) | 184.47(159.67-217.94) |  | 43201.54(31769.66-57053.69) | 116.21(92.57-142.83) | -2.31(-2.89 to-1.72) |
| **Brunei Darussalam** | 185.04(123.78-243.30) | 74.74(49.57-94.74) |  | 184.77(128.02-234.05) | 54.19(38.16-69.85) | -0.43(-0.63 to-0.22) |
| **Japan** | 90215.56(87369.79-92398.03) | 88.54(86.00-91.08) |  | 134035.52(116480.34-143289.85) | 59.85(55.72-63.21) | -1.65(-1.95 to-1.34) |
| **Republic of Korea** | 64752.65(36860.06-76902.71) | 181.70(107.21-216.85) |  | 25687.80(20420.34-37737.71) | 46.94(37.80-72.12) | -4.36(-4.66 to-4.06) |
| **Singapore** | 953.30(896.81-1018.25) | 38.68(36.31-41.39) |  | 758.70(692.76-815.36) | 13.19(11.75-14.59) | -3.56(-3.77 to-3.35) |
| **Australia** | 7296.95(6915.00-7650.22) | 48.91(46.19-51.60) |  | 6615.91(6115.47-7093.31) | 26.87(24.39-29.75) | -1.27(-1.72 to-0.81) |
| **New Zealand** | 2063.92(1934.16-2203.29) | 67.23(62.80-71.93) |  | 3181.46(2877.93-3443.40) | 83.32(73.79-92.13) | 1.59(1.00 to2.19) |
| **Andorra** | 2.48(1.21-3.43) | 6.18(3.14-8.59) |  | 2.44(1.31-3.32) | 2.50(1.37-3.36) | -2.49(-2.69 to-2.30) |
| **Austria** | 2896.44(2777.34-3013.43) | 42.53(40.30-44.88) |  | 2337.93(2155.66-2480.13) | 20.86(19.27-22.35) | -1.62(-1.95 to-1.30) |
| **Belgium** | 8901.28(8501.20-9338.14) | 105.60(100.03-111.91) |  | 12348.77(11193.85-13253.83) | 78.58(72.38-85.21) | -0.57(-0.82 to-0.32) |
| **Cyprus** | 607.14(372.06-797.56) | 90.48(55.23-118.60) |  | 465.69(311.34-604.92) | 32.73(23.01-42.25) | -3.37(-3.51 to-3.24) |
| **Denmark** | 1097.84(1047.33-1156.33) | 23.45(22.03-24.99) |  | 1066.01(986.60-1151.58) | 14.37(13.14-15.73) | -1.67(-1.89 to-1.44) |
| **Finland** | 2263.01(2173.97-2353.99) | 45.79(43.53-47.92) |  | 2303.38(2106.45-2442.78) | 31.84(29.23-34.17) | -1.21(-1.44 to-0.98) |
| **France** | 101243.95(97091.71-105147.18) | 176.78(169.54-183.93) |  | 76757.74(69359.69-81387.08) | 84.93(78.40-90.33) | -2.41(-2.70 to-2.11) |
| **Germany** | 41988.57(40502.03-43734.50) | 67.81(64.67-71.49) |  | 30137.19(28137.31-31851.13) | 24.95(23.55-26.33) | -2.41(-2.72 to-2.10) |
| **Greece** | 4851.52(4627.40-5036.59) | 58.64(55.17-61.92) |  | 4199.08(3879.37-4460.63) | 32.50(29.80-35.28) | -2.00(-2.43 to-1.56) |
| **Iceland** | 77.22(72.07-82.85) | 30.72(28.53-33.14) |  | 75.24(67.71-83.32) | 18.73(16.74-20.94) | -1.14(-1.33 to-0.94) |
| **Ireland** | 1560.51(1498.39-1629.61) | 46.90(44.75-49.29) |  | 1143.78(1027.52-1284.67) | 21.18(18.76-24.10) | -2.14(-2.40 to-1.88) |
| **Israel** | 4567.56(4260.68-4912.07) | 91.78(85.72-98.52) |  | 4399.89(3992.79-4743.68) | 40.76(37.24-44.03) | -2.09(-2.37 to-1.80) |
| **Italy** | 16407.66(15947.95-16844.28) | 40.78(39.48-42.03) |  | 9643.57(8767.26-10261.65) | 15.17(13.65-16.67) | -3.64(-3.97 to-3.31) |
| **Luxembourg** | 392.48(373.85-414.45) | 112.83(106.05-120.39) |  | 406.65(366.05-450.75) | 56.09(49.49-63.91) | -2.09(-2.43 to-1.74) |
| **Malta** | 133.41(122.19-144.59) | 39.82(36.24-43.59) |  | 177.33(155.67-199.56) | 38.57(32.95-44.70) | -0.19(-0.45 to0.07) |
| **Netherlands** | 4314.02(4126.83-4507.98) | 32.95(31.26-34.85) |  | 4154.63(3809.40-4407.33) | 20.34(18.59-21.85) | -1.22(-1.42 to-1.01) |
| **Norway** | 1603.85(1556.18-1649.54) | 36.48(35.34-37.64) |  | 1363.56(1272.34-1430.83) | 19.79(18.71-20.81) | -0.99(-1.50 to-0.48) |
| **Portugal** | 8839.43(8387.42-9340.26) | 118.46(110.91-126.78) |  | 7896.12(7143.61-8521.23) | 51.88(47.32-56.35) | -2.70(-3.25 to-2.15) |
| **Spain** | 29867.74(28786.64-30773.00) | 98.07(93.76-102.20) |  | 41249.48(36126.12-44634.20) | 58.72(53.38-63.20) | -1.29(-1.70 to-0.88) |
| **Sweden** | 4063.66(3901.06-4200.83) | 41.61(40.08-43.12) |  | 2232.34(1994.57-2452.44) | 15.69(14.20-17.07) | -2.74(-3.13 to-2.35) |
| **Switzerland** | 1867.94(1780.67-1953.13) | 31.67(29.82-33.56) |  | 2086.68(1893.56-2240.99) | 18.64(16.95-20.49) | -1.69(-1.86 to-1.51) |
| **United Kingdom** | 27813.80(27419.14-28205.61) | 52.79(51.93-53.68) |  | 17952.64(17266.22-18472.93) | 26.70(25.51-27.72) | -1.99(-2.19 to-1.79) |
| **Argentina** | 119810.15(113651.43-126376.54) | 359.95(341.53-379.78) |  | 34491.08(31976.06-37125.96) | 86.95(78.51-96.48) | -4.64(-5.17 to-4.10) |
| **Chile** | 83246.08(78650.38-87912.18) | 600.55(569.77-632.56) |  | 10645.37(10044.25-11262.71) | 59.10(55.04-63.15) | -7.36(-8.99 to-5.71) |
| **Uruguay** | 17421.64(16384.92-18473.76) | 604.77(567.13-643.23) |  | 4080.67(3787.03-4376.24) | 120.55(109.31-132.64) | -5.58(-6.12 to-5.03) |
| **Canada** | 13660.41(13145.53-14216.32) | 55.11(52.76-57.65) |  | 14459.52(13342.42-15529.74) | 39.99(35.82-44.66) | -0.70(-0.78 to-0.61) |
| **United States of America** | 166513.99(162937.98-169707.01) | 69.81(68.37-71.16) |  | 267822.28(249739.48-284144.63) | 95.94(88.14-104.21) | 1.62(1.42 to1.82) |
| **Antigua and Barbuda** | 43.99(38.86-49.99) | 75.08(66.38-85.12) |  | 39.06(36.07-42.00) | 52.14(47.35-57.22) | -1.18(-1.85 to-0.51) |
| **Bahamas** | 627.36(560.70-707.70) | 257.09(231.71-287.95) |  | 337.85(272.65-420.47) | 103.30(82.57-129.13) | -2.49(-3.19 to-1.79) |
| **Barbados** | 141.82(129.49-155.65) | 59.75(53.74-66.48) |  | 106.29(82.11-136.10) | 37.12(27.66-48.54) | -1.72(-2.53 to-0.91) |
| **Belize** | 725.03(643.38-822.22) | 274.25(247.05-307.15) |  | 402.90(352.50-462.77) | 104.85(91.50-120.52) | -3.01(-3.57 to-2.44) |
| **Cuba** | 10831.55(10356.71-11341.22) | 111.25(106.01-116.82) |  | 5575.02(4877.63-6296.58) | 50.00(43.40-56.51) | -2.82(-3.62 to-2.01) |
| **Dominica** | 59.02(31.54-73.87) | 76.33(40.09-94.36) |  | 40.07(25.88-60.31) | 81.60(54.48-123.50) | 0.50(-0.14 to1.15) |
| **Dominican Republic** | 25777.02(15526.83-31051.64) | 274.60(161.58-328.57) |  | 15117.18(8184.49-19568.15) | 142.77(78.03-186.72) | -1.46(-1.92 to-1.00) |
| **Grenada** | 177.07(152.50-205.56) | 179.31(157.03-205.03) |  | 112.50(98.59-126.41) | 121.86(106.33-138.02) | -1.39(-2.25 to-0.53) |
| **Guyana** | 2859.09(2444.96-3345.82) | 293.61(255.02-334.86) |  | 1224.16(923.34-1592.54) | 167.05(126.48-217.07) | -1.31(-1.84 to-0.77) |
| **Haiti** | 43175.18(14928.02-62707.77) | 429.13(174.23-616.07) |  | 34241.06(15364.89-50684.39) | 229.05(110.16-335.18) | -1.81(-2.10 to-1.52) |
| **Jamaica** | 2447.00(2189.50-2767.28) | 94.30(85.05-105.63) |  | 933.46(717.92-1214.32) | 38.38(29.18-50.25) | -2.50(-3.33 to-1.67) |
| **Saint Lucia** | 197.68(170.84-224.66) | 137.32(121.65-153.05) |  | 104.58(84.84-128.92) | 67.56(53.29-85.78) | -2.45(-3.20 to-1.70) |
| **Saint Vincent and the Grenadines** | 161.32(136.72-188.37) | 139.68(120.07-161.23) |  | 116.49(98.31-137.60) | 112.88(93.26-136.97) | -1.06(-1.67 to-0.44) |
| **Suriname** | 1122.46(604.83-1387.83) | 271.84(146.61-333.05) |  | 623.47(458.98-904.87) | 123.87(89.48-179.24) | -2.25(-2.70 to-1.79) |
| **Trinidad and Tobago** | 1497.04(1355.09-1656.22) | 129.64(118.04-142.87) |  | 890.27(695.68-1125.84) | 73.63(57.50-94.85) | -2.05(-2.63 to-1.46) |
| **Bolivia (Plurinational State of)** | 83534.43(56091.58-119521.90) | 904.91(601.05-1286.80) |  | 42360.43(32229.36-65044.14) | 364.77(276.92-561.77) | -3.04(-3.12 to-2.95) |
| **Ecuador** | 41029.65(38241.38-44108.58) | 354.43(333.84-377.95) |  | 38623.91(31855.54-46452.00) | 228.28(187.80-274.39) | -1.50(-2.02 to-0.98) |
| **Peru** | 365935.33(240380.75-428764.82) | 1316.81(869.16-1528.45) |  | 121880.76(86224.70-165965.57) | 352.84(248.16-482.67) | -4.56(-5.02 to-4.10) |
| **Colombia** | 46054.68(41219.84-51098.32) | 118.78(107.63-130.79) |  | 17723.47(14441.33-21544.37) | 40.12(32.10-49.48) | -2.83(-3.63 to-2.03) |
| **Costa Rica** | 2544.83(2353.49-2740.90) | 76.22(71.46-81.47) |  | 1890.81(1671.10-2106.18) | 43.74(38.36-49.52) | -1.99(-2.22 to-1.76) |
| **El Salvador** | 22166.95(14113.79-26842.62) | 321.07(195.99-384.00) |  | 6899.68(5189.59-10818.95) | 110.95(83.40-173.82) | -3.39(-3.79 to-2.99) |
| **Guatemala** | 54557.60(50222.67-59239.11) | 480.10(451.26-511.54) |  | 35136.89(29541.40-41371.98) | 230.97(193.64-273.10) | -2.47(-3.04 to-1.90) |
| **Honduras** | 36959.28(24979.60-48102.11) | 569.24(362.83-722.40) |  | 24651.19(15616.49-35863.69) | 266.74(160.80-373.46) | -2.54(-2.60 to-2.48) |
| **Mexico** | 227125.88(212140.66-248109.73) | 222.77(210.07-240.27) |  | 138509.29(118413.37-163289.86) | 122.33(102.90-148.25) | -2.04(-2.69 to-1.39) |
| **Nicaragua** | 14885.51(9741.54-18867.23) | 249.23(159.72-309.25) |  | 5194.56(3706.30-7612.76) | 81.57(58.02-119.37) | -3.57(-3.66 to-3.49) |
| **Panama** | 5614.85(4970.31-6366.58) | 211.80(189.34-238.14) |  | 4618.28(3769.87-5557.50) | 117.31(95.33-142.50) | -2.33(-2.62 to-2.05) |
| **Venezuela (Bolivarian Republic of)** | 69389.29(64884.67-74521.95) | 294.11(276.83-313.14) |  | 56500.93(43048.87-73604.56) | 236.28(180.33-308.74) | -0.46(-0.94 to0.03) |
| **Brazil** | 158985.46(145063.47-172768.98) | 104.61(95.75-113.23) |  | 178972.34(162141.87-195494.44) | 90.34(80.51-100.52) | -0.41(-0.61 to-0.21) |
| **Paraguay** | 7115.40(5170.73-9488.34) | 130.97(94.85-172.41) |  | 7796.48(5318.71-10923.37) | 120.27(82.57-168.64) | -0.17(-0.35 to0.00) |
| **Algeria** | 32250.96(15969.67-46262.85) | 93.91(47.75-132.32) |  | 15049.97(8714.86-19533.58) | 34.26(19.78-44.28) | -3.01(-3.09 to-2.94) |
| **Bahrain** | 241.30(120.49-287.44) | 47.28(23.12-55.74) |  | 235.58(143.95-305.68) | 19.02(12.22-24.86) | -2.89(-3.13 to-2.66) |
| **Egypt** | 66896.50(35062.35-82408.36) | 96.92(49.36-116.06) |  | 22503.91(16045.57-31676.72) | 19.92(14.38-28.18) | -5.42(-5.78 to-5.05) |
| **Iran (Islamic Republic of)** | 81454.49(41659.69-102751.95) | 107.84(55.19-135.32) |  | 16386.56(10200.15-21044.83) | 21.79(13.68-28.00) | -3.97(-4.58 to-3.35) |
| **Iraq** | 21080.92(12212.64-28068.99) | 79.46(45.36-104.35) |  | 15501.94(9913.70-20942.28) | 37.82(24.03-50.92) | -2.28(-2.44 to-2.12) |
| **Jordan** | 3926.93(2697.64-4779.67) | 77.82(52.75-93.90) |  | 4036.74(2827.09-5329.18) | 35.56(24.94-46.69) | -2.81(-3.02 to-2.59) |
| **Kuwait** | 1298.60(1192.60-1412.36) | 75.78(69.83-82.05) |  | 546.92(453.51-645.51) | 15.84(13.09-18.58) | -4.43(-5.08 to-3.79) |
| **Lebanon** | 1484.69(825.31-1960.80) | 43.21(23.77-56.14) |  | 1022.36(617.86-1274.52) | 20.16(12.20-25.82) | -2.42(-2.56 to-2.28) |
| **Libya** | 3338.99(1910.84-4376.30) | 60.16(34.47-77.31) |  | 2812.41(1554.95-3798.89) | 52.65(29.87-71.27) | 0.25(-0.09 to0.60) |
| **Morocco** | 36587.81(17223.15-52607.12) | 106.48(50.65-152.02) |  | 12209.80(6985.68-16906.33) | 36.21(20.64-50.01) | -3.39(-3.55 to-3.24) |
| **Palestine** | 1492.30(895.97-2013.80) | 47.26(28.47-62.69) |  | 1155.81(712.09-1509.35) | 21.72(13.19-27.83) | -2.17(-2.35 to-1.99) |
| **Oman** | 602.67(339.75-844.50) | 23.81(12.87-33.31) |  | 452.27(261.51-584.17) | 11.08(6.43-14.08) | -1.48(-1.86 to-1.10) |
| **Qatar** | 85.70(38.97-119.42) | 20.66(9.11-28.72) |  | 221.60(133.01-306.15) | 10.10(6.41-13.49) | -1.76(-2.18 to-1.33) |
| **Saudi Arabia** | 19540.49(11268.13-27404.22) | 97.02(56.03-135.75) |  | 15268.34(9654.12-21227.06) | 40.21(25.55-54.54) | -2.77(-2.91 to-2.63) |
| **Syrian Arab Republic** | 16666.29(9701.22-22367.91) | 93.82(56.14-122.74) |  | 5519.09(3674.47-7882.72) | 43.37(29.01-61.67) | -2.26(-2.86 to-1.66) |
| **Tunisia** | 8209.67(4356.61-11349.82) | 81.34(43.01-111.91) |  | 2913.68(1693.62-4031.22) | 28.45(16.26-39.42) | -3.29(-3.44 to-3.14) |
| **Türkiye** | 29660.78(17385.06-42600.31) | 45.44(27.13-64.02) |  | 15127.65(9407.88-19267.78) | 22.10(13.96-28.75) | -1.80(-2.02 to-1.59) |
| **United Arab Emirates** | 715.18(436.10-1088.58) | 38.90(23.69-58.89) |  | 889.86(506.86-1128.39) | 15.04(9.34-18.99) | -2.24(-2.58 to-1.91) |
| **Yemen** | 33017.12(13970.07-49899.42) | 126.43(56.68-191.37) |  | 22492.25(11766.98-30262.83) | 53.85(27.85-71.80) | -2.83(-2.90 to-2.77) |
| **Afghanistan** | 30232.38(14045.71-45012.75) | 185.13(89.58-269.33) |  | 37031.52(20456.22-51574.13) | 81.66(46.45-112.43) | -2.37(-2.57 to-2.18) |
| **Bangladesh** | 1107.76(702.67-1346.10) | 2.10(0.39-2.63) |  | 1942.86(262.46-2505.76) | 1.88(0.20-2.43) | -0.57(-0.80 to-0.33) |
| **Bhutan** | 165.98(13.61-247.80) | 18.43(1.64-26.83) |  | 52.49(9.74-74.08) | 8.74(1.65-12.39) | -2.75(-2.91 to-2.60) |
| **India** | 442032.99(200671.20-554067.44) | 42.26(19.15-52.01) |  | 263898.52(136723.35-360372.97) | 24.19(12.64-33.06) | -1.68(-1.78 to-1.58) |
| **Nepal** | 6418.01(776.77-9327.50) | 20.10(2.52-28.44) |  | 2340.89(369.77-3462.13) | 8.33(1.33-12.06) | -2.77(-2.83 to-2.72) |
| **Pakistan** | 43602.04(5114.50-59830.06) | 24.39(3.00-33.01) |  | 42855.57(6920.10-57140.99) | 16.22(2.69-21.19) | -0.71(-1.05 to-0.38) |
| **Angola** | 31606.35(12657.56-45712.98) | 168.87(80.14-239.59) |  | 37317.44(17668.07-52021.06) | 78.10(37.47-108.07) | -2.44(-2.65 to-2.24) |
| **Central African Republic** | 7940.70(3851.29-11736.89) | 166.15(87.74-242.52) |  | 8911.65(4507.88-12978.84) | 117.01(59.62-168.60) | -1.02(-1.12 to-0.93) |
| **Congo** | 4575.54(2348.59-6249.46) | 129.38(66.35-172.93) |  | 3788.12(1643.37-5124.26) | 67.26(29.88-90.79) | -2.43(-2.67 to-2.19) |
| **Democratic Republic of the Congo** | 100671.60(44761.77-146375.34) | 143.00(67.47-204.85) |  | 75784.49(34780.48-115663.50) | 65.99(30.67-98.46) | -2.25(-2.44 to-2.06) |
| **Equatorial Guinea** | 1039.10(472.16-1494.26) | 137.74(71.61-194.90) |  | 1227.91(453.72-1937.86) | 74.17(28.31-114.91) | -2.36(-2.56 to-2.16) |
| **Gabon** | 1482.09(679.08-1976.04) | 107.46(49.86-139.89) |  | 1196.59(433.63-1770.58) | 63.34(23.78-92.21) | -1.37(-1.61 to-1.12) |
| **Burundi** | 8557.13(3864.47-12907.18) | 87.35(42.53-128.57) |  | 6664.27(2870.69-11967.01) | 37.26(17.67-65.31) | -2.51(-2.73 to-2.28) |
| **Comoros** | 595.95(306.32-845.53) | 76.29(40.46-106.52) |  | 351.24(227.05-520.47) | 46.25(30.91-68.32) | -1.69(-1.82 to-1.56) |
| **Djibouti** | 345.56(199.55-480.85) | 56.48(34.44-77.95) |  | 423.34(247.96-665.81) | 33.48(20.73-51.76) | -1.82(-2.12 to-1.53) |
| **Eritrea** | 3385.14(1953.41-4929.85) | 62.70(37.99-89.13) |  | 3365.46(1996.15-5903.82) | 44.24(26.83-74.36) | -1.10(-1.20 to-1.01) |
| **Ethiopia** | 70829.36(43328.31-96640.36) | 79.59(50.55-107.59) |  | 38734.47(24319.26-70858.63) | 28.51(18.38-50.83) | -3.62(-3.81 to-3.44) |
| **Kenya** | 14632.66(9348.26-19346.47) | 37.92(24.73-51.44) |  | 11753.37(7784.11-18725.83) | 24.17(17.17-37.92) | -1.07(-1.22 to-0.91) |
| **Madagascar** | 15726.71(9556.79-20710.05) | 76.40(46.68-100.13) |  | 14320.41(8249.33-20900.39) | 40.61(25.41-57.45) | -1.77(-1.89 to-1.65) |
| **Malawi** | 21069.89(10794.07-29781.65) | 109.32(57.48-150.21) |  | 10238.39(4964.35-15808.04) | 43.78(23.15-65.08) | -2.84(-2.96 to-2.72) |
| **Mauritius** | 479.61(455.75-507.72) | 47.54(45.41-50.07) |  | 459.50(416.67-486.95) | 39.45(35.10-42.70) | -1.89(-2.84 to-0.94) |
| **Mozambique** | 24367.26(14927.88-36568.27) | 102.42(63.86-151.32) |  | 20222.69(12703.02-38690.09) | 47.71(31.17-84.91) | -2.13(-2.26 to-1.99) |
| **Rwanda** | 10945.50(5778.63-15390.60) | 92.08(49.73-127.77) |  | 5626.17(3100.02-10457.86) | 37.71(22.16-67.82) | -3.58(-3.89 to-3.28) |
| **Seychelles** | 37.56(20.51-44.32) | 53.46(28.66-62.10) |  | 36.32(23.87-48.59) | 36.60(24.82-49.30) | -0.98(-1.24 to-0.72) |
| **Somalia** | 10251.76(5688.06-15038.57) | 72.27(41.86-104.04) |  | 16234.75(9971.64-24511.54) | 47.81(30.86-71.50) | -1.11(-1.33 to-0.90) |
| **United Republic of Tanzania** | 43732.74(25738.23-58128.88) | 91.91(56.44-120.57) |  | 37615.65(21166.55-78490.03) | 47.87(28.54-95.36) | -1.84(-1.94 to-1.74) |
| **Uganda** | 23062.32(11481.82-34170.51) | 65.50(33.91-94.31) |  | 27180.69(12014.31-46852.45) | 43.80(21.31-72.23) | -1.36(-1.53 to-1.19) |
| **Zambia** | 12730.12(7594.49-17015.60) | 86.66(53.38-113.24) |  | 9438.11(5293.43-16228.48) | 39.21(23.29-64.50) | -2.59(-2.76 to-2.42) |
| **Botswana** | 1026.76(704.46-1574.15) | 69.33(46.92-112.58) |  | 1668.00(1154.80-2652.51) | 69.65(48.31-110.09) | -0.03(-0.21 to0.16) |
| **Lesotho** | 1097.39(788.71-1748.92) | 59.44(42.68-94.78) |  | 2058.24(1346.89-2891.88) | 107.80(70.68-151.94) | 2.55(2.15 to2.95) |
| **Namibia** | 1055.24(732.61-1479.92) | 65.29(44.17-92.18) |  | 1839.53(1256.65-3317.29) | 74.07(50.87-131.50) | 0.42(0.20 to0.63) |
| **South Africa** | 51376.34(30026.12-59853.16) | 123.95(72.17-143.20) |  | 50449.37(30382.50-62314.69) | 90.17(54.82-110.31) | -1.14(-1.38 to-0.90) |
| **Eswatini** | 830.44(605.32-1281.70) | 82.69(59.58-126.85) |  | 1297.83(863.60-2088.51) | 107.53(71.75-171.89) | 1.22(0.72 to1.72) |
| **Zimbabwe** | 9286.22(4677.47-11692.17) | 78.37(38.13-96.50) |  | 20293.71(11633.01-27588.11) | 123.92(69.00-169.39) | 1.95(1.52 to2.38) |
| **Benin** | 6770.43(3904.72-9834.98) | 76.77(46.33-107.38) |  | 16581.10(6648.33-24602.84) | 85.21(34.77-124.80) | 0.25(-0.11 to0.61) |
| **Burkina Faso** | 18332.27(9599.44-25557.79) | 101.96(56.71-139.87) |  | 26422.52(12052.04-51485.96) | 69.78(34.23-131.71) | -1.00(-1.15 to-0.85) |
| **Cameroon** | 11215.35(6662.14-15309.55) | 65.70(40.10-87.35) |  | 35525.36(13367.59-49519.19) | 90.23(35.48-122.81) | 0.92(0.44 to1.41) |
| **Cabo Verde** | 10.75(3.50-68.79) | 2.21(0.78-14.02) |  | 242.34(36.13-332.61) | 48.37(7.22-66.42) | 7.86(4.36 to11.48) |
| **Chad** | 6937.13(4138.21-9877.70) | 62.86(40.04-87.91) |  | 27848.85(13513.50-38039.38) | 95.88(46.19-129.53) | 1.24(0.83 to1.65) |
| **Côte d'Ivoire** | 11959.77(6492.40-17138.87) | 59.91(33.37-84.35) |  | 27755.83(10708.22-39877.55) | 79.12(31.63-113.03) | 0.72(0.28 to1.16) |
| **Gambia** | 750.50(447.45-1132.33) | 49.00(30.32-72.78) |  | 1659.15(834.66-2645.73) | 62.89(32.22-93.26) | 0.33(-0.14 to0.79) |
| **Ghana** | 12832.28(8429.40-17986.66) | 55.10(38.34-78.35) |  | 15297.77(9521.86-25565.10) | 38.37(25.40-62.19) | -0.80(-0.99 to-0.60) |
| **Guinea** | 10045.44(5483.87-14961.91) | 92.61(52.57-135.80) |  | 17014.96(7030.39-23436.49) | 91.32(38.60-124.38) | -0.02(-0.46 to0.43) |
| **Guinea-Bissau** | 1476.07(798.85-2228.68) | 92.07(52.08-136.00) |  | 1888.84(911.79-2586.23) | 81.02(39.91-105.64) | -0.59(-1.10 to-0.08) |
| **Liberia** | 5055.28(2536.37-7401.05) | 115.03(62.36-164.47) |  | 4836.10(1786.74-7391.48) | 76.27(29.60-114.12) | -1.68(-2.02 to-1.34) |
| **Mali** | 13082.61(7245.13-20454.77) | 84.64(48.91-130.50) |  | 30624.83(13021.66-42114.05) | 84.58(37.85-112.34) | -0.14(-0.55 to0.27) |
| **Mauritania** | 1385.03(935.94-1950.71) | 46.16(32.89-64.69) |  | 2535.77(1336.62-3534.05) | 51.11(26.55-69.48) | -0.10(-0.64 to0.44) |
| **Niger** | 15104.36(8200.64-23230.69) | 96.76(55.02-145.69) |  | 27541.70(11624.12-41637.99) | 69.42(30.07-103.56) | -1.56(-1.95 to-1.17) |
| **Nigeria** | 102594.05(59360.64-146973.50) | 70.46(41.67-97.97) |  | 312253.98(116054.14-457604.43) | 97.55(36.69-139.20) | 1.06(0.69 to1.42) |
| **Sao Tome and Principe** | 324.38(226.94-460.49) | 178.68(128.17-247.14) |  | 143.51(92.36-256.03) | 71.42(46.64-117.43) | -3.06(-3.31 to-2.82) |
| **Senegal** | 8437.75(4833.19-11780.10) | 66.47(40.01-90.00) |  | 11076.40(5550.62-16875.47) | 61.99(31.09-92.42) | -0.39(-0.85 to0.08) |
| **Sierra Leone** | 8093.91(4083.98-12153.44) | 107.59(57.28-160.04) |  | 11864.68(5155.03-17256.64) | 100.90(44.66-146.06) | -0.61(-1.00 to-0.23) |
| **Togo** | 3208.30(1890.99-4387.92) | 55.00(33.25-72.80) |  | 6171.93(2503.38-8979.85) | 67.57(27.61-96.08) | 0.50(0.11 to0.89) |
| **American Samoa** | 94.82(63.67-130.73) | 169.52(110.23-234.67) |  | 87.71(55.66-117.52) | 198.92(126.49-265.31) | 0.79(0.66 to0.92) |
| **Bermuda** | 35.40(32.61-38.35) | 64.54(58.65-70.81) |  | 20.93(17.37-25.27) | 28.60(22.57-35.09) | -2.74(-3.69 to-1.78) |
| **Cook Islands** | 10.96(7.50-14.15) | 55.95(38.27-70.87) |  | 5.85(3.96-7.57) | 35.56(24.81-47.15) | -2.35(-2.72 to-1.98) |
| **Greenland** | 137.31(90.37-172.92) | 246.95(165.05-312.55) |  | 63.47(44.28-82.05) | 130.24(92.00-168.83) | -1.83(-2.06 to-1.59) |
| **Guam** | 56.04(36.37-76.93) | 38.11(24.13-51.52) |  | 73.92(40.92-92.22) | 49.75(27.79-62.77) | 1.65(1.35 to1.95) |
| **Monaco** | 15.96(9.99-20.03) | 54.54(36.47-71.67) |  | 29.38(19.22-42.85) | 65.50(42.76-95.62) | 0.20(-0.14 to0.54) |
| **Nauru** | 14.11(8.14-18.44) | 120.04(68.94-158.40) |  | 16.79(9.48-22.55) | 142.48(78.29-190.90) | 0.62(0.12 to1.12) |
| **Niue** | 1.99(1.28-2.77) | 85.95(54.57-120.73) |  | 3.49(2.27-4.44) | 259.90(171.29-328.16) | 1.71(1.03 to2.40) |
| **Northern Mariana Islands** | 28.47(18.85-44.26) | 61.30(41.48-95.83) |  | 47.09(25.43-57.25) | 106.56(60.16-131.25) | 2.48(2.12 to2.84) |
| **Palau** | 25.79(15.94-36.46) | 168.94(104.78-238.52) |  | 24.51(14.91-31.56) | 158.54(100.79-205.20) | 0.05(-0.09 to0.19) |
| **Puerto Rico** | 3575.22(3405.63-3739.73) | 104.25(99.08-109.26) |  | 2166.82(1803.65-2504.67) | 59.68(50.71-69.20) | -2.22(-3.14 to-1.30) |
| **Saint Kitts and Nevis** | 40.19(36.63-44.03) | 95.08(87.02-103.94) |  | 23.94(19.54-29.19) | 50.40(40.23-62.47) | -2.04(-2.96 to-1.12) |
| **San Marino** | 7.12(4.51-8.99) | 39.67(25.37-51.39) |  | 5.69(3.58-8.06) | 14.65(9.45-20.53) | -2.57(-2.77 to-2.37) |
| **Tokelau** | 1.33(0.83-1.93) | 80.15(50.08-114.50) |  | 2.65(1.71-3.45) | 233.64(151.12-310.89) | 0.80(-0.22 to1.82) |
| **Tuvalu** | 17.18(8.55-25.52) | 134.59(71.62-195.37) |  | 11.45(6.30-14.94) | 92.57(50.85-120.95) | -1.02(-1.09 to-0.95) |
| **United States Virgin Islands** | 110.27(57.63-138.26) | 105.73(56.06-131.90) |  | 52.94(31.28-73.39) | 64.59(38.68-91.59) | -1.04(-1.48 to-0.60) |
| **South Sudan** | 9205.15(5030.91-13207.00) | 90.62(50.76-127.79) |  | 10622.90(5674.36-16414.86) | 70.46(38.98-108.12) | -0.61(-1.11 to-0.12) |
| **Sudan** | 66338.27(27195.66-102306.71) | 195.31(82.97-301.12) |  | 32884.00(15526.94-45721.09) | 63.14(30.10-87.37) | -3.42(-3.57 to-3.28) |

^a^Years of life lost, ^b^Uncertainty interval,  ^c^Estimated annual percentage change, ^d^Confidence interval, ^e^Sociodemographic indices,
