# Supplementary material for: Global, regional, and national assessment of foreign body aspiration (1990–2021): novel insights into incidence, mortality, and disability-adjusted life years
Source: Scand J Trauma Resusc Emerg Med. 2025 Mar 11;33:40. doi: 10.1186/s13049-025-01352-z (PMC11895196; doi:10.1186/s13049-025-01352-z)
Supplement: Supplementary file 11 — Supplementary Material 11: Table S6 YLDs and age‑standardized YLDs rate for foreign body aspiration in 1990 and 2021, and temporal trends globally, and in 21 regions and 204 countries. [file 13049_2025_1352_MOESM11_ESM.docx]

| **S6 Table** YLDs^a^ and age‑standardized YLDs^a^ rate for foreign body aspiration in 1990 and 2021, and temporal trends | | | | | | |
| --- | --- | --- | --- | --- | --- | --- |
|  | **1990** | |  | **2021** | | **1990-2021 EAPC**^c^**（95%CI**^d^**）** |
|  | **YDLs**^a^ **number (95%UI**^b^**)** | **Age-standardized YDLs**^a^ **rate per 100,000 (95% UI**^b^**)** |  | **YDLs**^a^ **number (95%UI**^b^**)** | **Age-standardized YDLs**^a^ **rate per 100,000 (95% UI**^b^**)** |  |
| **Global** | 136210.56(95652.90-183297.67) | 2.62(1.84-3.51) |  | 95372.10(67472.76-127075.61) | 1.19(0.84-1.59) | -2.58(-2.67 to-2.48) |
| **Sex** |  |  |  |  |  |  |
| **Males** | 68260.15(48035.65-91571.15) | 2.60(1.84-3.47) |  | 47178.48(33309.70-62674.50) | 1.18(0.84-1.57) | -2.59(-2.68,-2.50) |
| **Females** | 67950.41(47076.82-91726.52) | 2.63(1.83-3.53) |  | 48193.62(33875.64-64642.27) | 1.19(0.84-1.60) | -2.56(-2.66,-2.46) |
| **Socio-demographic index** |  |  |  |  |  |  |
| **High SDI**^e^ | 18107.95(13036.79-24070.66) | 1.98(1.43-2.66) |  | 13561.94(9852.05-17489.22) | 1.07(0.77-1.40) | -1.90(-2.01 to-1.78) |
| **High-middle SDI**^e^ | 36128.49(25009.47-48594.79) | 3.37(2.33-4.53) |  | 19488.82(13703.51-26299.37) | 1.37(0.96-1.85) | -3.03(-3.15 to-2.92) |
| **Middle SDI**^e^ | 43674.41(30357.37-59265.68) | 2.62(1.83-3.53) |  | 25335.71(17838.49-34354.07) | 1.01(0.71-1.36) | -3.21(-3.31 to-3.11) |
| **Low-middle SDI**^e^ | 27553.38(19298.17-37487.88) | 2.49(1.76-3.36) |  | 22679.72(15884.15-30245.62) | 1.20(0.84-1.60) | -2.35(-2.47 to-2.22) |
| **Low SDI**^e^ | 10554.03(7373.32-14532.49) | 2.23(1.57-3.03) |  | 14199.45(9801.30-19344.47) | 1.34(0.93-1.80) | -1.61(-1.78 to-1.44) |
| **Region** |  |  |  |  |  |  |
| **East Asia** | 33385.05(22988.96-45191.98) | 2.79(1.93-3.77) |  | 12605.31(8718.66-17352.64) | 0.78(0.53-1.07) | -4.46(-4.72 to-4.20) |
| **Southeast Asia** | 7330.66(5035.68-9982.74) | 1.67(1.16-2.24) |  | 5048.87(3579.46-6892.84) | 0.72(0.51-0.99) | -2.70(-2.88 to-2.51) |
| **Oceania** | 68.04(47.42-92.88) | 1.13(0.80-1.53) |  | 115.59(82.31-155.68) | 0.90(0.64-1.20) | -0.51(-0.70 to-0.32) |
| **Central Asia** | 4341.96(3032.43-6045.37) | 6.45(4.52-8.91) |  | 3566.48(2512.38-4882.98) | 3.71(2.62-5.08) | -2.04(-2.31 to-1.77) |
| **Central Europe** | 7822.66(5507.80-10817.83) | 6.08(4.29-8.43) |  | 3367.88(2372.84-4589.72) | 2.60(1.82-3.54) | -2.72(-2.81 to-2.64) |
| **Eastern Europe** | 9835.26(6921.88-13342.16) | 4.18(2.93-5.69) |  | 5631.84(3965.51-7579.65) | 2.43(1.70-3.28) | -1.92(-2.34 to-1.50) |
| **High-income Asia Pacific** | 2803.84(2059.62-3709.86) | 1.55(1.13-2.06) |  | 2213.89(1653.81-2849.53) | 0.87(0.64-1.15) | -1.73(-1.84 to-1.62) |
| **Australasia** | 428.98(314.13-565.57) | 2.03(1.49-2.70) |  | 507.24(378.18-663.25) | 1.43(1.06-1.89) | -0.74(-0.92 to-0.56) |
| **Western Europe** | 7075.17(5094.64-9442.30) | 1.80(1.29-2.43) |  | 5494.56(4028.19-7221.12) | 1.18(0.86-1.57) | -1.20(-1.32 to-1.08) |
| **Southern Latin America** | 1531.18(1095.35-2074.35) | 3.10(2.22-4.19) |  | 952.21(698.37-1269.30) | 1.37(1.00-1.84) | -2.47(-2.63 to-2.30) |
| **High-income North America** | 3520.76(2562.29-4571.31) | 1.18(0.86-1.55) |  | 3946.98(2911.25-5070.47) | 0.90(0.66-1.16) | -0.90(-1.12 to-0.68) |
| **Caribbean** | 1174.63(812.69-1603.58) | 3.37(2.33-4.58) |  | 884.90(616.61-1193.39) | 1.84(1.28-2.49) | -1.95(-2.04 to-1.86) |
| **Andean Latin America** | 3523.47(2466.35-4882.07) | 9.67(6.80-13.30) |  | 1931.55(1342.77-2567.97) | 2.92(2.03-3.88) | -4.24(-4.40 to-4.09) |
| **Central Latin America** | 6524.33(4529.92-9142.41) | 4.10(2.86-5.67) |  | 4274.02(2921.55-5831.11) | 1.67(1.14-2.28) | -3.18(-3.38 to-2.97) |
| **Tropical Latin America** | 3629.59(2523.53-4880.57) | 2.45(1.71-3.27) |  | 2319.90(1634.81-3097.84) | 0.98(0.69-1.30) | -2.98(-3.09 to-2.88) |
| **North Africa and Middle East** | 7252.05(5093.60-9840.74) | 2.29(1.61-3.08) |  | 6996.37(4889.16-9338.18) | 1.13(0.79-1.50) | -2.38(-2.47 to-2.29) |
| **South Asia** | 26025.15(18235.35-35533.37) | 2.49(1.75-3.36) |  | 21182.19(14750.96-28251.19) | 1.15(0.81-1.54) | -2.42(-2.61 to-2.24) |
| **Central Sub-Saharan Africa** | 1511.24(1046.00-2108.91) | 2.80(1.99-3.87) |  | 2162.49(1498.45-2952.86) | 1.64(1.14-2.23) | -1.82(-2.12 to-1.52) |
| **Eastern Sub-Saharan Africa** | 3356.23(2334.33-4671.29) | 1.81(1.26-2.48) |  | 4247.56(2952.25-5800.39) | 1.03(0.71-1.39) | -1.70(-1.91 to-1.49) |
| **Southern Sub-Saharan Africa** | 845.22(594.32-1146.12) | 1.70(1.20-2.28) |  | 799.05(559.57-1049.44) | 1.00(0.70-1.31) | -1.80(-2.03 to-1.57) |
| **Western Sub-Saharan Africa** | 4225.11(2953.05-5793.46) | 2.35(1.64-3.20) |  | 7123.21(4937.61-9706.48) | 1.59(1.10-2.13) | -1.20(-1.31 to-1.10) |
| **Country** |  |  |  |  |  |  |
| **China** | 32940.71(22678.90-44572.90) | 2.85(1.97-3.85) |  | 12344.74(8538.36-17000.54) | 0.79(0.54-1.09) | -4.49(-4.75 to-4.23) |
| **Democratic People's Republic of Korea** | 188.53(129.64-261.36) | 0.92(0.64-1.28) |  | 148.70(102.52-203.74) | 0.54(0.37-0.75) | -1.71(-1.77 to-1.64) |
| **Taiwan (Province of China)** | 255.81(173.68-359.64) | 1.27(0.87-1.77) |  | 111.87(75.19-155.10) | 0.42(0.28-0.58) | -3.38(-3.63 to-3.13) |
| **Cambodia** | 141.03(96.71-194.29) | 1.51(1.05-2.02) |  | 122.26(86.33-165.48) | 0.75(0.53-1.01) | -2.44(-2.55 to-2.32) |
| **Indonesia** | 2914.24(2005.81-4007.37) | 1.65(1.14-2.24) |  | 1687.03(1196.51-2282.02) | 0.60(0.42-0.81) | -3.19(-3.51 to-2.88) |
| **Lao People's Democratic Republic** | 76.53(53.42-104.19) | 1.97(1.38-2.65) |  | 58.85(40.76-81.14) | 0.83(0.58-1.13) | -3.17(-3.31 to-3.03) |
| **Malaysia** | 342.91(234.81-474.43) | 2.05(1.41-2.78) |  | 327.04(228.21-453.29) | 1.03(0.72-1.42) | -2.14(-2.23 to-2.04) |
| **Maldives** | 4.48(3.10-6.13) | 2.26(1.57-3.05) |  | 3.64(2.57-4.94) | 0.71(0.50-0.96) | -4.00(-4.09 to-3.91) |
| **Myanmar** | 671.69(465.40-907.51) | 1.76(1.22-2.35) |  | 380.14(265.11-515.22) | 0.69(0.48-0.93) | -3.48(-3.66 to-3.30) |
| **Philippines** | 832.18(579.73-1101.61) | 1.50(1.05-1.96) |  | 805.68(573.95-1082.25) | 0.74(0.53-0.99) | -2.16(-2.27 to-2.05) |
| **Sri Lanka** | 252.01(175.73-338.81) | 1.53(1.08-2.05) |  | 210.33(146.98-288.64) | 0.91(0.63-1.25) | -1.99(-2.49 to-1.49) |
| **Thailand** | 881.87(603.67-1222.52) | 1.60(1.10-2.19) |  | 585.77(411.97-789.24) | 0.76(0.53-1.04) | -2.32(-2.44 to-2.20) |
| **Timor-Leste** | 11.57(8.08-15.96) | 1.61(1.13-2.19) |  | 9.99(7.03-13.72) | 0.78(0.55-1.05) | -2.66(-2.76 to-2.56) |
| **Viet Nam** | 1173.68(806.46-1626.79) | 1.85(1.28-2.55) |  | 835.48(580.15-1147.57) | 0.83(0.58-1.15) | -2.59(-2.72 to-2.46) |
| **Fiji** | 9.97(6.93-13.54) | 1.39(0.97-1.88) |  | 10.10(7.07-13.73) | 1.10(0.77-1.49) | -0.63(-0.76 to-0.49) |
| **Kiribati** | 0.58(0.41-0.78) | 0.81(0.58-1.09) |  | 0.55(0.39-0.76) | 0.46(0.33-0.64) | -1.43(-1.66 to-1.19) |
| **Marshall Islands** | 0.53(0.37-0.72) | 1.27(0.89-1.73) |  | 0.48(0.34-0.65) | 0.89(0.63-1.19) | -0.94(-1.18 to-0.70) |
| **Micronesia (Federated States of)** | 1.24(0.86-1.69) | 1.27(0.89-1.71) |  | 0.75(0.53-1.00) | 0.75(0.53-1.01) | -1.55(-1.70 to-1.41) |
| **Papua New Guinea** | 40.61(28.58-55.90) | 1.09(0.77-1.48) |  | 87.09(62.05-117.21) | 0.91(0.65-1.22) | -0.32(-0.53 to-0.11) |
| **Samoa** | 1.90(1.31-2.61) | 1.22(0.84-1.65) |  | 1.35(0.96-1.82) | 0.69(0.49-0.92) | -1.63(-1.79 to-1.47) |
| **Solomon Islands** | 3.32(2.29-4.52) | 1.08(0.75-1.46) |  | 4.70(3.35-6.30) | 0.76(0.54-1.01) | -0.91(-1.15 to-0.67) |
| **Tonga** | 1.07(0.75-1.45) | 1.20(0.84-1.61) |  | 0.76(0.54-1.03) | 0.77(0.55-1.03) | -1.11(-1.32 to-0.89) |
| **Vanuatu** | 1.51(1.05-2.05) | 1.07(0.74-1.44) |  | 2.19(1.54-2.97) | 0.74(0.52-0.99) | -0.88(-1.11 to-0.66) |
| **Armenia** | 262.53(185.67-366.16) | 7.72(5.47-10.75) |  | 116.17(81.43-156.84) | 3.67(2.56-5.00) | -2.67(-2.83 to-2.51) |
| **Azerbaijan** | 558.32(389.05-763.53) | 7.73(5.41-10.54) |  | 561.23(395.01-768.03) | 5.10(3.58-6.99) | -1.58(-1.87 to-1.29) |
| **Georgia** | 261.01(180.26-364.19) | 4.65(3.21-6.51) |  | 153.82(107.30-213.35) | 4.13(2.90-5.77) | 0.11(-0.08 to0.30) |
| **Kazakhstan** | 1298.77(901.54-1820.59) | 7.95(5.52-11.12) |  | 731.85(508.26-1004.69) | 3.86(2.68-5.30) | -3.03(-3.54 to-2.53) |
| **Kyrgyzstan** | 262.41(183.64-365.71) | 6.05(4.27-8.39) |  | 216.71(153.08-301.02) | 3.21(2.26-4.44) | -2.24(-2.57 to-1.91) |
| **Mongolia** | 128.33(89.58-176.93) | 6.14(4.31-8.40) |  | 134.97(94.79-185.55) | 4.08(2.86-5.60) | -1.16(-1.46 to-0.86) |
| **Tajikistan** | 264.54(187.56-365.24) | 5.37(3.83-7.36) |  | 332.02(237.10-455.50) | 3.39(2.43-4.65) | -1.41(-1.68 to-1.15) |
| **Turkmenistan** | 211.77(148.18-295.34) | 5.95(4.19-8.25) |  | 187.65(131.88-256.46) | 3.63(2.55-4.95) | -1.72(-2.00 to-1.44) |
| **Uzbekistan** | 1094.27(765.22-1531.18) | 5.48(3.85-7.58) |  | 1132.06(798.07-1562.47) | 3.30(2.32-4.55) | -1.79(-1.98 to-1.61) |
| **Albania** | 170.31(119.87-235.48) | 5.38(3.80-7.38) |  | 73.02(51.39-100.00) | 2.50(1.75-3.42) | -2.59(-2.64 to-2.54) |
| **Bosnia and Herzegovina** | 297.10(209.54-407.53) | 6.40(4.52-8.78) |  | 126.88(90.01-172.76) | 3.45(2.40-4.72) | -2.17(-2.29 to-2.06) |
| **Bulgaria** | 506.30(355.68-695.62) | 5.60(3.91-7.71) |  | 208.00(145.98-281.71) | 2.81(1.96-3.82) | -2.38(-2.58 to-2.18) |
| **Croatia** | 236.72(163.14-326.04) | 4.62(3.15-6.39) |  | 147.71(103.23-202.63) | 3.12(2.15-4.29) | -0.72(-0.95 to-0.49) |
| **Czechia** | 652.88(455.28-904.96) | 6.11(4.26-8.52) |  | 325.42(229.61-447.12) | 2.71(1.90-3.73) | -2.22(-2.50 to-1.95) |
| **Hungary** | 583.31(409.54-800.91) | 5.32(3.72-7.34) |  | 298.27(208.89-406.91) | 2.72(1.88-3.75) | -2.20(-2.27 to-2.12) |
| **North Macedonia** | 93.85(66.55-129.91) | 4.63(3.28-6.40) |  | 59.03(41.38-80.84) | 2.47(1.73-3.36) | -1.96(-2.04 to-1.89) |
| **Montenegro** | 32.30(22.05-44.99) | 5.09(3.48-7.09) |  | 18.20(12.72-25.01) | 2.75(1.92-3.80) | -2.31(-2.55 to-2.07) |
| **Poland** | 2676.74(1884.74-3724.56) | 6.88(4.84-9.58) |  | 960.67(676.25-1305.44) | 2.21(1.54-3.03) | -3.57(-3.70 to-3.44) |
| **Romania** | 1598.05(1129.02-2215.92) | 6.67(4.71-9.27) |  | 636.42(448.55-867.71) | 2.99(2.08-4.08) | -2.78(-2.87 to-2.70) |
| **Serbia** | 490.11(344.07-683.44) | 4.92(3.44-6.88) |  | 229.76(161.46-316.71) | 2.31(1.62-3.18) | -2.51(-2.62 to-2.40) |
| **Slovakia** | 284.40(199.15-388.76) | 5.24(3.68-7.18) |  | 179.24(125.74-245.21) | 2.95(2.05-4.05) | -1.84(-1.96 to-1.71) |
| **Slovenia** | 75.48(52.02-103.59) | 3.64(2.49-5.02) |  | 56.24(39.56-76.10) | 2.40(1.68-3.29) | -1.12(-1.26 to-0.98) |
| **Belarus** | 487.43(338.20-669.15) | 4.52(3.11-6.23) |  | 260.69(181.61-351.86) | 2.51(1.72-3.40) | -1.97(-2.33 to-1.61) |
| **Estonia** | 99.57(70.49-134.59) | 6.07(4.28-8.25) |  | 35.07(24.50-47.55) | 2.36(1.64-3.22) | -3.43(-3.78 to-3.08) |
| **Latvia** | 111.81(78.92-151.07) | 4.01(2.83-5.47) |  | 44.00(30.98-59.55) | 2.06(1.44-2.80) | -2.16(-2.42 to-1.91) |
| **Lithuania** | 173.21(120.59-235.08) | 4.54(3.14-6.20) |  | 82.86(59.03-112.29) | 2.59(1.80-3.53) | -1.83(-2.13 to-1.53) |
| **Republic of Moldova** | 253.68(176.87-350.09) | 5.67(3.95-7.83) |  | 147.50(104.04-200.13) | 3.61(2.52-4.90) | -1.67(-1.95 to-1.39) |
| **Russian Federation** | 6396.71(4505.69-8619.17) | 4.07(2.86-5.51) |  | 3699.74(2609.91-5001.82) | 2.28(1.59-3.09) | -2.11(-2.60 to-1.62) |
| **Ukraine** | 2312.85(1631.26-3179.41) | 4.23(2.97-5.84) |  | 1361.99(952.25-1820.49) | 2.84(1.98-3.80) | -1.28(-1.53 to-1.02) |
| **Brunei Darussalam** | 3.39(2.47-4.47) | 1.34(0.99-1.76) |  | 4.58(3.35-6.04) | 1.00(0.73-1.32) | -0.80(-0.92 to-0.69) |
| **Japan** | 1930.14(1423.02-2515.84) | 1.42(1.04-1.88) |  | 1686.44(1268.09-2146.50) | 0.89(0.66-1.17) | -1.35(-1.50 to-1.19) |
| **Republic of Korea** | 831.21(608.76-1110.04) | 1.90(1.40-2.54) |  | 472.55(347.16-627.34) | 0.80(0.59-1.07) | -2.71(-2.97 to-2.45) |
| **Singapore** | 39.10(28.55-51.83) | 1.25(0.92-1.66) |  | 50.32(36.61-67.13) | 0.79(0.57-1.05) | -1.33(-1.45 to-1.21) |
| **Australia** | 277.21(205.45-365.84) | 1.58(1.17-2.09) |  | 336.29(251.48-439.89) | 1.12(0.82-1.48) | -1.01(-1.17 to-0.85) |
| **New Zealand** | 151.77(110.94-199.79) | 4.31(3.16-5.71) |  | 170.95(125.38-225.83) | 2.96(2.15-3.96) | -0.13(-0.51 to0.25) |
| **Andorra** | 0.92(0.66-1.25) | 1.63(1.16-2.21) |  | 1.00(0.73-1.34) | 1.09(0.79-1.47) | -1.35(-1.44 to-1.26) |
| **Austria** | 123.71(88.92-166.00) | 1.56(1.12-2.09) |  | 98.27(71.72-131.41) | 1.02(0.74-1.36) | -1.17(-1.30 to-1.03) |
| **Belgium** | 170.88(121.16-230.01) | 1.71(1.21-2.32) |  | 119.22(86.82-158.67) | 0.97(0.70-1.33) | -1.68(-2.06 to-1.31) |
| **Cyprus** | 14.69(10.47-19.81) | 1.87(1.33-2.52) |  | 15.74(11.59-20.95) | 1.08(0.80-1.45) | -1.60(-1.75 to-1.45) |
| **Denmark** | 82.98(59.10-110.56) | 1.57(1.12-2.15) |  | 68.58(49.37-90.80) | 1.13(0.81-1.53) | -0.92(-1.06 to-0.78) |
| **Finland** | 105.61(75.22-141.41) | 2.06(1.46-2.80) |  | 77.22(56.73-102.79) | 1.32(0.97-1.77) | -1.63(-2.22 to-1.04) |
| **France** | 1227.18(888.09-1648.66) | 2.06(1.48-2.80) |  | 883.30(648.86-1173.23) | 1.21(0.88-1.65) | -1.60(-1.73 to-1.47) |
| **Germany** | 1448.24(1030.14-1944.28) | 1.77(1.25-2.40) |  | 909.58(666.28-1195.27) | 1.00(0.73-1.33) | -1.65(-1.81 to-1.49) |
| **Greece** | 226.69(161.59-306.03) | 2.15(1.53-2.92) |  | 106.77(78.09-140.30) | 0.99(0.72-1.30) | -2.65(-2.76 to-2.55) |
| **Iceland** | 4.04(2.89-5.46) | 1.58(1.13-2.13) |  | 4.38(3.19-5.83) | 1.21(0.86-1.63) | -0.68(-0.85 to-0.51) |
| **Ireland** | 57.93(41.19-77.82) | 1.60(1.14-2.16) |  | 56.84(41.14-74.90) | 1.11(0.80-1.47) | -0.98(-1.14 to-0.83) |
| **Israel** | 104.34(74.82-143.66) | 2.12(1.52-2.91) |  | 102.86(74.66-137.66) | 1.06(0.77-1.43) | -2.11(-2.31 to-1.91) |
| **Italy** | 971.55(699.73-1298.48) | 1.66(1.20-2.27) |  | 1207.19(868.53-1645.76) | 2.03(1.44-2.79) | 1.34(0.86 to1.82) |
| **Luxembourg** | 7.02(5.08-9.41) | 1.79(1.29-2.41) |  | 7.17(5.23-9.44) | 1.04(0.76-1.38) | -1.52(-1.66 to-1.38) |
| **Malta** | 5.44(3.92-7.40) | 1.43(1.03-1.95) |  | 4.59(3.38-6.04) | 0.97(0.71-1.29) | -1.06(-1.21 to-0.91) |
| **Netherlands** | 307.92(220.74-421.76) | 2.00(1.44-2.75) |  | 251.84(183.46-339.51) | 1.38(1.00-1.87) | -1.88(-2.19 to-1.56) |
| **Norway** | 64.75(47.28-85.94) | 1.48(1.08-1.99) |  | 51.07(36.97-67.17) | 0.88(0.64-1.18) | -1.47(-1.66 to-1.29) |
| **Portugal** | 232.49(166.00-316.07) | 2.30(1.63-3.14) |  | 117.58(85.44-156.20) | 1.03(0.75-1.42) | -2.88(-3.00 to-2.76) |
| **Spain** | 696.28(492.92-927.47) | 1.76(1.24-2.36) |  | 529.74(383.05-700.30) | 1.05(0.76-1.40) | -1.52(-1.65 to-1.38) |
| **Sweden** | 139.95(101.66-188.23) | 1.57(1.13-2.13) |  | 101.27(73.95-134.32) | 0.92(0.67-1.23) | -1.51(-1.73 to-1.28) |
| **Switzerland** | 90.59(65.81-120.37) | 1.27(0.92-1.71) |  | 91.15(66.55-120.69) | 0.95(0.69-1.27) | -1.17(-1.31 to-1.02) |
| **United Kingdom** | 985.32(721.22-1304.71) | 1.67(1.22-2.24) |  | 683.55(494.16-897.16) | 0.95(0.69-1.27) | -1.76(-1.90 to-1.63) |
| **Argentina** | 1049.68(749.87-1423.48) | 3.18(2.28-4.31) |  | 708.28(520.86-942.27) | 1.52(1.11-2.03) | -2.19(-2.42 to-1.96) |
| **Chile** | 390.84(278.10-528.16) | 2.94(2.10-3.96) |  | 192.69(139.83-257.25) | 1.00(0.72-1.35) | -3.53(-4.36 to-2.69) |
| **Uruguay** | 90.58(65.08-120.39) | 2.89(2.07-3.85) |  | 51.19(37.11-67.81) | 1.44(1.05-1.93) | -2.29(-2.38 to-2.21) |
| **Canada** | 318.82(235.13-413.20) | 1.10(0.81-1.44) |  | 365.91(273.92-473.71) | 0.78(0.58-1.03) | -1.04(-1.12 to-0.96) |
| **United States of America** | 3200.94(2328.47-4162.39) | 1.19(0.87-1.57) |  | 3580.43(2638.92-4604.70) | 0.91(0.66-1.18) | -0.88(-1.12 to-0.63) |
| **Antigua and Barbuda** | 1.35(0.96-1.83) | 2.26(1.60-3.06) |  | 1.12(0.78-1.51) | 1.19(0.83-1.60) | -1.95(-2.04 to-1.86) |
| **Bahamas** | 8.02(5.58-10.94) | 3.17(2.20-4.29) |  | 6.90(4.75-9.35) | 1.72(1.18-2.34) | -1.92(-2.10 to-1.74) |
| **Barbados** | 5.95(4.18-8.05) | 2.30(1.62-3.12) |  | 3.97(2.76-5.33) | 1.23(0.86-1.66) | -1.74(-1.91 to-1.58) |
| **Belize** | 6.31(4.35-8.74) | 3.62(2.50-4.95) |  | 7.60(5.28-10.20) | 1.80(1.25-2.42) | -2.30(-2.37 to-2.23) |
| **Cuba** | 347.57(238.67-473.48) | 3.17(2.17-4.32) |  | 174.05(120.23-234.54) | 1.40(0.96-1.88) | -2.44(-2.58 to-2.30) |
| **Dominica** | 1.92(1.34-2.61) | 2.73(1.91-3.69) |  | 1.13(0.79-1.50) | 1.61(1.13-2.17) | -1.48(-1.63 to-1.33) |
| **Dominican Republic** | 209.55(146.39-288.36) | 3.06(2.15-4.18) |  | 156.43(111.82-208.36) | 1.41(1.01-1.88) | -2.32(-2.44 to-2.19) |
| **Grenada** | 2.76(1.92-3.76) | 3.32(2.31-4.47) |  | 1.89(1.32-2.52) | 1.77(1.23-2.36) | -1.89(-2.15 to-1.63) |
| **Guyana** | 32.03(22.08-43.65) | 4.23(2.93-5.69) |  | 17.18(12.00-23.01) | 2.25(1.57-3.01) | -1.97(-2.06 to-1.89) |
| **Haiti** | 289.58(199.69-397.69) | 4.65(3.23-6.36) |  | 332.16(228.47-460.62) | 2.60(1.80-3.57) | -2.23(-2.61 to-1.85) |
| **Jamaica** | 71.08(48.48-96.94) | 3.09(2.11-4.19) |  | 62.92(42.90-86.10) | 2.20(1.50-3.00) | -0.78(-1.04 to-0.51) |
| **Saint Lucia** | 3.54(2.48-4.83) | 2.72(1.90-3.67) |  | 2.59(1.81-3.46) | 1.36(0.95-1.83) | -2.13(-2.24 to-2.02) |
| **Saint Vincent and the Grenadines** | 3.09(2.17-4.22) | 2.91(2.04-3.94) |  | 2.07(1.43-2.74) | 1.75(1.21-2.31) | -1.62(-1.66 to-1.57) |
| **Suriname** | 14.35(9.95-19.59) | 3.80(2.63-5.14) |  | 12.44(8.63-16.75) | 2.11(1.46-2.84) | -2.06(-2.14 to-1.99) |
| **Trinidad and Tobago** | 34.03(23.68-46.39) | 2.86(1.98-3.86) |  | 21.44(14.96-29.19) | 1.48(1.03-2.04) | -2.13(-2.20 to-2.07) |
| **Bolivia (Plurinational State of)** | 543.67(382.70-749.10) | 8.89(6.34-12.16) |  | 373.92(260.59-499.36) | 3.19(2.23-4.25) | -3.71(-3.84 to-3.58) |
| **Ecuador** | 459.24(318.65-627.83) | 4.93(3.46-6.65) |  | 403.87(284.37-539.14) | 2.24(1.58-2.99) | -2.54(-2.64 to-2.45) |
| **Peru** | 2520.56(1753.24-3488.77) | 12.07(8.46-16.64) |  | 1153.76(804.78-1540.93) | 3.16(2.20-4.22) | -4.80(-4.99 to-4.61) |
| **Colombia** | 964.94(674.56-1337.35) | 3.05(2.13-4.22) |  | 729.27(496.96-1014.31) | 1.45(0.99-2.02) | -2.38(-2.63 to-2.13) |
| **Costa Rica** | 85.32(58.71-119.88) | 2.94(2.04-4.08) |  | 64.16(44.22-88.42) | 1.30(0.90-1.79) | -2.42(-2.56 to-2.27) |
| **El Salvador** | 294.50(205.24-411.35) | 5.85(4.09-8.13) |  | 77.95(54.15-105.92) | 1.20(0.84-1.63) | -5.53(-6.11 to-4.94) |
| **Guatemala** | 597.12(415.57-830.59) | 7.74(5.41-10.72) |  | 401.55(280.73-536.82) | 2.62(1.84-3.48) | -3.71(-3.98 to-3.44) |
| **Honduras** | 147.56(102.37-205.57) | 3.33(2.36-4.57) |  | 172.81(120.71-236.03) | 1.74(1.22-2.37) | -2.10(-2.23 to-1.96) |
| **Mexico** | 3224.88(2232.75-4529.48) | 3.91(2.73-5.42) |  | 1997.98(1399.19-2706.97) | 1.52(1.06-2.07) | -3.58(-4.01 to-3.15) |
| **Nicaragua** | 167.34(115.41-235.05) | 4.58(3.18-6.33) |  | 120.72(82.31-165.56) | 1.83(1.25-2.51) | -3.17(-3.25 to-3.10) |
| **Panama** | 110.19(76.53-153.34) | 4.73(3.29-6.54) |  | 68.68(45.94-94.72) | 1.59(1.06-2.19) | -3.59(-3.69 to-3.50) |
| **Venezuela (Bolivarian Republic of)** | 932.48(640.38-1304.12) | 5.02(3.46-6.99) |  | 640.90(442.04-877.32) | 2.39(1.64-3.28) | -2.40(-2.63 to-2.18) |
| **Brazil** | 3527.85(2453.12-4742.78) | 2.45(1.71-3.26) |  | 2223.98(1566.40-2970.20) | 0.96(0.68-1.29) | -3.01(-3.12 to-2.91) |
| **Paraguay** | 101.73(71.08-136.54) | 2.72(1.90-3.61) |  | 95.92(67.50-129.26) | 1.34(0.95-1.81) | -2.28(-2.35 to-2.21) |
| **Algeria** | 535.42(373.00-725.83) | 2.26(1.58-3.04) |  | 536.62(376.24-732.15) | 1.23(0.86-1.67) | -2.07(-2.14 to-1.99) |
| **Bahrain** | 10.74(7.54-14.55) | 2.13(1.50-2.87) |  | 16.52(11.51-22.24) | 1.02(0.71-1.38) | -2.20(-2.34 to-2.07) |
| **Egypt** | 1166.50(831.31-1574.23) | 2.28(1.62-3.07) |  | 1125.80(804.09-1495.99) | 1.14(0.82-1.50) | -2.25(-2.33 to-2.18) |
| **Iran (Islamic Republic of)** | 1251.32(866.07-1718.45) | 2.34(1.63-3.17) |  | 806.28(557.68-1091.01) | 0.92(0.63-1.24) | -3.06(-3.18 to-2.94) |
| **Iraq** | 375.13(266.43-511.62) | 2.23(1.59-3.00) |  | 431.23(302.62-579.33) | 1.07(0.75-1.44) | -2.75(-2.96 to-2.55) |
| **Jordan** | 82.38(57.39-113.07) | 2.41(1.69-3.25) |  | 142.67(99.32-193.06) | 1.17(0.81-1.58) | -2.60(-2.75 to-2.45) |
| **Kuwait** | 29.94(20.80-39.97) | 1.80(1.24-2.39) |  | 45.35(31.66-61.89) | 0.92(0.64-1.25) | -2.19(-2.30 to-2.08) |
| **Lebanon** | 71.95(49.70-97.86) | 2.48(1.73-3.36) |  | 70.88(48.82-96.02) | 1.24(0.85-1.68) | -2.32(-2.42 to-2.22) |
| **Libya** | 73.99(51.34-100.00) | 1.90(1.32-2.54) |  | 80.89(56.68-109.73) | 1.14(0.80-1.53) | -1.71(-1.78 to-1.64) |
| **Morocco** | 641.59(453.29-875.56) | 2.64(1.87-3.57) |  | 511.94(359.26-675.03) | 1.36(0.96-1.79) | -2.24(-2.39 to-2.10) |
| **Palestine** | 36.94(25.88-50.12) | 2.08(1.46-2.77) |  | 57.32(39.73-76.67) | 1.19(0.82-1.59) | -1.67(-1.84 to-1.50) |
| **Oman** | 28.68(20.28-38.34) | 1.56(1.11-2.06) |  | 37.87(25.93-51.62) | 0.80(0.55-1.09) | -1.98(-2.19 to-1.78) |
| **Qatar** | 9.64(6.79-13.00) | 2.14(1.51-2.87) |  | 25.69(17.57-34.88) | 0.82(0.56-1.11) | -3.37(-3.50 to-3.24) |
| **Saudi Arabia** | 277.27(195.68-379.29) | 1.86(1.33-2.50) |  | 412.85(287.00-550.36) | 1.04(0.72-1.40) | -1.85(-1.98 to-1.72) |
| **Syrian Arab Republic** | 308.90(217.20-421.40) | 2.70(1.91-3.62) |  | 173.93(121.78-231.86) | 1.22(0.85-1.62) | -2.87(-3.00 to-2.73) |
| **Tunisia** | 173.66(120.90-232.71) | 2.18(1.52-2.90) |  | 133.05(92.28-178.01) | 1.08(0.75-1.45) | -2.20(-2.31 to-2.10) |
| **Türkiye** | 1089.42(778.00-1444.12) | 2.01(1.43-2.64) |  | 797.87(558.35-1079.86) | 0.91(0.63-1.23) | -2.50(-2.58 to-2.42) |
| **United Arab Emirates** | 38.49(26.66-52.27) | 2.05(1.43-2.75) |  | 123.44(85.02-167.48) | 1.17(0.81-1.59) | -1.74(-1.89 to-1.60) |
| **Yemen** | 294.01(207.25-401.14) | 2.41(1.70-3.27) |  | 463.62(322.58-623.29) | 1.48(1.04-1.96) | -1.83(-1.94 to-1.72) |
| **Afghanistan** | 245.46(173.65-330.62) | 2.64(1.87-3.53) |  | 440.23(305.57-587.60) | 1.56(1.09-2.06) | -2.09(-2.36 to-1.81) |
| **Bangladesh** | 2763.62(1899.54-3811.63) | 2.69(1.85-3.68) |  | 1750.59(1236.42-2337.19) | 1.09(0.77-1.45) | -3.22(-3.32 to-3.12) |
| **Bhutan** | 13.61(9.43-18.55) | 2.29(1.60-3.11) |  | 7.65(5.38-10.15) | 1.00(0.70-1.33) | -2.80(-2.96 to-2.63) |
| **India** | 20784.81(14593.22-28431.58) | 2.53(1.79-3.42) |  | 16515.56(11509.86-22116.46) | 1.16(0.81-1.56) | -2.42(-2.63 to-2.21) |
| **Nepal** | 413.17(290.41-561.18) | 2.27(1.60-3.06) |  | 331.99(231.40-446.67) | 1.09(0.76-1.46) | -2.44(-2.59 to-2.29) |
| **Pakistan** | 2049.94(1426.70-2782.48) | 2.00(1.41-2.68) |  | 2576.40(1782.99-3487.85) | 1.13(0.79-1.52) | -1.59(-1.80 to-1.38) |
| **Angola** | 290.97(200.70-407.47) | 2.87(2.02-3.98) |  | 370.24(259.21-499.90) | 1.19(0.84-1.59) | -3.20(-3.58 to-2.82) |
| **Central African Republic** | 74.01(50.70-101.89) | 2.71(1.88-3.71) |  | 131.04(91.19-182.61) | 2.37(1.66-3.27) | -0.35(-0.40 to-0.29) |
| **Congo** | 60.87(41.94-85.09) | 2.53(1.76-3.49) |  | 66.34(46.46-89.91) | 1.24(0.86-1.67) | -2.47(-2.68 to-2.26) |
| **Democratic Republic of the Congo** | 1049.02(729.87-1457.03) | 2.81(1.99-3.88) |  | 1560.18(1086.53-2137.74) | 1.80(1.25-2.46) | -1.46(-1.77 to-1.15) |
| **Equatorial Guinea** | 13.90(9.62-19.44) | 3.35(2.31-4.62) |  | 12.83(8.77-17.51) | 0.85(0.59-1.16) | -4.94(-5.21 to-4.68) |
| **Gabon** | 22.47(15.42-30.83) | 2.32(1.60-3.16) |  | 21.86(15.31-29.56) | 1.21(0.85-1.64) | -1.92(-2.12 to-1.71) |
| **Burundi** | 88.05(60.58-122.61) | 1.62(1.12-2.23) |  | 150.73(104.78-206.31) | 1.18(0.82-1.60) | -0.81(-1.03 to-0.60) |
| **Comoros** | 8.04(5.49-11.18) | 1.80(1.24-2.49) |  | 8.72(6.08-11.98) | 1.18(0.82-1.61) | -1.26(-1.34 to-1.18) |
| **Djibouti** | 6.18(4.25-8.53) | 1.52(1.05-2.08) |  | 11.20(7.85-15.18) | 0.89(0.63-1.20) | -1.62(-1.80 to-1.44) |
| **Eritrea** | 52.33(36.30-72.45) | 1.57(1.09-2.16) |  | 68.26(47.40-92.92) | 1.06(0.73-1.42) | -1.11(-1.26 to-0.97) |
| **Ethiopia** | 1091.31(759.56-1522.61) | 2.21(1.55-3.04) |  | 1040.50(723.65-1416.18) | 0.99(0.69-1.34) | -2.51(-2.86 to-2.15) |
| **Kenya** | 403.99(283.89-554.08) | 1.82(1.28-2.48) |  | 477.10(331.40-650.30) | 0.96(0.67-1.30) | -1.48(-1.75 to-1.20) |
| **Madagascar** | 194.27(135.52-273.43) | 1.68(1.17-2.33) |  | 309.28(213.92-425.68) | 1.12(0.77-1.52) | -1.30(-1.46 to-1.15) |
| **Malawi** | 148.02(100.74-204.39) | 1.55(1.05-2.12) |  | 187.31(128.98-254.96) | 0.99(0.68-1.33) | -1.26(-1.41 to-1.11) |
| **Mauritius** | 16.88(11.52-23.29) | 1.56(1.07-2.13) |  | 14.83(10.40-20.13) | 1.10(0.76-1.50) | -1.04(-1.32 to-0.75) |
| **Mozambique** | 230.14(157.08-317.89) | 1.79(1.22-2.43) |  | 339.95(229.67-469.53) | 1.11(0.77-1.52) | -1.47(-1.56 to-1.39) |
| **Rwanda** | 121.53(83.50-168.87) | 1.73(1.18-2.36) |  | 128.45(88.04-175.72) | 0.99(0.69-1.35) | -1.75(-1.98 to-1.52) |
| **Seychelles** | 0.98(0.68-1.33) | 1.39(0.96-1.88) |  | 0.81(0.57-1.09) | 0.74(0.52-1.00) | -1.90(-2.01 to-1.78) |
| **Somalia** | 131.59(90.69-182.32) | 1.71(1.18-2.33) |  | 248.34(173.62-342.92) | 1.22(0.85-1.66) | -1.04(-1.19 to-0.88) |
| **United Republic of Tanzania** | 426.89(291.93-583.90) | 1.70(1.17-2.30) |  | 613.16(425.97-848.64) | 1.08(0.76-1.48) | -1.36(-1.50 to-1.22) |
| **Uganda** | 252.08(172.87-349.42) | 1.49(1.02-2.03) |  | 393.39(271.32-538.04) | 0.95(0.66-1.30) | -1.35(-1.54 to-1.17) |
| **Zambia** | 117.33(80.81-162.26) | 1.49(1.03-2.03) |  | 173.29(119.11-235.41) | 0.90(0.62-1.22) | -1.51(-1.71 to-1.31) |
| **Botswana** | 18.34(12.78-24.47) | 1.49(1.06-1.98) |  | 22.52(15.61-29.83) | 0.93(0.65-1.23) | -1.33(-1.45 to-1.22) |
| **Lesotho** | 24.08(16.93-32.73) | 1.68(1.18-2.25) |  | 25.74(18.05-34.21) | 1.37(0.96-1.80) | -0.36(-0.53 to-0.20) |
| **Namibia** | 20.33(14.08-27.26) | 1.57(1.10-2.08) |  | 25.27(17.59-33.40) | 1.06(0.74-1.40) | -1.12(-1.24 to-1.00) |
| **South Africa** | 629.25(443.19-852.53) | 1.76(1.24-2.37) |  | 498.72(351.28-660.11) | 0.87(0.61-1.15) | -2.53(-2.85 to-2.20) |
| **Eswatini** | 10.77(7.46-14.66) | 1.46(1.02-1.96) |  | 13.34(9.39-17.80) | 1.17(0.83-1.54) | -0.56(-0.70 to-0.41) |
| **Zimbabwe** | 142.46(97.67-192.92) | 1.51(1.07-2.02) |  | 213.46(150.36-285.37) | 1.47(1.04-1.94) | 0.59(0.31 to0.87) |
| **Benin** | 103.44(71.68-141.95) | 2.34(1.63-3.17) |  | 182.90(128.53-244.58) | 1.55(1.09-2.04) | -1.26(-1.33 to-1.18) |
| **Burkina Faso** | 184.82(127.61-253.70) | 2.08(1.44-2.81) |  | 327.60(225.21-447.21) | 1.56(1.07-2.12) | -0.81(-0.90 to-0.71) |
| **Cameroon** | 210.75(145.05-289.63) | 2.19(1.53-2.99) |  | 504.77(350.93-690.66) | 1.75(1.20-2.34) | -0.56(-0.73 to-0.39) |
| **Cabo Verde** | 11.13(7.67-15.19) | 3.36(2.33-4.53) |  | 9.84(6.90-13.10) | 1.76(1.23-2.34) | -2.27(-2.41 to-2.14) |
| **Chad** | 123.57(85.98-168.17) | 2.25(1.56-3.04) |  | 291.81(206.83-395.42) | 1.89(1.33-2.51) | -0.45(-0.58 to-0.32) |
| **Côte d'Ivoire** | 245.68(168.83-332.46) | 2.15(1.50-2.89) |  | 411.56(286.08-556.04) | 1.62(1.13-2.16) | -0.71(-0.82 to-0.60) |
| **Gambia** | 19.78(13.64-27.13) | 2.19(1.53-2.97) |  | 34.02(23.87-45.61) | 1.59(1.13-2.10) | -0.87(-0.98 to-0.76) |
| **Ghana** | 249.23(173.85-343.51) | 1.78(1.25-2.42) |  | 341.41(237.55-455.56) | 1.05(0.73-1.38) | -1.40(-1.60 to-1.20) |
| **Guinea** | 135.30(94.10-186.23) | 2.42(1.68-3.31) |  | 220.37(151.59-297.49) | 1.84(1.28-2.44) | -0.73(-0.83 to-0.63) |
| **Guinea-Bissau** | 23.68(16.29-32.47) | 2.49(1.73-3.36) |  | 29.31(20.53-39.62) | 1.58(1.11-2.09) | -1.45(-1.58 to-1.33) |
| **Liberia** | 60.67(42.26-83.26) | 2.63(1.83-3.58) |  | 78.63(55.68-104.73) | 1.61(1.14-2.11) | -1.80(-1.87 to-1.73) |
| **Mali** | 211.70(144.53-292.29) | 2.66(1.84-3.61) |  | 432.09(300.80-596.34) | 2.01(1.40-2.72) | -0.82(-0.90 to-0.74) |
| **Mauritania** | 40.77(28.13-55.42) | 2.16(1.50-2.87) |  | 54.07(37.61-73.14) | 1.36(0.96-1.82) | -1.31(-1.57 to-1.05) |
| **Niger** | 193.41(136.30-268.09) | 2.66(1.87-3.61) |  | 392.88(276.09-538.97) | 1.86(1.32-2.46) | -1.22(-1.27 to-1.18) |
| **Nigeria** | 2070.04(1448.64-2831.22) | 2.46(1.72-3.34) |  | 3321.27(2289.05-4570.59) | 1.55(1.08-2.10) | -1.49(-1.60 to-1.37) |
| **Sao Tome and Principe** | 3.72(2.59-5.10) | 3.28(2.28-4.46) |  | 4.23(2.96-5.70) | 2.08(1.46-2.78) | -1.64(-1.75 to-1.54) |
| **Senegal** | 162.99(114.26-225.52) | 2.33(1.64-3.15) |  | 216.16(150.22-287.92) | 1.50(1.05-1.97) | -1.25(-1.39 to-1.12) |
| **Sierra Leone** | 98.31(68.45-134.46) | 2.52(1.75-3.41) |  | 153.23(106.12-208.82) | 1.88(1.31-2.52) | -0.85(-1.00 to-0.70) |
| **Togo** | 75.98(52.68-104.17) | 2.27(1.57-3.07) |  | 116.94(80.84-156.32) | 1.55(1.09-2.05) | -1.04(-1.18 to-0.90) |
| **American Samoa** | 0.59(0.41-0.80) | 1.34(0.93-1.79) |  | 0.55(0.39-0.73) | 1.10(0.78-1.47) | -0.31(-0.53 to-0.09) |
| **Bermuda** | 1.45(1.01-1.96) | 2.36(1.64-3.19) |  | 0.80(0.56-1.09) | 1.13(0.78-1.54) | -2.08(-2.26 to-1.90) |
| **Cook Islands** | 0.23(0.16-0.31) | 1.25(0.88-1.69) |  | 0.13(0.09-0.17) | 0.67(0.47-0.91) | -1.70(-1.86 to-1.55) |
| **Greenland** | 0.92(0.67-1.20) | 1.73(1.26-2.23) |  | 0.58(0.43-0.76) | 1.02(0.75-1.32) | -1.89(-1.96 to-1.81) |
| **Guam** | 1.27(0.89-1.72) | 0.95(0.67-1.28) |  | 1.02(0.72-1.39) | 0.62(0.43-0.85) | -1.05(-1.23 to-0.88) |
| **Monaco** | 0.50(0.36-0.67) | 1.59(1.13-2.16) |  | 0.45(0.32-0.60) | 1.14(0.82-1.53) | -1.06(-1.13 to-0.99) |
| **Nauru** | 0.08(0.05-0.11) | 0.83(0.58-1.11) |  | 0.06(0.04-0.08) | 0.60(0.42-0.81) | -0.92(-1.40 to-0.45) |
| **Niue** | 0.02(0.02-0.03) | 1.09(0.77-1.47) |  | 0.01(0.01-0.02) | 0.67(0.47-0.90) | -1.48(-1.64 to-1.31) |
| **Northern Mariana Islands** | 0.46(0.32-0.62) | 1.04(0.72-1.41) |  | 0.42(0.29-0.56) | 0.83(0.59-1.13) | -0.16(-0.37 to0.06) |
| **Palau** | 0.19(0.13-0.25) | 1.26(0.88-1.68) |  | 0.16(0.11-0.21) | 0.82(0.58-1.10) | -1.27(-1.35 to-1.19) |
| **Puerto Rico** | 98.23(66.32-135.01) | 2.71(1.83-3.73) |  | 48.23(32.98-65.56) | 1.28(0.87-1.76) | -2.28(-2.38 to-2.18) |
| **Saint Kitts and Nevis** | 0.91(0.63-1.24) | 2.24(1.56-3.04) |  | 0.67(0.46-0.91) | 1.09(0.75-1.47) | -2.09(-2.21 to-1.98) |
| **San Marino** | 0.34(0.25-0.47) | 1.39(1.01-1.92) |  | 0.36(0.26-0.48) | 1.05(0.75-1.38) | -0.82(-0.96 to-0.68) |
| **Tokelau** | 0.02(0.02-0.03) | 1.52(1.06-2.06) |  | 0.01(0.01-0.02) | 0.82(0.59-1.12) | -1.77(-1.95 to-1.60) |
| **Tuvalu** | 0.11(0.08-0.15) | 1.24(0.86-1.66) |  | 0.08(0.06-0.11) | 0.67(0.48-0.89) | -1.79(-1.93 to-1.66) |
| **United States Virgin Islands** | 3.13(2.16-4.25) | 2.98(2.05-4.04) |  | 1.36(0.96-1.83) | 1.45(1.02-1.95) | -2.08(-2.24 to-1.93) |
| **South Sudan** | 82.07(57.35-111.41) | 1.44(1.00-1.94) |  | 94.19(65.21-128.67) | 1.01(0.70-1.37) | -1.06(-1.19 to-0.93) |
| **Sudan** | 506.64(358.16-693.96) | 2.71(1.92-3.66) |  | 555.77(385.53-741.02) | 1.35(0.94-1.79) | -2.51(-2.64 to-2.39) |

^a^Years lived with disability, ^b^Uncertainty interval, ^c^Estimated annual percentage change, ^d^Confidence interval, ^e^Sociodemographic indices,
